# Estimates of global, regional, and national morbidity, mortality, and aetiologies of diarrhoeal diseases: a systematic analysis for the Global Burden of Disease Study 2015

**DOI:** 10.1016/S1473-3099(17)30276-1

**Published:** 2017-09

**Authors:** GBD Diarrhoeal Diseases Collaborators, Christopher Troeger, Mohammad Forouzanfar, Puja C Rao, Ibrahim Khalil, Alexandria Brown, Robert C Reiner, Nancy Fullman, Robert L Thompson, Amanuel Abajobir, Muktar Ahmed, Mulubirhan Assefa Alemayohu, Nelson Alvis-Guzman, Azmeraw T Amare, Carl Abelardo Antonio, Hamid Asayesh, Euripide Avokpaho, Ashish Awasthi, Umar Bacha, Aleksandra Barac, Balem Demtsu Betsue, Addisu Shunu Beyene, Dube Jara Boneya, Deborah Carvalho Malta, Lalit Dandona, Rakhi Dandona, Manisha Dubey, Babak Eshrati, Joseph R A Fitchett, Tsegaye Tewelde Gebrehiwot, Gessessew Buggsa Hailu, Masako Horino, Peter J Hotez, Tariku Jibat, Jost B Jonas, Amir Kasaeian, Niranjan Kissoon, Karen Kotloff, Ai Koyanagi, G Anil Kumar, Rajesh Kumar Rai, Aparna Lal, Hassan Magdy Abd El Razek, Mubarek Abera Mengistie, Christine Moe, George Patton, James A Platts-Mills, Mostafa Qorbani, Usha Ram, Hirbo Shore Roba, Juan Sanabria, Benn Sartorius, Monika Sawhney, Mika Shigematsu, Chandrashekhar Sreeramareddy, Soumya Swaminathan, Bemnet Amare Tedla, Roman Topor-Madry Jagiellonian, Kingsley Ukwaja, Andrea Werdecker, Marc-Alain Widdowson, Naohiro Yonemoto, Maysaa El Sayed Zaki, Stephen S Lim, Mohsen Naghavi, Theo Vos, Simon I Hay, Christopher J L Murray, Ali H Mokdad

## Abstract

**Background:**

The Global Burden of Diseases, Injuries, and Risk Factors Study 2015 (GBD 2015) provides an up-to-date analysis of the burden of diarrhoeal diseases. This study assesses cases, deaths, and aetiologies spanning the past 25 years and informs the changing picture of diarrhoeal disease worldwide.

**Methods:**

We estimated diarrhoeal mortality by age, sex, geography, and year using the Cause of Death Ensemble Model (CODEm), a modelling platform shared across most causes of death in the GBD 2015 study. We modelled diarrhoeal morbidity, including incidence and prevalence, using a meta-regression platform called DisMod-MR. We estimated aetiologies for diarrhoeal diseases using a counterfactual approach that incorporates the aetiology-specific risk of diarrhoeal disease and the prevalence of the aetiology in diarrhoea episodes. We used the Socio-demographic Index, a summary indicator derived from measures of income per capita, educational attainment, and fertility, to assess trends in diarrhoeal mortality. The two leading risk factors for diarrhoea—childhood malnutrition and unsafe water, sanitation, and hygiene—were used in a decomposition analysis to establish the relative contribution of changes in diarrhoea disability-adjusted life-years (DALYs).

**Findings:**

Globally, in 2015, we estimate that diarrhoea was a leading cause of death among all ages (1·31 million deaths, 95% uncertainty interval [95% UI] 1·23 million to 1·39 million), as well as a leading cause of DALYs because of its disproportionate impact on young children (71·59 million DALYs, 66·44 million to 77·21 million). Diarrhoea was a common cause of death among children under 5 years old (499 000 deaths, 95% UI 447 000–558 000). The number of deaths due to diarrhoea decreased by an estimated 20·8% (95% UI 15·4–26·1) from 2005 to 2015. Rotavirus was the leading cause of diarrhoea deaths (199 000, 95% UI 165 000–241 000), followed by *Shigella* spp (164 300, 85 000–278 700) and *Salmonella* spp (90 300, 95% UI 34 100–183 100). Among children under 5 years old, the three aetiologies responsible for the most deaths were rotavirus, *Cryptosporidium* spp, and *Shigella* spp. Improvements in safe water and sanitation have decreased diarrhoeal DALYs by 13·4%, and reductions in childhood undernutrition have decreased diarrhoeal DALYs by 10·0% between 2005 and 2015.

**Interpretation:**

At the global level, deaths due to diarrhoeal diseases have decreased substantially in the past 25 years, although progress has been faster in some countries than others. Diarrhoea remains a largely preventable disease and cause of death, and continued efforts to improve access to safe water, sanitation, and childhood nutrition will be important in reducing the global burden of diarrhoea.

**Funding:**

Bill & Melinda Gates Foundation.

## Introduction

In 2015, diarrhoea caused more than 1·3 million deaths globally and was the fourth leading cause of death among children younger than 5 years.^1^ Although the burden is greatest in low-income populations with poor access to safe water, sanitation, and urgent medical care, acute infectious diarrhoea is also a common cause of outpatient visits and hospital admissions in high-income regions and is an important health problem globally. Prevention and treatment of diarrhoea is challenging because of pervasive infrastructural, political, and socioeconomic barriers, including access to safe water and sanitation, education, nutrition, and access to health care.^2^

Estimates of the burden of diarrhoea and its aetiologies are being produced annually as part of the Global Burden of Diseases, Injuries, and Risk Factors Study 2015 (GBD 2015), which provides a unique source for tracking progress in reducing morbidity and mortality due to diarrhoea. The findings of this study quantify the burden of childhood diarrhoea, which will help to track progress toward achieving Sustainable Development Goal (SDG) 3 of ensuring healthy lives and promoting wellbeing for all at all ages.

Here, we present the results of GBD 2015 for diarrhoea and 13 aetiologies covering deaths and disability-adjusted life-years (DALYs) for 195 countries or territories from 1990 to 2015 by age and sex. Because of the disproportionate burden of diarrhoea in children younger than 5 years, our results and discussion focus on this age group.

**Research in context**

**Evidence before this study**

This manuscript builds on previous GBD publications with updated data and methods. Diarrhoeal diseases are a leading cause of morbidity and mortality, especially in children younger than 5 years, and the global burden has been estimated by several groups, including the Maternal and Child Health Epidemiology Estimation group and the Global Burden of Disease Study (GBD) 2013. Diarrhoea mortality has declined substantially since 1990, but morbidity has not declined as rapidly. Diarrhoeal mortality attributable to aetiologies has mainly been based on categorical attribution from non-molecular diagnostic methods with low overall attribution.

**Added value of this study**

This study provides a comprehensive assessment of diarrhoea burden based on the findings of GBD 2015, including new and more robust evidence on the mortality, morbidity, and risk factors associated with diarrhoea and 13 aetiologies and is the first cause-specific description of diarrhoea from the GBD group. Moreover, it introduces molecular diagnostic case definitions for diarrhoeal aetiologies. In addition to descriptions of trends in morbidity and mortality, this analysis uses a Socio-demographic Index to relate changes in diarrhoea burden to demographic transitions and assesses the effect of changing population characteristics and risk factor exposure to decompose trends in diarrhoea mortality.

**Implications of all the available evidence**

This study provides a detailed picture of the decreasing diarrhoeal burden over time and 13 aetiologies across all geographies while relating these trends to changes in risk factor exposure. This work allows for an in-depth understanding of national health challenges and areas for intervention. The findings will have great implications for strategies and programmes to address the burden of diarrhoea at the global, country, and local level.

## Methods

### Overview

The general methods for GBD 2015 and earlier GBD iterations have been described previously.^1^, ^3^ Diarrhoea burden is measured with several metrics: deaths, episodes, and DALYs. DALYs are a sum of years of life lost (YLLs) because of premature death and years lived with disability (YLDs). DALYs for diarrhoea reflect the acute outcomes of the disease. We produced all estimates by year and by age, for both sexes, and for all countries. Flowcharts and a detailed description for each step of the estimation process are provided in the appendix, in accordance with the Guidelines for Accurate and Transparent Health Estimates Reporting (GATHER). Code for each step of the estimation process is available on GitHub.

We used 1000 draws from a posterior distribution of each parameter and estimated value to retain uncertainty throughout the estimation process. The results are presented as mean values with 95% uncertainty intervals (95% UIs) representing the 2·5th and 97·5th percentiles of the distribution.

### Mortality

The Global Burden of Disease Cause of Death (CoD) database consists of all available data from surveillance systems, vital registration systems, and verbal autopsy (appendix p 2). Raw data are processed to reconcile disparate coding schemes (such as the International Classification of Diseases [ICD] 9 and 10) and to redistribute poorly coded causes of death, among other corrections.^4^

We estimated diarrhoeal disease mortality in the Cause of Death Ensemble model framework (CODEm).^1^, ^5^ CODEm is a spatiotemporal modelling platform that produces a wide range of sub-models based on CoD data and space–time covariates. Covariates are selected independently for each sub-model, and the selection is based on an algorithm that captures plausible relationships between the covariates and diarrhoeal mortality and provides a diverse set of plausible models (appendix p 5). These sub-models are evaluated using in-sample and out-of-sample validity, root mean square error, and input datapoint coverage. The best performing sub-models provide a greater relative number of draws to the final 1000 draws for the model of diarrhoea mortality. We assessed our diarrhoeal disease CoD models using in-sample and out-of-sample predictive performance. This modelling process is described in more detail in the appendix (p 3).

There is a final step in modelling causes of death called CoDCorrect, which ensures internal consistency among all causes of death in GBD. Like all mortality models in GBD, diarrhoea mortality models are single-cause. The sum of all mortality models must be equal to the all-cause mortality envelope. We corrected diarrhoea mortality estimates, and other causes of mortality, by rescaling them in accordance with the uncertainty around the cause-specific mortality rate.

### Morbidity

Diarrhoeal cases are defined as passing three or more loose stools in a 24 h period.^6^ Input data for these models were from population representative surveys, hospital inpatient and outpatient records (ICD9 codes 001–009.9 and ICD10 codes A00–A09), health care utilisation (USA only), excess mortality from the GBD 2015 CoD estimates for diarrhoea, and a systematic literature review of cohort and cross-sectional studies (appendix p 9).

We estimated diarrhoeal disease incidence and prevalence for each location, age, and sex from 1990 to 2015 using an age-integrating meta–regression tool called DisMod-MR 2.1 (DisMod) which has been described in more detail previously^3^, ^7^, ^8^ and in the appendix (p 7). DisMod adjusts for variations in study methods between data sources and enforces consistency between data for different parameters such as incidence and prevalence. Incidence, prevalence, remission, and excess mortality are dependent in a compartmental model of disease progression. Geospatial priors, space–time covariates, random effects, and input data predict incidence and prevalence of diarrhoea. The tool evaluates epidemiological data on diarrhoea burden through a geographical cascade of four levels from global, where fixed effects for covariates are established, to the smallest geographical estimation unit. Outputs from larger geographical units of the cascade are used as priors in smaller subunits within the same geography. DisMod uses geographically representative random effects to produce estimates in areas with sparse data.^3^

Diarrhoeal diseases have three severity levels: mild, moderate, and severe (appendix p 10). To estimate YLDs from diarrhoeal diseases, we calculated a disability weight for each of the diarrhoea severity levels and the percentage of cases that fall into each state (appendix p 10). DALYs are the sum of YLLs and YLDs and represent the cumulative burden of disease due to diarrhoea.^9^

### Aetiologies

We estimated diarrhoeal disease aetiologies separately from overall diarrhoea mortality. Aetiologies included enteric adenovirus (serotypes 40 and 41), *Aeromaons* spp, *Entamoeba histolytica* (amoebiasis), *Campylobacter* spp enteritis (*Campylobacter*), cryptosporidiosis (*Cryptosporidium* spp), typical enteropathogenic *Escherichia coli* (tEPEC), enterotoxigenic *E coli* (ETEC; both ST and LT), norovirus, non-typhoidal *Salmonella* spp, rotaviral enteritis (rotavirus), shigellosis (*Shigella* spp), *Vibrio cholerae* (cholera), and *Clostridium difficile*. The modelling strategy for diarrhoeal aetiologies is described in more detail in the appendix (pp 11–26).

We used a counterfactual approach that allows for interactions between pathogens and accounts for the distribution of pathogens in healthy individuals. We estimated a population attributable fraction (PAF), for each aetiology, which is the product of pathogen presence and the odds ratio (OR) of diarrhoea given its detection:^10^

$$PAF=\mathrm{Pr}oportion*(1-\frac{1}{OR})$$

Where *OR* is the OR of diarrhoea given the presence of a pathogen and *Proportion* is the modelled proportion of diarrhoea episodes where the pathogen is present.

For GBD 2015, we used a systematic reanalysis of the Global Enteric Multicenter Study (GEMS)^11^, ^12^ that used quantitative polymerase chain reaction (qPCR) as the diagnostic tool for pathogen detection to estimate the ORs of diarrhoea given pathogen detection. GEMS is a seven site, case-control study of moderate-to-severe diarrhoea in children younger than 5 years in south Asia and sub-Saharan Africa. Validation studies have shown that the use of molecular diagnostics is more sensitive than is traditional laboratory diagnostic methods for the detection of diarrhoeal pathogens.^13^, ^14^ We used a mixed-effects conditional logistic regression model, matching for case-control pairs, random effects for GEMS sites, and accounting for all pathogens to calculate the OR by age for each of our aetiologies. OR did not vary by time or geography, a change from GBD 2013 when we used region-specific ORs.^15^

We did a systematic literature review of the proportion of diarrhoea cases that tested positive for each aetiology (appendix p 19) and used the meta-regression tool DisMod-MR to model the proportion of positive diarrhoea cases, for each aetiology separately, by location, year, age, and sex. We used rotavirus vaccine coverage as a covariate in the rotavirus proportion model only. Because most of the studies published on this topic used a case definition based on non-molecular diagnostics, we used the sensitivity and specificity of these methods compared with our qPCR case definition (appendix p 15) to correct the proportion estimates for exposure misclassification due to diagnostic error.^16^, ^17^

$$\mathrm{Pr}oportion_{True}=\frac{\left(\mathrm{Pr}oportion_{observed}*Specificity-1\right)}{\left(Sensitivity+Specificity-1\right)}$$

We estimated a distinct fatal and non-fatal PAF for each aetiology assuming that diarrhoea episodes with hospital admission are a reasonable proxy for the cause of fatal cases since data on the cause of diarrhoea mortality after death were not available. Finally, we multiplied PAFs by the fatal and non-fatal diarrhoea envelopes to establish cases and deaths by aetiology.

We modelled *V cholerae* independently from the other aetiologies because of its epidemic tendency and imperfect reporting frequency. We used a systematic literature review to estimate the expected number of cholera cases for each country–year. We compared this expected number of cholera cases to the number reported to WHO and used this under-reporting fraction to correct the cholera case notification data for all countries.^18^ We modelled the case fatality ratio of cholera using DisMod-MR and applied these values to the cholera case envelope to estimate the number of cholera deaths.

We also modelled *C difficile* independently from the aetiologies because it was not included as a pathogen in GEMS. We did a systematic literature review for the prevalence and incidence of *C difficile* and used inpatient and outpatient hospital visits coded for *C difficile*. We modelled the natural history of *C difficile* infection, including incidence and mortality, in DisMod-MR for each location, year, age, and sex.

### Risk factor decomposition

Risk factors for diarrhoeal diseases are modelled as part of GBD 2015 and have been described in detail previously.^19^, ^20^ Risk factors are different from diarrhoea model covariates. Briefly, risk factors also follow a PAF counterfactual approach in which the prevalence of exposure is modelled from scientific literature and population representative surveys, and the relative risk of diarrhoea given risk exposure is taken from published meta-analyses. Although there are ten total risk factors for diarrhoea in GBD 2015, we used only the two leading risk factors for diarrhoea DALYs, unsafe water and sanitation and childhood undernutrition, in a decomposition analysis of the change in DALYs due to diarrhoea from 2005 to 2015. The decomposition is of five factors that contribute interdependently to diarrhoea burden, including undernutrition exposure, unsafe water or sanitation exposure, population growth, population ageing, and the underlying rate of DALYs from diarrhoea unexplained by the other factors. A combinatorial process calculates the relative contribution of each of these five factors to the change in diarrhoea DALYs.^20^, ^21^ These analyses are not done at the draw level so uncertainty is not propogated through the risk factor decomposition.

### Burden transition with development

Based on methods used to construct the Human Development Index, GBD 2015 used the Socio-demographic Index (SDI), a summary measure of development based on lag-dependent income per capita, average educational attainment, and total fertility rate.^1^, ^22^ We used age-standardised estimates of the diarrhoea mortality rate for each year and most detailed geographical location to calculate the relationship between SDI and diarrhoea mortality using a simple least-squares regression with a cubic spline.

### Role of the funding source

The funder of the study had no role in study design, data collection, data analysis, data interpretation, or writing of the report. The corresponding author had full access to all data in the study and had final responsibility for the decision to submit for publication.

## Results

**Figure 1:**
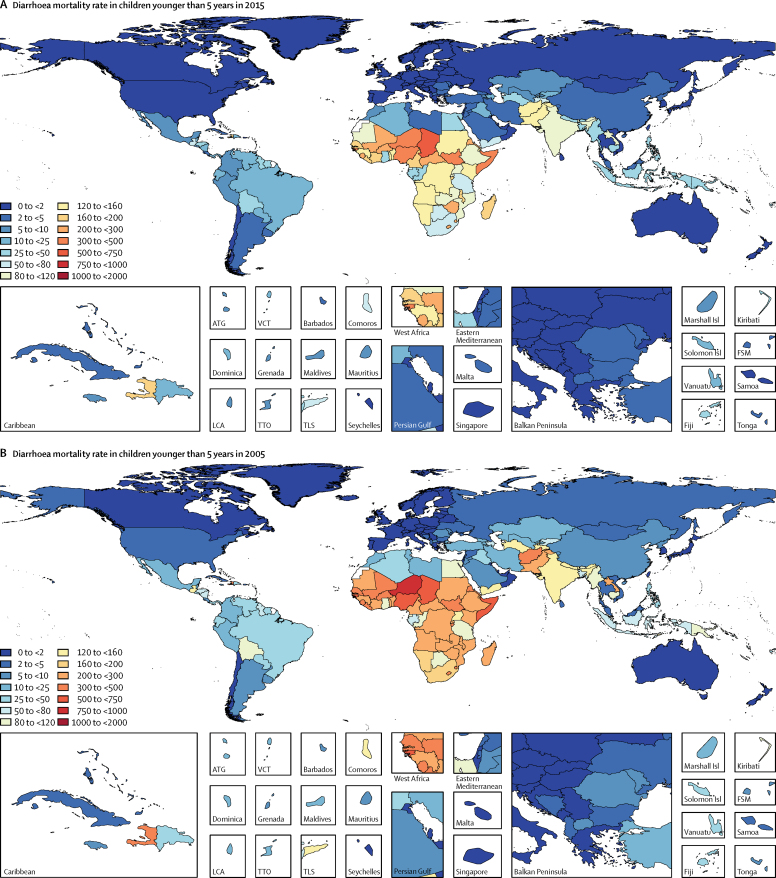
Under-5 diarrhoea mortality rate per 100 000 population (A) Under-5 mortality in 2015. (B) Under-5 mortality in 2005. ATG=Antigua and Barbuda. VCT=Saint Vincent and the Grenadines. LCA=Saint Lucia. TTO=Trinidad and Tobago. TLS=Timor-Leste. FSM=Federated States of Micronesia.

**Figure 2:**
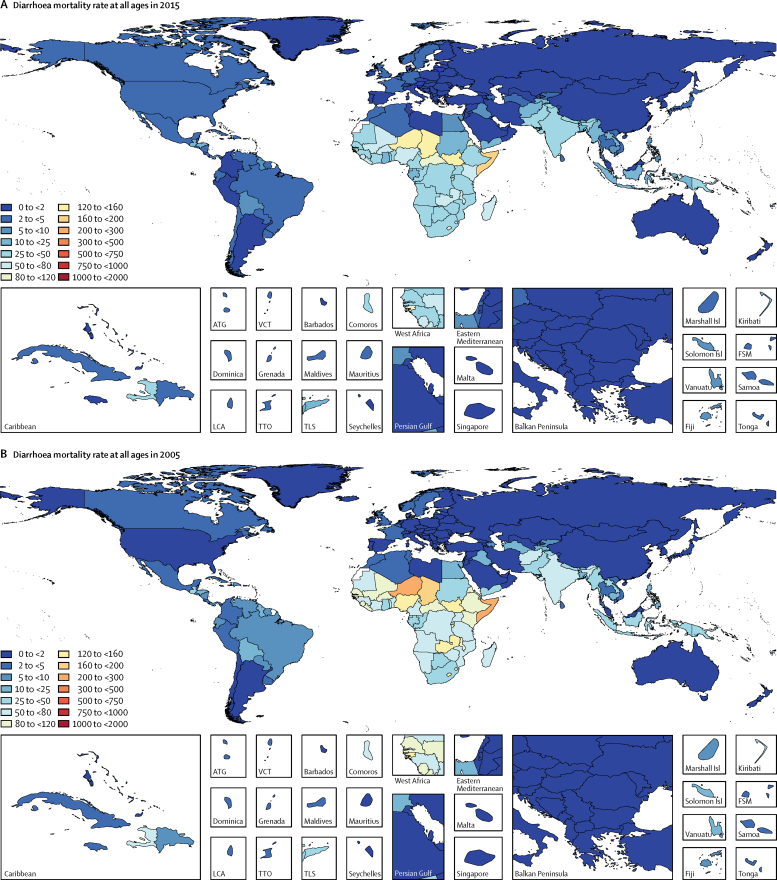
All ages diarrhoea mortality rate per 100 000 population (A) All-ages mortality in 2015. (B) All-ages mortality in 2005. ATG=Antigua and Barbuda. VCT=Saint Vincent and the Grenadines. LCA=Saint Lucia. TTO=Trinidad and Tobago. TLS=Timor-Leste. FSM=Federated States of Micronesia.

**Figure 3:**
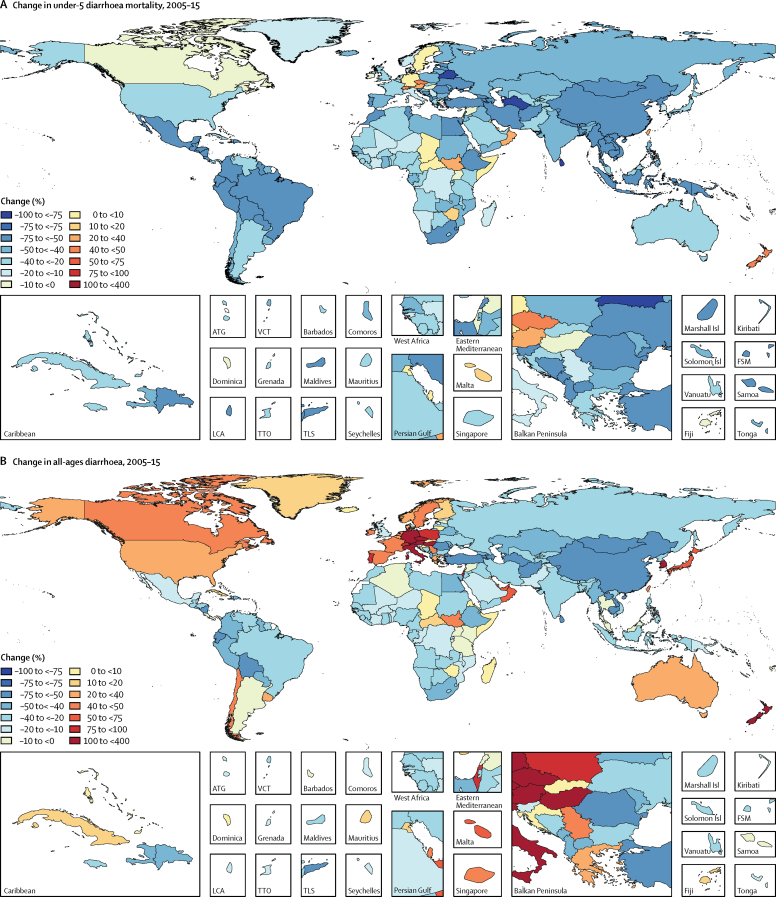
Change in diarrhoea deaths by geography, 2005–15 (A) Percentage change in under-5 deaths and (B) all-age deaths. ATG=Antigua and Barbuda. VCT=Saint Vincent and the Grenadines. LCA=Saint Lucia. TTO=Trinidad and Tobago. TLS=Timor-Leste. FSM=Federated States of Micronesia.

In 2015, diarrhoeal diseases were responsible for 1·31 million deaths (95% UI 1·23 million to 1·39 million; table 1). Among children younger than 5 years, diarrhoeal diseases were responsible for 499 000 deaths (447 000–558 000; table 1), representing 8·6% (7·7–9·5) of the 5·82 million deaths in this age group.^1^ Diarrhoea was the ninth leading cause of death among all ages and the fourth leading cause among children younger than 5 years, behind preterm birth complications, neonatal encephalopathy, and lower respiratory infections.^1^ At the global level, the diarrhoea mortality rate for children younger than 5 years in 2015 was 74·3 deaths (95% UI 66·6–83·0) per 100 000 and was slightly different between boys (74·1 deaths [64·4–85·1] per 100 000) and girls (74·5 deaths [65·0–85·5] per 100 000). Figure 1 shows mortality rates due to diarrhoea for children younger than 5 years by geography in 2015 and figure 2 shows all age mortality by geography. Under-5 mortality from diarrhoea was highest in sub-Saharan Africa and south Asia. Between 2005 and 2015, the number of deaths due to diarrhoea decreased by 34·3% (24·9–42·3) among children younger than 5 years and decreased by 20·8% among all ages (15·4–26·1). Figure 3 shows the rate of change in under-5 deaths due to diarrhoea between 2005 and 2015.

**Table 1:** Deaths, episodes, and DALYs attributable to diarrhoeal disease in 2015, by country

		**Children younger than 5 years**						**All ages**					
		Deaths			Episodes (100 000s)	DALYs		Deaths			Episodes (100 000s)	DALYs	
		Number	Mortality rate (per 100 000)	Percentage change, 2005–15		Number	Percentage change, 2005–15	Number	Mortality rate (per 100 000)	Percentage change, 2005–15		Number	Percentage change, 2005–15
**Global**		**498 888·8(447 450·0 to 557 643·5)**	**74·3(66·6 to 83·0)**	**−34·3%(−42·3 to −24·9)**	**9574·6(8711·4 to 10574·6)**	**45 109 455·2(40 694 368·7 to 50 119 268·8)**	**−33·1%(−40·9 to −24·0)**	**1 312 128·4(1233 574·1 to 1391 253·6)**	**17·8(16·7 to 18·9)**	**−20·8%(−26·1 to −15·4)**	**23 925·3(23 011·1 to 25 031)**	**71 589 510·5(66 442 883·8 to 77 205 834·9)**	**−27·2%(−33·2 to −20·8)**
**Central Europe, eastern Europe, and central Asia**		**1967·7 (1554·3 to 2465·7)**	**7·1 (5·6 to 8·9)**	**−49·6% (−59·9 to −36·7)**	**145·9 (130·1 to 162·9)**	**207 352·2 (169 890·4 to 250 705·8)**	**−44·6% (−54·5 to −32·4)**	**3179·8 (2772·1 to 3693·1)**	**0·8 (0·7 to 0·9)**	**−39·0% (−46·9 to −29·1)**	**313·7 (297 to 330·6)**	**274 787·8 (232 590·8 to 321 424·0)**	**−41·0% (−49·2 to −31·2)**
Albania		2·3 (1·3 to 3·8)	1·3 (0·7 to 2·1)	−64·5% (−80·5 to −37·2)	0·5 (0·4 to 0·6)	334·7 (232·5 to 470·0)	−56·2% (−69·5 to −36·0)	5·7 (4·4 to 7·3)	0·2 (0·2 to 0·3)	−46·3% (−60·5 to −28·6)	1·1 (1 to 1·1)	531·9 (412·4 to 693·3)	−50·2% (−61·3 to −35·6)
Armenia		10·1 (7·0 to 14·4)	5·1 (3·6 to 7·3)	−72·5% (−82·1 to −58·5)	1·8 (1·6 to 2·1)	1342·9 (1020·9 to 1766·4)	−64·7% (−74·3 to −51·9)	18·7 (15·2 to 23·2)	0·6 (0·5 to 0·8)	−64·0% (−72·2 to −52·9)	4·5 (4·2 to 4·8)	2156·9 (1716·1 to 2727·9)	−57·8% (−66·0 to −47·3)
Azerbaijan		176·8 (107·6 to 263·3)	18·4 (11·2 to 27·5)	−62·5% (−77·8 to −41·4)	9·2 (7·7 to 10·5)	17 641·7 (11 717·7 to 24 958·5)	−59·0% (−73·5 to −38·6)	201·3 (132·0 to 288·9)	2·1 (1·3 to 3·0)	−61·3% (−75·3 to −42·1)	16·9 (15·5 to 18·3)	20 399·5 (14 314·7 to 27 933·0)	−57·4% (−70·9 to −39·3)
Belarus		1·5 (0·9 to 2·4)	0·3 (0·2 to 0·4)	−77·7% (−86·5 to −63·8)	1·7 (1·5 to 1·9)	579·7 (408·6 to 778·5)	−46·9% (−59·1 to −34·5)	15·5 (13·0 to 19·4)	0·2 (0·1 to 0·2)	−40·4% (−51·6 to −25·5)	5 (4·8 to 5·1)	1516·4 (1159·6 to 1947·3)	−39·1% (−46·3 to −31·9)
Bosnia and Herzegovina		2·0 (1·1 to 3·5)	1·2 (0·6 to 2·0)	−59·0% (−81·3 to −12·4)	0·5 (0·4 to 0·6)	315·3 (214·7 to 456·6)	−46·7% (−68·0 to −13·0)	5·8 (4·5 to 7·4)	0·2 (0·1 to 0·2)	−32·2% (−54·7 to −1·9)	1·3 (1·2 to 1·4)	530·5 (397·8 to 691·5)	−38·1% (−56·4 to −14·7)
Bulgaria		7·9 (5·1 to 12·0)	2·3 (1·5 to 3·5)	−43·4% (−64·3 to −13·7)	0·9 (0·8 to 1)	916·2 (651·9 to 1278·3)	−37·2% (−55·2 to −12·8)	24·8 (20·2 to 33·0)	0·3 (0·3 to 0·5)	−22·0% (−36·3 to −5·4)	2·3 (2·2 to 2·5)	1547·9 (1230·2 to 1956·2)	−30·7% (−44·0 to −14·4)
Croatia		1·0 (0·7 to 1·3)	0·5 (0·4 to 0·6)	−31·8% (−50·0 to −8·6)	0·3 (0·3 to 0·3)	166·8 (129·7 to 214·7)	−19·7% (−34·1 to −4·8)	17·3 (12·2 to 21·0)	0·4 (0·3 to 0·5)	8·4% (−17·5 to 31·7)	1 (0·9 to 1)	558·1 (447·5 to 666·3)	−10·8% (−23·4 to −1·7)
Czech Republic		2·0 (1·5 to 2·8)	0·4 (0·3 to 0·5)	48·5% (7·1 to 107·2)	0·8 (0·7 to 0·9)	383·4 (290·7 to 483·3)	41·5% (18·7 to 70·2)	116·6 (30·9 to 147·6)	1·1 (0·3 to 1·4)	299·8% (−0·1 to 423·4)	2·5 (2·4 to 2·6)	2175·3 (1236·1 to 2578·0)	108·6% (6·5 to 148·8)
Estonia		0·2 (0·1 to 0·3)	0·3 (0·2 to 0·4)	−55·1% (−69·7 to −34·8)	0·2 (0·2 to 0·2)	72·3 (52·4 to 96·5)	−25·6% (−36·2 to −15·3)	1·8 (1·4 to 2·3)	0·1 (0·1 to 0·2)	−7·6% (−33·0 to 15·3)	0·6 (0·6 to 0·7)	180·9 (136·0 to 235·6)	−21·4% (−28·3 to −14·5)
Georgia		12·4 (8·7 to 16·8)	4·5 (3·1 to 6·0)	−54·8% (−69·8 to −31·3)	2·1 (1·8 to 2·4)	1622·6 (1247·7 to 2050·7)	−46·0% (−59·7 to −26·7)	23·7 (19·4 to 28·6)	0·6 (0·5 to 0·7)	−43·8% (−56·0 to −26·8)	5·1 (4·8 to 5·4)	2482·5 (1982·1 to 3038·8)	−41·1% (−52·3 to −27·4)
Hungary		3·9 (2·5 to 5·5)	0·8 (0·5 to 1·2)	−4·6% (−36·5 to 34·1)	0·7 (0·6 to 0·8)	520·3 (379·1 to 678·7)	−1·3% (−23·1 to 24·7)	111·8 (25·6 to 140·4)	1·1 (0·3 to 1·4)	233·6% (−6·9 to 313·8)	2·2 (2·1 to 2·3)	2280·8 (1104·6 to 2682·7)	70·2% (−6·7 to 97·3)
Kazakhstan		93·5 (67·6 to 127·9)	5·0 (3·6 to 6·9)	−48·7% (−65·4 to −22·7)	11·8 (10·6 to 13·2)	11 127·3 (8490·7 to 14 096·6)	−39·2% (−55·5 to −16·6)	121·8 (93·3 to 167·2)	0·7 (0·5 to 1·0)	−47·3% (−61·4 to −28·9)	23 (21·6 to 24·3)	14 735·0 (11 471·3 to 18 407·0)	−35·9% (−50·4 to −18·2)
Kyrgyzstan		175·9 (131·6 to 228·7)	24·0 (17·9 to 31·1)	−35·7% (−53·4 to −13·5)	7·4 (6·7 to 8·3)	17 051·8 (13 159·6 to 21 840·2)	−32·5% (−49·5 to −11·5)	196·9 (152·5 to 250·3)	3·3 (2·6 to 4·2)	−34·8% (−51·2 to −14·7)	12·3 (11·5 to 13·3)	19 135·2 (15 218·9 to 24 121·9)	−31·3% (−46·8 to −12·2)
Latvia		0·4 (0·2 to 0·5)	0·3 (0·2 to 0·5)	−65·4% (−78·2 to −45·8)	0·3 (0·2 to 0·3)	97·8 (71·6 to 128·1)	−40·3% (−53·1 to −27·8)	3·5 (2·7 to 4·3)	0·2 (0·1 to 0·2)	−20·9% (−37·6 to −4·7)	0·8 (0·8 to 0·9)	269·5 (207·3 to 342·9)	−29·7% (−37·3 to −22·5)
Lithuania		0·6 (0·4 to 0·9)	0·4 (0·3 to 0·6)	−42·3% (−60·3 to −19·1)	0·4 (0·4 to 0·4)	159·9 (119·3 to 207·2)	−23·8% (−35·3 to −10·9)	6·9 (4·9 to 8·1)	0·2 (0·2 to 0·3)	4·9% (−28·9 to 25·0)	1·3 (1·3 to 1·4)	472·7 (374·6 to 589·0)	−17·6% (−29·1 to −10·0)
Macedonia		4·6 (2·7 to 7·2)	4·0 (2·4 to 6·2)	−57·0% (−74·4 to −30·0)	0·4 (0·4 to 0·5)	510·4 (341·9 to 742·9)	−52·4% (−68·6 to −29·4)	7·6 (5·7 to 10·3)	0·4 (0·3 to 0·5)	−48·3% (−62·5 to −28·0)	0·9 (0·9 to 1)	684·7 (503·8 to 925·5)	−47·3% (−61·7 to −28·0)
Moldova		6·1 (3·7 to 9·3)	2·8 (1·7 to 4·3)	−49·6% (−71·3 to −20·3)	1·2 (1·1 to 1·4)	856·3 (604·1 to 1168·6)	−42·1% (−59·7 to −21·4)	9·8 (7·2 to 13·5)	0·2 (0·2 to 0·3)	−42·0% (−59·6 to −20·1)	2·8 (2·6 to 2·9)	1269·7 (961·1 to 1668·4)	−38·9% (−52·3 to −23·9)
Mongolia		2·1 (1·2 to 3·8)	0·6 (0·4 to 1·1)	−62·7% (−81·6 to −28·2)	2·5 (2·2 to 2·8)	879·7 (620·7 to 1196·1)	−17·1% (−39·7 to 5·6)	3·9 (2·8 to 5·6)	0·1 (0·1 to 0·2)	−52·2% (−68·9 to −28·1)	3·9 (3·6 to 4·2)	1234·0 (886·3 to 1665·2)	−20·3% (−36·9 to −5·5)
Montenegro		0·2 (0·1 to 0·3)	0·5 (0·2 to 0·9)	−72·4% (−87·3 to −40·5)	0·1 (0·1 to 0·1)	34·1 (23·0 to 49·2)	−57·2% (−72·6 to −34·5)	0·6 (0·4 to 0·7)	0·1 (0·1 to 0·1)	−46·5% (−63·1 to −23·9)	0·2 (0·2 to 0·2)	60·4 (45·2 to 79·7)	−45·4% (−59·2 to −28·0)
Poland		6·8 (4·6 to 9·4)	0·3 (0·2 to 0·5)	−40·5% (−59·8 to −14·3)	2·9 (2·6 to 3·2)	1346·1 (1010·0 to 1773·2)	−21·1% (−35·9 to −3·0)	120·5 (74·6 to 147·4)	0·3 (0·2 to 0·4)	80·3% (−8·9 to 122·9)	8·3 (8 to 8·6)	4095·1 (3245·4 to 4969·4)	3·8% (−14·8 to 15·8)
Romania		29·0 (19·7 to 42·9)	3·1 (2·1 to 4·6)	−71·7% (−81·2 to −57·6)	2·1 (1·9 to 2·4)	3057·5 (2209·1 to 4263·3)	−68·6% (−77·5 to −55·8)	57·5 (44·8 to 73·8)	0·3 (0·2 to 0·4)	−57·2% (−67·2 to −45·3)	5·5 (5·3 to 5·8)	4303·3 (3327·5 to 5539·8)	−62·6% (−71·0 to −51·3)
Russia		154·6 (124·2 to 189·3)	1·7 (1·4 to 2·1)	−49·5% (−60·5 to −35·3)	37·8 (33·5 to 42·5)	23 064·9 (18 536·9 to 28 323·0)	−36·5% (−47·0 to −24·9)	477·5 (418·7 to 612·3)	0·3 (0·3 to 0·4)	−38·3% (−46·9 to −29·8)	92·7 (87·9 to 97·8)	42 653·8 (35 136·1 to 51 777·1)	−35·2% (−43·1 to −27·9)
Serbia		4·0 (2·8 to 5·4)	0·9 (0·6 to 1·2)	−10·7% (−39·3 to 26·5)	0·9 (0·8 to 1)	573·6 (434·8 to 720·0)	−14·4% (−32·3 to 7·1)	33·0 (27·5 to 40·3)	0·4 (0·3 to 0·5)	45·5% (17·8 to 81·5)	2·4 (2·2 to 2·5)	1334·2 (1114·6 to 1579·4)	−2·3% (−13·3 to 12·1)
Slovakia		2·8 (2·0 to 3·9)	1·0 (0·7 to 1·4)	−30·3% (−53·1 to 2·4)	0·5 (0·4 to 0·5)	363·2 (285·1 to 473·5)	−20·5% (−40·3 to 4·0)	20·3 (16·8 to 24·3)	0·4 (0·3 to 0·4)	6·3% (−13·1 to 28·9)	1·2 (1·2 to 1·3)	829·3 (703·7 to 975·5)	−12·9% (−24·2 to 0·1)
Slovenia		0·2 (0·1 to 0·3)	0·2 (0·1 to 0·3)	−54·4% (−67·3 to −36·9)	0·1 (0·1 to 0·2)	56·7 (42·3 to 74·9)	−24·5% (−37·2 to −8·1)	4·3 (2·5 to 5·5)	0·2 (0·1 to 0·3)	−11·7% (−30·7 to 16·0)	0·4 (0·4 to 0·4)	157·2 (123·3 to 198·3)	−21·0% (−29·5 to −10·7)
Tajikistan		878·3 (534·3 to 1313·2)	73·5 (44·7 to 109·9)	−36·7% (−62·3 to −1·2)	17·8 (15·7 to 20·8)	80 115·2 (50 418·6 to 116 601·2)	−35·5% (−59·9 to −1·3)	986·8 (644·9 to 1417·4)	11·6 (7·6 to 16·7)	−35·9% (−59·0 to −4·3)	28·4 (26·2 to 31·4)	87 872·3 (58 394·6 to 124 557·7)	−35·2% (−57·9 to −4·2)
Turkmenistan		162·7 (99·1 to 246·5)	30·4 (18·5 to 46·0)	−75·4% (−85·1 to −59·6)	6 (5·2 to 6·9)	15 562·9 (9901·7 to 22 840·9)	−73·6% (−83·4 to −58·3)	199·5 (135·4 to 284·0)	3·7 (2·5 to 5·3)	−73·1% (−82·2 to −59·0)	11·3 (10·5 to 12·3)	18 554·4 (12 765·2 to 26 230·1)	−71·3% (−80·7 to −57·1)
Ukraine		27·9 (16·1 to 44·7)	1·2 (0·7 to 1·8)	−49·7% (−72·9 to −13·4)	9·5 (8·3 to 10·6)	4877·3 (3510·8 to 6560·2)	−32·9% (−51·4 to −9·1)	99·2 (76·0 to 172·4)	0·2 (0·2 to 0·4)	−29·1% (−43·8 to −10·4)	26·6 (25·3 to 27·9)	9719·0 (7363·3 to 12 946·4)	−30·9% (−42·1 to −18·6)
Uzbekistan		197·6 (123·8 to 299·2)	6·1 (3·8 to 9·2)	−35·3% (−62·7 to 5·4)	25·6 (21·5 to 29·8)	23 721·8 (17 192·1 to 31 900·4)	−27·1% (−51·7 to 7·0)	287·1 (212·9 to 386·3)	1·0 (0·7 to 1·3)	−30·8% (−53·0 to −0·0)	49·2 (45 to 53·5)	33 047·4 (25 629·3 to 42 309·0)	−24·9% (−44·5 to −0·4)
**High-income**		**683·7 (630·2 to 740·8)**	**1·2 (1·1 to 1·3)**	**−30·5% (−36·4 to −24·4)**	**26·5 (24·1 to 29·3)**	**66 050·7 (60 451·9 to 71 860·4)**	**−28·4% (−34·1 to −22·6)**	**25 701·2 (15 008·1 to 28 112·6)**	**2·4 (1·4 to 2·6)**	**47·7% (16·4 to 57·5)**	**111·3 (108·5 to 114·2)**	**342 122·9 (234 939·7 to 368 698·4)**	**15·4% (−6·6 to 21·0)**
	Andorra	0·0 (0·0 to 0·0)	0·2 (0·1 to 0·4)	−45·0% (−71·8 to 8·2)	0 (0 to 0)	1·0 (0·7 to 1·6)	−32·9% (−57·2 to 5·9)	0·7 (0·5 to 1·0)	0·9 (0·6 to 1·2)	60·6% (13·1 to 135·6)	0 (0 to 0)	7·8 (6·5 to 9·5)	21·3% (−0·2 to 50·5)
	Argentina	140·8 (113·1 to 171·7)	3·8 (3·0 to 4·6)	−33·7% (−47·0 to −16·9)	5·1 (4·4 to 6)	13 504·4 (11 055·9 to 16 354·3)	−30·9% (−43·5 to −14·7)	658·4 (525·0 to 790·9)	1·5 (1·2 to 1·8)	−2·8% (−20·4 to 17·9)	13·7 (13 to 14·6)	22 862·4 (19 533·9 to 26 243·9)	−21·0% (−31·3 to −9·4)
	Australia	11·5 (9·0 to 14·6)	0·7 (0·6 to 0·9)	−23·3% (−43·5 to 2·8)	0·6 (0·5 to 0·7)	1147·5 (913·9 to 1419·3)	−18·4% (−37·9 to 4·5)	156·7 (106·1 to 197·1)	0·6 (0·4 to 0·8)	33·2% (6·3 to 67·5)	1·9 (1·8 to 2)	3023·6 (2396·5 to 3451·3)	3·9% (−9·3 to 17·7)
	Austria	4·3 (3·4 to 5·5)	1·1 (0·8 to 1·4)	34·9% (2·7 to 78·1)	0·2 (0·2 to 0·3)	430·5 (349·4 to 531·2)	32·4% (4·8 to 67·8)	73·0 (33·5 to 92·3)	0·8 (0·4 to 1·1)	119·4% (18·2 to 185·2)	1 (1 to 1·1)	1349·9 (964·4 to 1534·6)	61·1% (29·9 to 83·7)
	Belgium	6·2 (4·9 to 7·7)	1·0 (0·8 to 1·2)	−23·4% (−40·1 to −0·7)	0·3 (0·3 to 0·4)	621·3 (511·8 to 755·1)	−20·7% (−36·0 to −0·2)	434·0 (200·8 to 561·3)	3·8 (1·8 to 5·0)	23·5% (−4·6 to 65·0)	1·2 (1·2 to 1·3)	4787·4 (2780·6 to 5622·9)	6·4% (−9·4 to 25·2)
	Brunei	0·5 (0·4 to 0·6)	1·4 (1·0 to 1·9)	−2·8% (−31·7 to 36·8)	0 (0 to 0)	44·2 (33·4 to 58·7)	−2·7% (−30·4 to 33·1)	1·1 (1·0 to 1·4)	0·3 (0·2 to 0·3)	17·5% (−6·0 to 44·0)	0 (0 to 0)	63·6 (52·1 to 78·5)	1·8% (−19·8 to 28·5)
	Canada	14·4 (11·6 to 18·3)	0·8 (0·6 to 1·0)	−2·4% (−24·2 to 25·4)	0·3 (0·2 to 0·3)	1313·1 (1062·0 to 1646·0)	−1·0% (−22·1 to 25·9)	1198·8 (273·4 to 1563·8)	3·3 (0·8 to 4·3)	45·1% (12·3 to 82·6)	1·3 (1·2 to 1·3)	13 171·1 (4668·2 to 15 895·2)	27·3% (7·3 to 47·7)
	Chile	10·3 (8·3 to 12·7)	0·9 (0·7 to 1·1)	−46·7% (−58·8 to −32·8)	0·7 (0·7 to 0·8)	1098·7 (905·3 to 1319·3)	−42·0% (−53·2 to −29·6)	374·6 (278·4 to 475·1)	2·1 (1·6 to 2·6)	47·9% (13·6 to 93·2)	2·9 (2·8 to 3)	5583·6 (4456·1 to 6420·3)	4·2% (−10·8 to 20·4)
	Cyprus	0·9 (0·7 to 1·2)	2·4 (1·8 to 3·2)	−35·6% (−55·0 to −9·1)	0 (0 to 0)	80·9 (60·5 to 105·3)	−35·0% (−53·9 to −9·9)	10·6 (8·3 to 13·6)	1·2 (0·9 to 1·5)	12·6% (−14·7 to 49·8)	0·1 (0·1 to 0·1)	211·6 (184·5 to 244·2)	−14·5% (−28·3 to 1·9)
	Denmark	3·9 (3·0 to 4·9)	1·3 (1·0 to 1·7)	−17·2% (−36·0 to 5·8)	0·2 (0·1 to 0·2)	378·1 (296·8 to 468·5)	−15·7% (−32·3 to 5·7)	240·4 (102·5 to 310·6)	4·2 (1·8 to 5·4)	30·5% (−3·2 to 69·7)	0·6 (0·5 to 0·6)	2719·9 (1540·7 to 3237·7)	18·5% (−5·5 to 40·9)
	Finland	0·6 (0·4 to 0·8)	0·2 (0·1 to 0·3)	−43·6% (−62·2 to −17·7)	0·1 (0·1 to 0·1)	81·7 (60·2 to 108·1)	−31·3% (−46·4 to −11·3)	61·5 (45·5 to 79·9)	1·1 (0·8 to 1·4)	12·4% (−12·6 to 45·8)	0·6 (0·5 to 0·6)	758·7 (620·9 to 910·1)	−4·9% (−17·7 to 9·6)
	France	36·9 (25·9 to 50·1)	0·9 (0·7 to 1·3)	−36·7% (−55·9 to −12·5)	2·7 (2·4 to 3)	3909·5 (2916·5 to 5014·2)	−32·1% (−49·6 to −11·6)	2391·6 (1461·8 to 3121·4)	3·7 (2·2 to 4·8)	46·4% (11·3 to 92·4)	10·6 (10·2 to 10·9)	26 395·6 (18 514·0 to 31 145·8)	13·3% (−6·2 to 32·0)
	Germany	17·3 (13·3 to 22·2)	0·5 (0·4 to 0·7)	5·1% (−23·0 to 38·4)	1·8 (1·6 to 2)	1983·4 (1591·5 to 2429·0)	4·3% (−17·6 to 30·1)	3829·3 (1215·1 to 4922·9)	4·6 (1·5 to 5·9)	167·6% (17·2 to 245·3)	11·3 (10·9 to 11·6)	40 695·1 (16 036·7 to 48 848·5)	110·9% (8·4 to 149·9)
	Greece	0·4 (0·3 to 0·6)	0·1 (0·1 to 0·1)	−38·9% (−53·0 to −21·1)	0·1 (0·1 to 0·1)	64·6 (49·0 to 85·7)	−25·9% (−40·6 to −8·4)	17·5 (13·5 to 25·9)	0·2 (0·1 to 0·2)	40·0% (11·8 to 80·0)	0·7 (0·7 to 0·7)	369·1 (304·4 to 472·8)	3·7% (−5·9 to 15·1)
	Greenland	0·1 (0·1 to 0·2)	1·8 (1·1 to 2·9)	−11·2% (−52·4 to 62·8)	0 (0 to 0)	12·6 (7·4 to 20·0)	−11·0% (−51·9 to 61·1)	1·0 (0·8 to 1·2)	1·8 (1·6 to 2·2)	17·0% (−4·8 to 43·0)	0 (0 to 0)	32·2 (26·0 to 40·2)	2·2% (−23·0 to 33·7)
	Iceland	0·1 (0·1 to 0·1)	0·4 (0·3 to 0·6)	−43·1% (−59·2 to −23·0)	0 (0 to 0)	10·0 (7·6 to 13·0)	−37·2% (−52·7 to −19·2)	1·9 (1·2 to 2·5)	0·6 (0·4 to 0·8)	9·7% (−14·3 to 42·1)	0 (0 to 0)	32·7 (24·3 to 37·8)	−12·8% (−24·8 to −1·0)
	Ireland	2·6 (2·0 to 3·3)	0·7 (0·6 to 0·9)	−4·4% (−28·9 to 27·7)	0·2 (0·1 to 0·2)	265·5 (213·4 to 331·7)	−1·1% (−22·8 to 28·4)	25·0 (17·6 to 31·1)	0·5 (0·4 to 0·7)	43·0% (6·5 to 83·5)	0·4 (0·4 to 0·4)	598·5 (475·7 to 686·2)	15·5% (−0·2 to 34·1)
	Israel	8·5 (6·7 to 10·8)	1·0 (0·8 to 1·3)	−3·5% (−25·3 to 24·1)	0·4 (0·3 to 0·4)	833·9 (673·7 to 1026·7)	−1·6% (−21·4 to 24·1)	220·4 (100·5 to 277·4)	2·7 (1·2 to 3·4)	76·3% (22·8 to 120·9)	0·8 (0·8 to 0·9)	3062·5 (2006·9 to 3542·8)	34·3% (8·8 to 53·5)
	Italy	10·5 (7·7 to 14·2)	0·4 (0·3 to 0·5)	−15·7% (−39·6 to 16·5)	1 (0·9 to 1·1)	1173·4 (907·5 to 1511·7)	−11·2% (−31·6 to 15·7)	558·7 (383·4 to 716·0)	0·9 (0·6 to 1·1)	110·8% (22·4 to 178·3)	5 (4·9 to 5·2)	7064·7 (5168·3 to 8311·7)	48·7% (7·5 to 72·8)
	Japan	36·9 (31·3 to 41·8)	0·7 (0·6 to 0·8)	−27·0% (−35·2 to −16·9)	1·7 (1·6 to 2)	3636·5 (3140·5 to 4076·9)	−25·2% (−32·7 to −16·4)	3108·2 (2392·5 to 3524·9)	2·4 (1·9 to 2·7)	51·3% (39·4 to 63·7)	11 (10·7 to 11·3)	33 297·6 (25 074·1 to 37 750·0)	17·3% (9·8 to 24·9)
	Luxembourg	0·6 (0·5 to 0·8)	2·0 (1·6 to 2·6)	14·4% (−14·8 to 53·5)	0 (0 to 0)	57·4 (45·0 to 72·7)	13·9% (−13·0 to 49·9)	10·7 (6·7 to 13·7)	1·9 (1·2 to 2·5)	50·0% (12·9 to 94·6)	0·1 (0 to 0·1)	171·4 (124·4 to 197·7)	26·5% (8·0 to 47·3)
	Malta	0·4 (0·3 to 0·5)	2·2 (1·7 to 2·8)	16·4% (−11·7 to 55·9)	0 (0 to 0)	36·6 (29·1 to 45·8)	15·6% (−10·8 to 52·0)	4·5 (2·9 to 5·5)	1·1 (0·7 to 1·3)	59·9% (21·1 to 96·7)	0 (0 to 0)	93·7 (72·4 to 107·4)	31·5% (11·8 to 52·6)
	Netherlands	6·6 (5·1 to 8·2)	0·7 (0·6 to 0·9)	−15·5% (−35·3 to 8·8)	0·8 (0·7 to 0·8)	780·5 (629·8 to 939·6)	−13·4% (−29·0 to 6·4)	293·1 (155·7 to 375·6)	1·7 (0·9 to 2·2)	45·4% (6·0 to 88·3)	3·2 (3·1 to 3·3)	4185·0 (2759·9 to 4920·6)	22·0% (−0·4 to 39·3)
	New Zealand	3·7 (3·0 to 4·7)	1·2 (1·0 to 1·5)	45·3% (13·2 to 86·6)	0·1 (0·1 to 0·1)	351·1 (286·9 to 431·8)	42·1% (13·6 to 78·6)	55·6 (29·9 to 71·3)	1·2 (0·7 to 1·6)	121·1% (22·7 to 194·1)	0·3 (0·3 to 0·3)	887·5 (662·4 to 1030·2)	67·3% (30·4 to 97·3)
	Norway	2·9 (2·1 to 3·7)	1·0 (0·7 to 1·2)	−11·5% (−31·2 to 14·2)	0·1 (0·1 to 0·2)	280·5 (216·0 to 351·0)	−10·1% (−28·6 to 12·7)	237·7 (75·8 to 311·7)	4·6 (1·5 to 6·0)	29·4% (−2·0 to 69·3)	0·4 (0·4 to 0·4)	2304·2 (1054·3 to 2794·8)	16·1% (−6·5 to 38·0)
	Portugal	4·1 (3·3 to 5·0)	0·9 (0·7 to 1·1)	−57·3% (−66·5 to −45·9)	0·2 (0·2 to 0·2)	409·2 (337·1 to 490·3)	−54·5% (−63·4 to −43·8)	116·5 (78·7 to 148·1)	1·1 (0·7 to 1·4)	77·6% (−5·6 to 129·4)	0·9 (0·9 to 1)	1823·1 (1370·2 to 2081·6)	1·9% (−29·4 to 19·4)
	Singapore	0·8 (0·6 to 1·0)	0·4 (0·3 to 0·5)	−26·1% (−44·2 to −3·0)	0 (0 to 0·1)	80·5 (59·8 to 100·5)	−23·7% (−40·7 to −2·3)	34·3 (26·3 to 56·2)	0·9 (0·7 to 1·4)	47·9% (19·8 to 85·5)	0·3 (0·3 to 0·3)	534·4 (455·0 to 801·4)	9·3% (−1·9 to 23·3)
	South Korea	8·5 (6·4 to 11·2)	0·4 (0·3 to 0·5)	−12·0% (−35·2 to 19·2)	0·6 (0·5 to 0·6)	882·8 (688·5 to 1133·2)	−10·9% (−31·4 to 16·2)	796·4 (623·5 to 1048·5)	1·6 (1·2 to 2·1)	200·6% (55·1 to 304·7)	3·5 (3·4 to 3·6)	10 613·7 (8921·2 to 14 114·7)	86·8% (17·6 to 126·2)
	Spain	9·0 (7·1 to 11·2)	0·4 (0·3 to 0·5)	−45·8% (−58·5 to −29·5)	0·7 (0·6 to 0·9)	974·9 (787·1 to 1180·5)	−40·1% (−52·7 to −25·8)	769·3 (530·8 to 985·7)	1·6 (1·1 to 2·0)	41·2% (5·7 to 84·6)	3·4 (3·3 to 3·6)	8576·2 (6251·2 to 10 038·4)	9·5% (−11·6 to 27·4)
	Sweden	2·6 (1·6 to 3·2)	0·4 (0·3 to 0·6)	4·5% (−17·0 to 32·7)	0·2 (0·2 to 0·2)	273·4 (185·0 to 334·6)	8·6% (−10·3 to 32·2)	303·9 (129·4 to 390·1)	3·1 (1·3 to 4·0)	44·7% (−0·6 to 83·4)	0·7 (0·6 to 0·7)	2903·6 (1570·8 to 3458·7)	30·3% (−1·9 to 52·3)
	Switzerland	4·7 (3·7 to 6·0)	1·1 (0·9 to 1·5)	48·9% (13·9 to 93·7)	0·2 (0·2 to 0·2)	461·4 (366·7 to 572·1)	45·9% (14·9 to 85·3)	135·2 (89·9 to 170·7)	1·6 (1·1 to 2·1)	82·5% (14·0 to 141·3)	0·8 (0·7 to 0·8)	1787·2 (1391·4 to 2055·2)	54·9% (17·6 to 81·4)
	UK	30·2 (26·1 to 33·6)	0·8 (0·6 to 0·8)	−28·1% (−36·0 to −19·4)	6·5 (6·3 to 6·7)	4332·5 (3701·5 to 5036·0)	−24·5% (−29·9 to −18·7)	1484·4 (767·5 to 1664·7)	2·3 (1·2 to 2·6)	−18·5% (−25·5 to −0·1)	25·9 (25·7 to 26·2)	22 069·6 (14 883·3 to 24 989·7)	−18·6% (−22·8 to −10·4)
	USA	292·5 (257·7 to 331·9)	1·5 (1·3 to 1·7)	−33·1% (−41·5 to −23·1)	1·4 (1·2 to 1·6)	25 566·8 (22 534·3 to 28 966·8)	−32·8% (−41·3 to −23·0)	7945·2 (2804·6 to 8799·2)	2·5 (0·9 to 2·7)	36·8% (3·7 to 46·3)	8·1 (7·8 to 8·3)	117 521·7 (61 201·8 to 127 694·9)	11·1% (−13·0 to 18·2)
	Uruguay	10·5 (7·4 to 14·7)	4·3 (3·0 to 6·0)	−40·7% (−60·4 to −13·2)	0·2 (0·1 to 0·2)	945·4 (674·6 to 1311·9)	−39·9% (−58·6 to −12·9)	139·1 (103·4 to 177·8)	4·0 (3·0 to 5·2)	22·0% (−16·3 to 59·8)	0·5 (0·5 to 0·5)	2400·5 (1947·1 to 2840·0)	−15·8% (−32·6 to 2·2)
**Latin America and Caribbean**		**9367·4 (8139·6 to 10 924·1)**	**19·3 (16·7 to 22·5)**	**−58·8% (−64·4 to −51·8)**	**668·8 (604·7 to 742·3)**	**980 659·6 (857 961·4 to 1138 212·7)**	**−54·6% (−60·0 to −47·8)**	**24 053·1 (22 336·8 to 28 008·3)**	**4·2 (3·9 to 4·9)**	**−35·4% (−39·8 to −30·1)**	**1720·3 (1654·3 to 1795·1)**	**1542 673·4 (1368 480·1 to 1753 664·8)**	**−44·0% (−49·1 to −38·5)**
Antigua and Barbuda		0·6 (0·4 to 0·9)	8·5 (5·6 to 11·7)	−28·6% (−48·5 to −1·8)	0·1 (0·1 to 0·1)	77·3 (57·6 to 99·8)	−20·9% (−37·4 to 0·1)	2·1 (1·7 to 2·6)	2·2 (1·8 to 2·8)	−13·4% (−27·9 to 3·6)	0·3 (0·3 to 0·3)	142·4 (115·2 to 176·7)	−10·9% (−22·2 to 2·5)
Barbados		0·6 (0·3 to 1·0)	3·6 (2·1 to 6·1)	−31·2% (−63·5 to 29·6)	0·2 (0·2 to 0·2)	101·3 (71·6 to 145·8)	−15·5% (−41·7 to 22·6)	3·9 (3·0 to 5·6)	1·4 (1·1 to 2·0)	−3·8% (−23·4 to 23·8)	0·7 (0·7 to 0·7)	258·9 (199·2 to 329·7)	−0·2% (−16·0 to 19·5)
Belize		4·9 (3·1 to 8·3)	12·7 (7·8 to 21·4)	−24·8% (−57·1 to 28·4)	0·5 (0·4 to 0·6)	558·6 (387·8 to 819·1)	−17·9% (−46·4 to 25·1)	9·8 (7·6 to 13·1)	2·7 (2·1 to 3·7)	−11·9% (−35·1 to 19·5)	1·2 (1·1 to 1·3)	862·7 (674·0 to 1133·0)	−9·5% (−32·0 to 20·6)
Bermuda		0·0 (0·0 to 0·0)	0·7 (0·4 to 1·1)	−44·9% (−68·7 to −6·9)	0 (0 to 0)	11·7 (8·4 to 15·5)	−8·3% (−23·1 to 6·5)	0·3 (0·2 to 0·5)	0·5 (0·4 to 0·8)	−3·5% (−21·1 to 16·4)	0·1 (0·1 to 0·1)	36·5 (26·5 to 48·0)	6·1% (−2·3 to 14·0)
Bolivia		470·0 (277·7 to 732·3)	38·3 (22·6 to 59·7)	−67·3% (−79·9 to −47·8)	31·7 (28·1 to 36·3)	48 411·8 (31 743·2 to 71 092·1)	−63·9% (−75·8 to −46·3)	710·0 (519·7 to 976·7)	6·6 (4·8 to 9·1)	−59·1% (−69·8 to −42·9)	58·8 (55·1 to 63·5)	61 025·2 (44 180·0 to 83 478·6)	−59·4% (−70·3 to −44·0)
Brazil		1760·7 (1530·5 to 2032·3)	11·7 (10·2 to 13·5)	−69·9% (−74·6 to −64·6)	229·5 (208·7 to 253·8)	211 500·2 (179 999·4 to 245 446·4)	−63·7% (−68·5 to −58·2)	6342·9 (5942·3 to 6732·0)	3·1 (2·9 to 3·2)	−37·2% (−42·0 to −31·5)	734·3 (712·8 to 759·8)	410 590·5 (349 758·9 to 478 186·3)	−47·6% (−52·8 to −42·0)
Colombia		335·5 (240·6 to 475·7)	8·9 (6·4 to 12·6)	−65·7% (−76·0 to −50·3)	54·5 (49·2 to 60·6)	43 691·5 (33 831·0 to 55 929·1)	−56·8% (−67·1 to −43·4)	913·1 (764·2 to 1341·5)	1·9 (1·6 to 2·8)	−43·2% (−52·9 to −29·4)	142·8 (136·9 to 149·1)	75 502·7 (60 625·1 to 94 245·6)	−42·9% (−52·7 to −32·0)
Costa Rica		16·3 (10·9 to 23·9)	4·6 (3·1 to 6·8)	−52·9% (−67·4 to −32·8)	4·2 (3·8 to 4·7)	2569·4 (1891·1 to 3405·6)	−37·9% (−51·1 to −21·8)	98·7 (81·3 to 119·9)	2·1 (1·7 to 2·5)	−4·4% (−20·1 to 14·5)	11·7 (11·2 to 12·1)	5404·2 (4274·1 to 6676·6)	−18·2% (−29·5 to −6·1)
Cuba		13·7 (10·9 to 17·2)	2·3 (1·8 to 2·9)	−37·7% (−51·9 to −17·8)	4·4 (4 to 4·8)	2355·1 (1872·6 to 2950·1)	−23·6% (−35·4 to −10·7)	312·8 (221·7 to 370·4)	2·7 (1·9 to 3·3)	11·5% (−7·5 to 32·4)	25·4 (24·6 to 26·2)	11 250·3 (9174·5 to 13 627·0)	−1·4% (−10·3 to 5·9)
Dominica		1·2 (0·7 to 2·1)	21·5 (12·7 to 37·0)	−6·9% (−43·9 to 51·5)	0·1 (0·1 to 0·1)	126·5 (83·1 to 200·5)	−3·8% (−35·8 to 45·4)	3·0 (2·4 to 4·0)	4·2 (3·3 to 5·6)	1·7% (−21·1 to 30·7)	0·3 (0·2 to 0·3)	201·1 (150·8 to 277·4)	−1·5% (−23·8 to 30·4)
Dominican Republic		212·9 (147·7 to 298·4)	20·0 (13·9 to 28·1)	−58·8% (−71·9 to −40·5)	17·3 (15·7 to 19·3)	22 952·0 (17 165·9 to 30 630·2)	−53·7% (−66·5 to −36·4)	413·0 (336·6 to 509·5)	3·9 (3·2 to 4·8)	−42·8% (−54·8 to −27·5)	45·3 (43·4 to 47·5)	33 932·4 (27 115·7 to 42 117·0)	−44·2% (−55·9 to −29·5)
Ecuador		227·8 (169·5 to 304·1)	14·1 (10·5 to 18·8)	−64·5% (−73·7 to −53·2)	28·4 (24·8 to 31·4)	26 887·0 (21 163·5 to 33 397·3)	−57·4% (−66·1 to −46·7)	484·8 (411·2 to 572·3)	3·0 (2·5 to 3·5)	−50·5% (−58·5 to −40·4)	60 (56·1 to 63)	39 815·1 (32 852·2 to 47 382·1)	−49·4% (−57·3 to −39·9)
El Salvador		49·6 (31·1 to 71·8)	9·4 (5·9 to 13·5)	−71·9% (−83·1 to −57·3)	5·1 (4·4 to 5·9)	5647·8 (3995·9 to 7658·9)	−66·9% (−77·4 to −53·8)	212·2 (179·8 to 250·3)	3·5 (2·9 to 4·1)	−43·1% (−52·7 to −29·9)	11·8 (11·1 to 12·6)	11 269·6 (9310·8 to 13 592·6)	−54·4% (−63·2 to −43·8)
Grenada		0·7 (0·4 to 1·2)	6·8 (3·7 to 12·1)	−20·0% (−58·6 to 53·7)	0·1 (0·1 to 0·2)	99·7 (67·8 to 146·5)	−7·5% (−37·9 to 42·4)	2·2 (1·8 to 2·8)	2·1 (1·7 to 2·6)	−14·9% (−34·1 to 11·6)	0·4 (0·4 to 0·4)	190·8 (146·3 to 244·3)	−5·3% (−23·3 to 19·7)
Guatemala		1300·2 (992·3 to 1702·9)	61·9 (47·2 to 81·0)	−52·8% (−62·9 to −40·5)	47·5 (41·1 to 54·3)	123 583·3 (96 780·6 to 158 637·4)	−50·2% (−59·9 to −38·7)	2911·2 (2339·4 to 4217·9)	17·8 (14·3 to 25·8)	−38·6% (−49·4 to −25·0)	84·1 (77·3 to 91)	182 140·2 (150 962·4 to 228 310·0)	−44·3% (−52·9 to −34·2)
Guyana		28·4 (19·3 to 42·0)	40·5 (27·5 to 59·9)	−55·4% (−68·6 to −35·5)	1·1 (1 to 1·3)	2743·7 (1922·7 to 3905·1)	−53·2% (−65·6 to −34·6)	56·9 (45·8 to 70·8)	7·4 (5·9 to 9·2)	−44·3% (−54·6 to −31·6)	2·8 (2·6 to 3)	4015·2 (3134·4 to 5188·4)	−47·0% (−57·6 to −32·8)
Haiti		2370·7 (1423·0 to 3765·7)	190·0 (114·0 to 301·8)	−47·9% (−68·9 to −14·4)	34·2 (29·9 to 40·2)	212 391·3 (131 143·0 to 333 031·8)	−46·8% (−67·2 to −14·1)	3132·2 (2131·0 to 4542·5)	29·2 (19·9 to 42·4)	−42·3% (−60·4 to −13·4)	66·2 (61·7 to 72·5)	245 795·2 (162 820·1 to 367 372·9)	−44·1% (−63·0 to −14·3)
Honduras		248·2 (170·2 to 349·3)	29·6 (20·3 to 41·7)	−55·3% (−69·8 to −30·4)	17·9 (15·3 to 20·9)	26 129·1 (18 999·6 to 34 505·9)	−51·0% (−65·1 to −27·8)	963·0 (776·0 to 1211·2)	11·9 (9·6 to 15·0)	−26·3% (−40·6 to −8·5)	35·3 (32·6 to 38·4)	47 976·7 (39 675·2 to 58 832·3)	−38·9% (−50·9 to −22·9)
Jamaica		16·4 (10·3 to 25·7)	6·8 (4·3 to 10·7)	−37·6% (−65·6 to 12·7)	2·7 (2·3 to 3·2)	2133·2 (1508·5 to 3034·5)	−27·9% (−53·5 to 10·9)	47·6 (37·8 to 60·2)	1·7 (1·3 to 2·1)	−20·3% (−40·2 to 8·4)	7·7 (7·3 to 8·2)	3835·2 (2968·5 to 4973·9)	−19·5% (−39·0 to 3·6)
Mexico		1094·1 (963·3 to 1246·7)	9·4 (8·3 to 10·7)	−58·7% (−63·9 to −52·7)	66·7 (59·4 to 75·4)	111 997·6 (99 444·6 to 126 391·3)	−54·3% (−59·6 to −48·4)	4508·2 (4039·8 to 6409·7)	3·5 (3·2 to 5·0)	−16·9% (−23·0 to −9·9)	152·5 (145·2 to 161·4)	199 870·8 (179 688·5 to 242 840·3)	−39·1% (−44·3 to −33·2)
Nicaragua		98·1 (64·3 to 142·3)	16·0 (10·5 to 23·2)	−70·3% (−80·7 to −54·9)	9·8 (8·4 to 11·3)	11 109·8 (7977·5 to 14 919·4)	−64·9% (−75·0 to −50·7)	180·5 (142·3 to 224·9)	3·0 (2·3 to 3·7)	−57·8% (−68·0 to −44·1)	19·4 (18·1 to 20·9)	15 379·3 (11 827·6 to 19 349·1)	−58·3% (−68·1 to −45·3)
Panama		86·8 (61·2 to 123·0)	23·6 (16·6 to 33·4)	−23·4% (−45·7 to 8·9)	6·2 (5·3 to 7·2)	9112·3 (6768·8 to 12 294·6)	−18·5% (−39·0 to 9·1)	204·4 (170·4 to 245·1)	5·2 (4·3 to 6·2)	−0·5% (−19·2 to 22·5)	13·6 (12·7 to 14·5)	13 871·3 (11 295·5 to 17 138·4)	−9·4% (−26·0 to 10·2)
Paraguay		90·7 (59·0 to 136·8)	13·2 (8·6 to 19·9)	−63·3% (−77·1 to −41·5)	9·8 (8·6 to 11·1)	10 363·7 (7384·4 to 14 288·0)	−56·5% (−69·8 to −36·6)	224·5 (185·4 to 274·3)	3·4 (2·8 to 4·1)	−44·7% (−56·7 to −28·6)	23·1 (21·9 to 24·5)	16 536·8 (12 896·7 to 20 818·6)	−46·8% (−58·8 to −30·6)
Peru		230·1 (174·0 to 305·2)	7·6 (5·7 to 10·1)	−60·3% (−70·7 to −45·7)	52·3 (47·1 to 58·1)	33 268·2 (26 449·1 to 41 541·3)	−49·6% (−59·5 to −37·8)	626·9 (530·6 to 738·5)	2·0 (1·7 to 2·4)	−39·9% (−50·4 to −26·2)	116·5 (111 to 122·6)	56 184·3 (45 558·2 to 68 892·5)	−38·8% (−47·8 to −29·4)
Puerto Rico		4·1 (3·1 to 5·2)	1·8 (1·4 to 2·4)	−44·6% (−58·1 to −26·6)	1·8 (1·7 to 1·9)	859·0 (670·8 to 1077·1)	−23·8% (−35·0 to −11·6)	74·1 (43·0 to 92·6)	2·0 (1·2 to 2·5)	2·2% (−17·2 to 26·6)	9·2 (9 to 9·4)	3276·3 (2532·9 to 4049·0)	−2·8% (−9·9 to 3·9)
Saint Lucia		1·1 (0·6 to 2·0)	7·8 (4·3 to 14·7)	−48·3% (−74·8 to 9·4)	0·2 (0·2 to 0·2)	140·4 (93·2 to 223·0)	−36·9% (−61·9 to 10·6)	4·6 (3·7 to 6·1)	2·5 (2·0 to 3·3)	−13·0% (−33·1 to 14·9)	0·5 (0·5 to 0·6)	279·1 (215·4 to 371·5)	−19·9% (−39·8 to 10·1)
Saint Vincent and the Grenadines		1·4 (0·8 to 2·4)	15·8 (9·6 to 26·8)	−41·8% (−64·1 to −1·9)	0·1 (0·1 to 0·1)	151·9 (103·0 to 236·6)	−36·7% (−56·7 to −2·6)	4·0 (3·2 to 5·2)	3·6 (2·9 to 4·7)	−24·2% (−39·0 to −4·0)	0·4 (0·3 to 0·4)	262·4 (203·8 to 354·0)	−26·7% (−42·4 to −3·0)
Suriname		12·0 (8·0 to 17·8)	25·2 (16·8 to 37·2)	−48·4% (−62·9 to −27·0)	0·6 (0·5 to 0·7)	1207·7 (861·7 to 1683·8)	−45·0% (−59·0 to −25·4)	23·7 (19·0 to 29·9)	4·4 (3·5 to 5·5)	−34·1% (−46·0 to −19·0)	1·7 (1·6 to 1·8)	1731·6 (1356·2 to 2233·0)	−37·5% (−49·7 to −21·6)
The Bahamas		1·0 (0·6 to 1·7)	3·5 (2·0 to 6·0)	−18·7% (−55·4 to 44·4)	0·3 (0·3 to 0·4)	178·8 (129·4 to 253·1)	2·2% (−23·9 to 40·8)	4·2 (3·4 to 5·2)	1·1 (0·9 to 1·3)	6·6% (−15·1 to 34·0)	1·1 (1 to 1·1)	402·7 (314·4 to 507·1)	12·3% (−3·6 to 31·2)
Trinidad and Tobago		8·6 (5·3 to 14·7)	8·9 (5·5 to 15·2)	−34·1% (−58·1 to 2·7)	1·1 (1 to 1·2)	1033·4 (721·6 to 1574·7)	−24·4% (−45·6 to 6·4)	27·7 (22·1 to 36·2)	2·0 (1·6 to 2·7)	−14·0% (−30·1 to 6·4)	3·4 (3·2 to 3·5)	1908·7 (1486·8 to 2499·9)	−13·8% (−29·3 to 6·0)
Venezuela		596·3 (491·8 to 715·2)	20·2 (16·6 to 24·2)	−40·0% (−51·4 to −26·2)	37·9 (33·5 to 43·2)	61 418·6 (52 002·8 to 73 130·7)	−35·7% (−45·9 to −23·4)	1393·1 (1183·3 to 1677·3)	4·5 (3·8 to 5·4)	−21·6% (−32·1 to −7·8)	83·5 (78·8 to 88·9)	88 292·3 (76 265·7 to 103 889·0)	−26·7% (−36·0 to −15·9)
Virgin Islands		0·1 (0·1 to 0·1)	1·2 (0·9 to 1·7)	−40·6% (−59·6 to −14·8)	0·1 (0·1 to 0·1)	28·5 (21·3 to 37·4)	−11·2% (−23·7 to 1·1)	1·1 (0·9 to 1·4)	1·0 (0·8 to 1·3)	14·8% (−10·2 to 50·3)	0·3 (0·3 to 0·3)	85·6 (65·1 to 108·8)	7·7% (−0·5 to 14·8)
**North Africa and Middle East**		**23 676·5 (18 183·5 to 30 620·0)**	**36·8 (28·3 to 47·6)**	**−48·0% (−60·0 to −33·6)**	**1070·1 (954·5 to 1204·5)**	**2310 442·0 (1836 155·8 to 2923 972·1)**	**−44·5% (−56·0 to −30·6)**	**30 112·9 (24 528·6 to 37 380·3)**	**5·3 (4·3 to 6·6)**	**−42·8% (−53·9 to −29·8)**	**1696·1 (1579·4 to 1830·2)**	**2666 966·9 (2167 412·7 to 3310 493·4)**	**−41·5% (−52·7 to −28·5)**
Afghanistan		6137·8 (3609·0 to 9482·5)	122·0 (71·7 to 188·5)	−56·8% (−74·7 to −30·1)	99·8 (88·2 to 116·5)	551 832·6 (333 450·7 to 834 283·1)	−55·8% (−73·5 to −30·1)	6747·2 (4178·1 to 10 042·4)	20·7 (12·8 to 30·8)	−55·0% (−72·3 to −29·7)	140·2 (128·4 to 156·5)	589 645·6 (367 169·7 to 871 883·3)	−54·5% (−71·8 to −29·6)
Algeria		601·9 (345·8 to 996·6)	13·2 (7·6 to 21·9)	−26·2% (−59·8 to 32·4)	69 (60·9 to 76·8)	69 839·4 (46 317·4 to 104 229·0)	−16·1% (−47·1 to 33·3)	1493·5 (1193·6 to 1921·1)	3·8 (3·0 to 4·8)	−3·2% (−28·7 to 29·9)	112·6 (104·6 to 121·1)	97 412·6 (74 290·3 to 132 499·3)	−11·6% (−36·9 to 24·3)
Bahrain		3·0 (1·9 to 4·1)	3·0 (1·9 to 4·1)	−43·4% (−60·0 to −20·0)	1 (0·9 to 1·1)	518·6 (387·9 to 654·9)	−20·6% (−35·5 to −2·9)	4·9 (3·7 to 6·1)	0·4 (0·3 to 0·4)	−32·2% (−46·3 to −14·0)	1·9 (1·8 to 2)	787·7 (606·6 to 997·2)	−8·0% (−22·4 to 6·7)
Egypt		4023·4 (2701·4 to 6121·3)	34·2 (23·0 to 52·1)	−48·7% (−67·2 to −20·1)	196·6 (170·4 to 224·7)	397 177·0 (281 871·6 to 582 449·5)	−44·9% (−62·4 to −17·8)	4780·3 (3469·1 to 6887·3)	5·2 (3·8 to 7·6)	−45·2% (−62·4 to −20·3)	306·7 (279·9 to 334·4)	448 805·8 (329 449·7 to 637 148·4)	−42·2% (−59·1 to −17·2)
Iran		238·0 (127·5 to 414·6)	3·5 (1·9 to 6·1)	−60·9% (−80·3 to −27·0)	81·9 (70 to 95)	42 064·0 (29 407·8 to 59 838·5)	−40·9% (−59·9 to −11·8)	872·8 (707·3 to 1083·5)	1·1 (0·9 to 1·4)	−35·5% (−53·6 to −14·8)	154·7 (142·5 to 168·1)	73 825·9 (57 919·6 to 94 064·1)	−31·4% (−47·2 to −11·7)
Iraq		1239·9 (728·6 to 1912·5)	21·7 (12·7 to 33·4)	−41·2% (−64·7 to −4·4)	90·6 (76·2 to 103·9)	130 383·7 (83 718·9 to 189 611·9)	−34·8% (−58·1 to −1·0)	1846·5 (1302·2 to 2534·3)	5·1 (3·6 to 7·0)	−33·7% (−54·0 to −6·5)	133·7 (119·1 to 147)	164 043·1 (116 444·7 to 223 389·5)	−30·1% (−51·4 to −1·1)
Jordan		24·4 (15·4 to 36·9)	2·5 (1·6 to 3·9)	−46·0% (−67·7 to −13·0)	13·5 (11·8 to 15·5)	5663·5 (4071·8 to 7342·9)	−16·2% (−36·5 to 5·6)	41·6 (31·9 to 53·8)	0·5 (0·4 to 0·7)	−35·5% (−53·5 to −10·7)	19·7 (18·1 to 21·7)	7621·2 (5577·3 to 9900·8)	−10·0% (−27·7 to 8·4)
Kuwait		4·1 (2·9 to 5·8)	1·1 (0·8 to 1·6)	2·0% (−30·7 to 50·5)	2·7 (2·5 to 3)	1085·0 (814·7 to 1421·6)	44·3% (20·7 to 67·8)	7·3 (5·8 to 9·7)	0·2 (0·1 to 0·2)	13·2% (−12·9 to 44·5)	4·8 (4·6 to 5)	1680·1 (1259·0 to 2162·9)	52·8% (33·9 to 70·5)
Lebanon		6·6 (3·1 to 13·1)	1·8 (0·8 to 3·5)	−49·6% (−75·5 to 5·5)	5·1 (4·4 to 5·9)	1915·7 (1354·9 to 2692·0)	−18·1% (−39·8 to 9·6)	36·4 (26·7 to 48·4)	0·6 (0·5 to 0·8)	−2·1% (−28·2 to 31·7)	10·6 (9·9 to 11·4)	3657·4 (2742·1 to 4802·7)	−6·1% (−23·0 to 10·6)
Libya		32·5 (16·8 to 54·5)	4·9 (2·5 to 8·2)	−49·7% (−72·3 to −10·3)	9·3 (7·8 to 10·8)	5242·0 (3578·3 to 7405·6)	−33·1% (−55·0 to −2·9)	70·1 (53·1 to 93·7)	1·1 (0·8 to 1·5)	−28·1% (−50·4 to 3·0)	15 (13·4 to 16·4)	7575·2 (5648·0 to 9940·4)	−24·2% (−44·9 to 0·3)
Morocco		294·2 (176·9 to 464·0)	8·6 (5·2 to 13·6)	−57·1% (−75·8 to −24·5)	56·3 (49·3 to 64·7)	40 039·9 (28 807·1 to 56 682·5)	−45·4% (−63·3 to −17·0)	829·5 (655·2 to 1051·4)	2·4 (1·9 to 3·1)	−32·9% (−50·9 to −8·8)	105·9 (98·8 to 114·6)	65 851·1 (52 215·4 to 83 832·3)	−35·3% (−51·3 to −14·7)
Oman		3·7 (2·2 to 6·0)	1·0 (0·6 to 1·6)	26·1% (−30·6 to 131·5)	3·9 (3·4 to 4·3)	1343·3 (955·9 to 1809·8)	44·2% (20·3 to 70·3)	25·7 (21·0 to 31·0)	0·6 (0·5 to 0·7)	54·5% (22·0 to 93·8)	6·9 (6·4 to 7·4)	2656·4 (2043·6 to 3375·8)	57·6% (40·4 to 75·3)
Palestine		16·5 (10·7 to 25·0)	2·3 (1·5 to 3·5)	−39·9% (−64·3 to 0·9)	7·9 (6·9 to 9·2)	3518·5 (2649·3 to 4623·2)	−13·1% (−34·6 to 13·5)	28·7 (22·4 to 37·4)	0·6 (0·5 to 0·8)	−22·0% (−45·9 to 9·9)	12 (10·8 to 13·3)	4994·8 (3886·3 to 6451·7)	−3·5% (−22·7 to 18·9)
Qatar		0·9 (0·5 to 1·6)	0·8 (0·4 to 1·4)	6·1% (−47·8 to 111·7)	1·2 (1 to 1·3)	388·7 (270·9 to 528·0)	65·6% (34·6 to 100·5)	2·6 (2·0 to 3·4)	0·1 (0·1 to 0·2)	45·4% (3·3 to 107·8)	1·9 (1·8 to 2·1)	639·3 (470·7 to 850·8)	86·9% (62·0 to 115·3)
Saudi Arabia		103·5 (79·8 to 131·6)	3·4 (2·6 to 4·3)	−37·0% (−51·9 to −17·8)	33·1 (28·9 to 37·8)	17 585·8 (13 929·2 to 22 038·2)	−20·0% (−32·7 to −6·7)	270·2 (239·2 to 304·3)	0·9 (0·8 to 1·0)	−15·7% (−26·6 to −3·6)	54·3 (50·1 to 59)	26 751·1 (21 647·9 to 32 828·5)	−12·0% (−22·3 to −2·4)
Sudan		7620·5 (4273·1 to 12 528·4)	124·8 (70·0 to 205·2)	−40·6% (−63·9 to −0·9)	156·4 (134·9 to 186·4)	693 071·7 (402 663·6 to 1120 449·1)	−39·3% (−61·7 to −0·9)	8706·3 (5315·0 to 13 714·0)	21·6 (13·2 to 34·0)	−38·3% (−60·1 to −3·5)	217·7 (196·2 to 247·4)	752 128·0 (459 781·3 to 1182 960·3)	−37·8% (−59·7 to −3·0)
Syria		49·7 (29·2 to 81·9)	2·2 (1·3 to 3·5)	−3·3% (−49·4 to 92·8)	27·6 (24·2 to 31·5)	11 523·2 (8574·8 to 15 492·2)	−10·2% (−29·1 to 15·9)	85·1 (63·2 to 116·0)	0·5 (0·3 to 0·6)	0·2% (−31·4 to 52·8)	42·5 (38·8 to 46·5)	16 167·6 (12 231·4 to 21 148·4)	−6·9% (−21·8 to 13·4)
Tunisia		37·3 (22·7 to 56·0)	3·8 (2·3 to 5·7)	−55·5% (−75·5 to −21·9)	13·5 (11·7 to 15·5)	6765·0 (4759·8 to 8898·6)	−34·4% (−55·1 to −9·2)	157·4 (123·6 to 195·8)	1·4 (1·1 to 1·7)	−26·6% (−44·0 to −6·8)	25·8 (24 to 27·8)	11 832·4 (9123·0 to 14 828·0)	−26·7% (−42·7 to −10·2)
Turkey		244·2 (137·5 to 435·9)	3·8 (2·2 to 6·8)	−71·4% (−84·5 to −45·8)	88·1 (79 to 98·6)	44 243·7 (31 322·3 to 61 062·4)	−54·9% (−69·5 to −34·5)	548·8 (432·7 to 743·8)	0·7 (0·6 to 0·9)	−59·0% (−70·1 to −42·5)	166·1 (156·5 to 176·7)	69 081·5 (52 057·4 to 89 892·1)	−47·7% (−61·1 to −31·7)
United Arab Emirates		2·4 (1·1 to 4·7)	0·5 (0·2 to 1·0)	−34·5% (−71·3 to 42·6)	5·5 (4·8 to 6·3)	1680·6 (1164·9 to 2276·8)	41·7% (17·1 to 66·0)	43·7 (34·2 to 56·0)	0·5 (0·4 to 0·6)	68·5% (27·1 to 124·4)	9·3 (8·6 to 10·1)	3600·5 (2782·9 to 4605·0)	75·6% (52·5 to 101·3)
Yemen		2974·1 (1513·8 to 4803·6)	74·3 (37·8 to 120·0)	−41·1% (−69·4 to 29·9)	106·1 (93·6 to 120·2)	282 788·3 (155 704·0 to 441 693·1)	−38·0% (−65·6 to 29·0)	3491·0 (2024·1 to 5319·1)	13·0 (7·5 to 19·8)	−38·0% (−64·4 to 24·9)	152·5 (140·3 to 166·2)	316 091·6 (186 650·7 to 473 608·5)	−35·5% (−61·6 to 27·7)
**South Asia**		**143 342·5 (121 458·5 to 167 803·4)**	**85·7 (72·6 to 100·3)**	**−40·7% (−50·5 to −29·4)**	**3212·6 (2941·1 to 3548)**	**13 069 605·0 (11 148 814·4 to 15 169 895·6)**	**−39·2% (−48·7 to −28·1)**	**571 867·0 (524 215·7 to 625 240·6)**	**33·8 (31·0 to 37·0)**	**−22·1% (−29·0 to −14·2)**	**8986·2 (8653·4 to 9389)**	**25 939 993·0 (23 659 621·0 to 28 384 384·7)**	**−31·0% (−38·0 to −23·4)**
Bangladesh		3826·7 (2503·7 to 5622·8)	25·0 (16·4 to 36·8)	−60·4% (−75·3 to −39·1)	279·5 (243·6 to 313·2)	396 211·8 (278 623·2 to 556 197·1)	−55·7% (−70·4 to −35·6)	19 982·7 (16 865·4 to 23 663·4)	12·4 (10·5 to 14·7)	−32·4% (−44·3 to −19·3)	762·2 (724 to 800·5)	814 082·8 (666 252·8 to 995 015·5)	−44·8% (−56·0 to −30·9)
Bhutan		26·8 (10·1 to 53·8)	40·5 (15·2 to 81·4)	−60·1% (−77·9 to −31·7)	3·5 (3·2 to 4)	3154·3 (1666·5 to 5490·0)	−51·3% (−68·9 to −22·0)	96·3 (63·9 to 138·6)	12·4 (8·3 to 17·9)	−32·7% (−48·6 to −9·3)	11 (10·6 to 11·6)	6822·4 (4826·3 to 9315·7)	−32·0% (−49·5 to −8·3)
India		104 643·0 (89 525·9 to 122 375·6)	84·2 (72·1 to 98·5)	−43·2% (−52·3 to −32·2)	2079·6 (1883·7 to 2318)	9478 080·8 (8144 313·2 to 11 032 693·2)	−42·0% (−50·9 to −31·2)	488 999·5 (443 135·5 to 542 962·6)	37·3 (33·8 to 41·4)	−21·7% (−29·3 to −13·1)	6300·3 (6066·8 to 6588·8)	20 666 209·6 (18 833 450·6 to 22 742 931·6)	−32·1% (−38·8 to −24·5)
Nepal		1256·9 (819·7 to 1792·5)	44·2 (28·8 to 63·0)	−70·7% (−80·7 to −55·0)	92·7 (82·4 to 104·3)	130 482·2 (91 434·2 to 178 328·0)	−66·7% (−77·1 to −51·5)	7288·3 (5555·6 to 9172·3)	25·5 (19·5 to 32·1)	−34·4% (−46·2 to −18·6)	262·3 (250·4 to 275·6)	337 131·9 (264 607·3 to 409 635·3)	−47·2% (−56·4 to −34·5)
Pakistan		33 589·2 (22 981·8 to 46 133·5)	135·3 (92·6 to 185·8)	−22·6% (−47·5 to 15·2)	757·3 (694·3 to 830)	3061 675·9 (2154 845·4 to 4136 608·7)	−20·8% (−45·1 to 15·5)	55 500·3 (43 257·7 to 70 027·0)	29·4 (22·9 to 37·0)	−18·9% (−37·1 to 5·0)	1650·4 (1580·3 to 1732·7)	4115 746·4 (3146 982·0 to 5278 901·5)	−18·6% (−38·8 to 8·2)
**Southeast Asia, east Asia, and Oceania**		**16 805·8 (12 991·0 to 20 932·4)**	**11·5 (8·9 to 14·3)**	**−57·5% (−68·6 to −42·5)**	**1138·8 (1036·2 to 1243·7)**	**1739 482·9 (1403 744·0 to 2098 801·8)**	**−52·9% (−63·5 to −38·6)**	**88 066·6 (77 411·0 to 99 546·0)**	**4·2 (3·7 to 4·8)**	**−30·1% (−39·0 to −20·7)**	**3083·5 (2966·8 to 3197·5)**	**3789 582·4 (3310 849·2 to 4281 169·5)**	**−40·7% (−48·8 to −30·9)**
American Samoa		0·1 (0·1 to 0·2)	1·3 (0·9 to 1·8)	−27·6% (−49·8 to 4·4)	0·1 (0·1 to 0·1)	30·1 (22·3 to 38·3)	1·7% (−15·6 to 23·4)	1·2 (1·0 to 1·5)	1·4 (1·1 to 1·8)	11·3% (−11·6 to 40·8)	0·2 (0·2 to 0·2)	89·5 (70·9 to 110·8)	22·4% (9·4 to 34·9)
Cambodia		459·6 (249·5 to 782·9)	25·7 (14·0 to 43·8)	−71·7% (−85·0 to −50·6)	26·4 (23·3 to 30·3)	46 334·2 (28 636·2 to 73 138·1)	−68·5% (−81·5 to −48·3)	1256·0 (977·2 to 1629·4)	8·1 (6·3 to 10·5)	−59·1% (−69·3 to −46·1)	65·3 (61·8 to 69·4)	76 642·4 (56 805·5 to 105 743·3)	−62·3% (−73·8 to −46·8)
China		1919·7 (1558·2 to 2353·5)	2·3 (1·9 to 2·8)	−71·0% (−77·2 to −62·5)	248·2 (224·7 to 277·7)	230 847·4 (192 875·5 to 276 969·8)	−64·5% (−71·0 to −56·6)	5851·5 (5373·9 to 6348·6)	0·4 (0·4 to 0·5)	−59·8% (−63·8 to −55·5)	641·3 (616·8 to 671·1)	418 739·7 (357 730·0 to 487 899·5)	−58·5% (−63·6 to −53·0)
Federated States of Micronesia		0·4 (0·2 to 0·7)	3·0 (1·4 to 5·9)	−55·3% (−77·6 to −2·9)	0·1 (0·1 to 0·1)	59·5 (39·5 to 90·2)	−43·2% (−63·5 to −9·8)	2·7 (1·8 to 3·9)	2·5 (1·7 to 3·8)	−32·8% (−49·3 to −9·8)	0·3 (0·3 to 0·4)	167·7 (125·6 to 220·0)	−27·8% (−42·8 to −11·3)
Fiji		26·3 (13·6 to 49·1)	29·9 (15·5 to 55·9)	−2·0% (−50·5 to 86·7)	0·9 (0·8 to 1)	2476·3 (1366·3 to 4460·6)	−2·4% (−46·6 to 76·9)	89·0 (70·6 to 115·1)	10·0 (7·9 to 12·9)	19·1% (−9·7 to 56·9)	3 (2·9 to 3·1)	4705·4 (3473·1 to 6720·3)	4·9% (−24·1 to 49·6)
Guam		0·2 (0·1 to 0·3)	1·3 (0·9 to 1·8)	25·0% (−15·7 to 84·4)	0·1 (0·1 to 0·1)	35·2 (26·7 to 45·7)	18·0% (−1·5 to 41·9)	1·8 (1·4 to 2·3)	1·0 (0·8 to 1·4)	63·5% (23·1 to 115·0)	0·3 (0·3 to 0·3)	118·8 (93·1 to 148·9)	40·7% (28·9 to 53·0)
Indonesia		8559·4 (4911·2 to 12 391·6)	34·5 (19·8 to 50·0)	−50·5% (−73·8 to −6·1)	478·8 (423·9 to 526·8)	859 607·1 (556 464·5 to 1200 465·2)	−45·5% (−68·2 to −3·0)	56 929·1 (47 274·5 to 67 214·9)	22·1 (18·4 to 26·1)	−20·8% (−36·5 to −2·7)	1177·8 (1120·5 to 1233·1)	2047 754·7 (1675 262·9 to 2454 145·9)	−32·9% (−48·7 to −10·7)
Kiribati		7·3 (3·0 to 15·4)	48·8 (20·1 to 103·2)	−37·9% (−74·8 to 35·2)	0·2 (0·2 to 0·2)	668·1 (301·7 to 1357·7)	−36·5% (−71·4 to 33·3)	23·9 (18·2 to 33·3)	21·2 (16·2 to 29·6)	−16·9% (−38·8 to 15·2)	0·5 (0·5 to 0·5)	1195·1 (810·3 to 1921·7)	−26·5% (−53·1 to 18·7)
Laos		811·8 (434·3 to 1359·8)	97·1 (51·9 to 162·7)	−57·1% (−76·7 to −18·7)	10 (8·7 to 11·7)	72 275·5 (40 011·5 to 119 402·9)	−56·1% (−75·8 to −18·4)	1159·6 (770·4 to 1704·1)	17·1 (11·3 to 25·1)	−54·6% (−71·0 to −27·8)	23·4 (22 to 25·3)	87 855·1 (55 114·0 to 134 683·3)	−54·9% (−72·2 to −23·7)
Malaysia		50·2 (29·3 to 85·3)	2·1 (1·2 to 3·5)	−35·9% (−63·2 to 10·3)	21·2 (17·9 to 24·9)	9952·2 (7295·4 to 13 423·2)	−13·8% (−33·5 to 14·0)	385·1 (317·1 to 466·2)	1·3 (1·0 to 1·5)	0·4% (−19·2 to 22·0)	60·4 (56·9 to 64·2)	28 305·0 (22 840·1 to 34 849·5)	2·9% (−10·0 to 16·8)
Maldives		2·0 (1·2 to 3·3)	5·4 (3·2 to 8·9)	−53·8% (−75·2 to −14·0)	0·3 (0·3 to 0·4)	259·1 (177·5 to 366·5)	−42·1% (−63·0 to −6·8)	7·6 (6·0 to 9·4)	2·1 (1·7 to 2·6)	−26·0% (−44·0 to −2·3)	0·8 (0·8 to 0·9)	484·7 (383·3 to 611·3)	−31·3% (−48·1 to −8·8)
Marshall Islands		0·7 (0·3 to 1·3)	7·0 (3·1 to 14·4)	−65·6% (−85·5 to −15·9)	0·1 (0·1 to 0·1)	79·2 (46·4 to 138·5)	−57·9% (−78·8 to −13·7)	2·6 (1·9 to 3·4)	3·5 (2·7 to 4·7)	−36·1% (−56·1 to −7·8)	0·3 (0·2 to 0·3)	169·2 (126·3 to 232·3)	−40·2% (−61·2 to −10·2)
Mauritius		4·4 (2·8 to 5·7)	6·2 (3·9 to 8·1)	−28·4% (−54·5 to 4·7)	0·6 (0·5 to 0·7)	537·8 (385·2 to 673·7)	−26·8% (−46·6 to −3·3)	26·7 (18·2 to 32·0)	2·1 (1·4 to 2·5)	15·6% (−26·1 to 41·9)	2·4 (2·3 to 2·5)	1364·4 (1107·1 to 1622·9)	−6·0% (−22·1 to 8·9)
Myanmar		1292·1 (714·5 to 2155·9)	27·5 (15·2 to 45·9)	−72·7% (−86·1 to −51·5)	50·6 (45·9 to 56·5)	123 803·8 (73 735·7 to 196 053·8)	−70·9% (−84·0 to −50·4)	7714·2 (5397·0 to 10 369·9)	14·3 (10·0 to 19·2)	−43·9% (−58·9 to −25·2)	163 (156·8 to 169·7)	314 766·3 (233 988·8 to 421 123·3)	−55·3% (−67·9 to −40·0)
North Korea		63·6 (23·3 to 156·8)	3·6 (1·3 to 9·0)	−62·0% (−88·1 to 25·4)	16·4 (14·2 to 18·9)	9875·3 (5814·6 to 17 896·2)	−50·6% (−76·1 to 2·2)	199·3 (143·5 to 301·9)	0·8 (0·6 to 1·2)	−39·5% (−64·8 to 0·1)	40 (37·6 to 42·5)	19 267·5 (13 672·7 to 28 140·0)	−37·7% (−61·8 to −4·6)
Northern Mariana Islands		0·1 (0·0 to 0·1)	0·7 (0·3 to 1·2)	−9·0% (−57·2 to 83·0)	0 (0 to 0·1)	17·4 (12·2 to 23·3)	22·9% (−3·9 to 55·0)	0·5 (0·4 to 0·6)	0·4 (0·4 to 0·6)	38·3% (9·9 to 71·7)	0·2 (0·2 to 0·2)	64·7 (49·3 to 82·5)	56·6% (41·3 to 71·3)
Papua New Guinea		373·8 (166·9 to 709·3)	37·0 (16·5 to 70·2)	−52·5% (−76·9 to −6·6)	10·6 (9·5 to 12·2)	34 697·4 (16 900·6 to 63 634·0)	−50·6% (−74·7 to −6·5)	2253·4 (1598·9 to 3157·7)	29·5 (20·9 to 41·4)	−14·3% (−35·8 to 14·5)	31·5 (30 to 33·2)	92 344·8 (65 710·7 to 132 198·8)	−30·1% (−50·0 to −3·2)
Philippines		2864·3 (2223·3 to 3600·0)	25·2 (19·6 to 31·7)	−46·6% (−58·8 to −29·9)	142·7 (127·8 to 160·5)	281 533·9 (224 667·9 to 348 885·9)	−44·0% (−55·5 to −28·8)	5728·4 (4343·6 to 7045·0)	5·7 (4·3 to 7·0)	−35·0% (−48·6 to −21·1)	371·1 (353·4 to 390·4)	446 810·3 (368 593·2 to 530 626·5)	−36·5% (−46·3 to −24·8)
Samoa		0·3 (0·1 to 0·7)	1·1 (0·3 to 3·0)	−55·9% (−82·7 to 5·6)	0·2 (0·2 to 0·3)	82·1 (54·3 to 126·3)	−28·0% (−51·9 to 0·4)	4·6 (3·3 to 6·6)	2·4 (1·7 to 3·4)	−7·0% (−31·7 to 30·0)	0·6 (0·6 to 0·7)	252·1 (191·9 to 327·1)	−10·5% (−27·3 to 5·3)
Seychelles		0·1 (0·1 to 0·1)	1·2 (0·8 to 1·7)	−31·9% (−51·8 to −4·9)	0·1 (0·1 to 0·1)	23·3 (17·8 to 30·7)	−11·2% (−24·7 to 4·2)	1·3 (1·0 to 1·6)	1·3 (1·1 to 1·7)	−11·4% (−30·7 to 13·3)	0·2 (0·2 to 0·2)	80·5 (64·5 to 99·9)	−2·3% (−10·9 to 6·2)
Solomon Islands		12·3 (6·1 to 21·6)	14·6 (7·2 to 25·7)	−42·5% (−69·8 to 29·5)	0·9 (0·8 to 1)	1282·6 (751·6 to 2084·4)	−37·7% (−63·3 to 22·4)	40·9 (29·3 to 56·2)	7·0 (5·0 to 9·6)	−28·5% (−48·3 to −1·3)	2·5 (2·4 to 2·6)	2498·4 (1832·5 to 3405·6)	−28·6% (−49·2 to 5·2)
Sri Lanka		18·4 (13·0 to 26·6)	1·1 (0·8 to 1·6)	−76·8% (−83·6 to −67·3)	10 (8·9 to 11·3)	4125·7 (3061·4 to 5277·3)	−58·3% (−66·6 to −49·8)	437·1 (227·3 to 697·7)	2·1 (1·1 to 3·4)	−49·5% (−70·2 to −22·3)	45·4 (43·7 to 47·1)	19 096·5 (13 896·1 to 25 722·8)	−37·6% (−49·2 to −25·0)
Taiwan		6·9 (3·3 to 13·7)	0·7 (0·3 to 1·3)	28·5% (−39·5 to 165·9)	4·3 (3·9 to 4·9)	1755·6 (1252·2 to 2428·5)	2·0% (−20·9 to 38·1)	115·4 (90·6 to 144·2)	0·5 (0·4 to 0·6)	49·4% (14·8 to 89·7)	14·3 (13·8 to 14·9)	5482·3 (4373·2 to 6797·9)	9·0% (−1·7 to 25·3)
Thailand		48·5 (28·8 to 79·2)	1·3 (0·8 to 2·1)	−70·8% (−82·4 to −51·0)	29·7 (25·5 to 34·2)	12 059·2 (8742·2 to 16 132·5)	−47·3% (−59·1 to −32·9)	2675·4 (2131·9 to 3293·1)	3·9 (3·1 to 4·9)	0·6% (−20·6 to 26·4)	146·5 (141·5 to 151·9)	87 581·7 (72 715·1 to 103 263·6)	−19·9% (−30·1 to −8·3)
Timor-Leste		112·1 (39·2 to 236·2)	53·7 (18·8 to 113·2)	−59·4% (−85·9 to 8·3)	2·9 (2·6 to 3·3)	10 388·2 (4077·9 to 20 989·1)	−57·5% (−83·1 to 8·1)	155·1 (78·7 to 281·3)	13·0 (6·6 to 23·6)	−54·2% (−77·9 to −1·6)	5·6 (5·3 to 6·1)	12 335·1 (5959·0 to 22 996·5)	−55·4% (−79·4 to 0·7)
Tonga		0·5 (0·3 to 0·9)	3·7 (1·9 to 6·5)	−41·7% (−69·3 to 5·8)	0·1 (0·1 to 0·1)	73·9 (48·8 to 106·8)	−30·2% (−54·0 to 2·9)	3·2 (2·5 to 4·2)	3·0 (2·4 to 3·9)	−13·6% (−33·9 to 14·2)	0·4 (0·3 to 0·4)	176·2 (138·2 to 220·0)	−16·1% (−32·3 to 0·3)
Vanuatu		6·4 (3·3 to 11·4)	19·5 (10·0 to 34·8)	−39·0% (−71·6 to 47·9)	0·4 (0·4 to 0·5)	659·0 (378·2 to 1093·1)	−34·4% (−65·8 to 38·3)	18·2 (12·6 to 25·3)	6·9 (4·8 to 9·6)	−24·1% (−47·0 to 11·0)	1·3 (1·3 to 1·4)	1215·7 (876·0 to 1674·3)	−23·1% (−47·8 to 22·2)
Vietnam		132·5 (73·2 to 227·7)	1·7 (0·9 to 2·9)	−50·8% (−74·8 to −1·5)	81·2 (68·6 to 93·3)	32 830·6 (23 384·9 to 45 314·0)	−28·9% (−49·9 to −5·2)	2517·3 (1762·2 to 3538·9)	2·7 (1·9 to 3·8)	−23·9% (−48·5 to 11·9)	279·5 (266·6 to 292·7)	107 791·8 (83 999·2 to 135 233·1)	−17·5% (−30·3 to −5·5)
**Sub-saharan Africa**		**303 045·1 (260 960·9 to 348 617·3)**	**191·6 (165·0 to 220·4)**	**−25·2% (−37·3 to −10·9)**	**3311·9 (2999 to 3689·9)**	**26 735 862·9 (23 150 003·6 to 30 641 750·4)**	**−24·6% (−36·4 to −10·5)**	**569 147·9 (514 949·4 to 632 041·3)**	**59·3 (53·6 to 65·8)**	**−16·9% (−26·0 to −5·5)**	**8014·2 (7658·4 to 8440·3)**	**37 033 384·0 (33 172 726·7 to 41 364 493·9)**	**−20·0% (−29·9 to −8·0)**
Angola		6116·2 (3320·1 to 10 161·6)	123·6 (67·1 to 205·4)	−29·3% (−61·2 to 26·6)	123·1 (109 to 139·1)	554 953·0 (317 493·2 to 903 146·9)	−27·9% (−58·7 to 25·3)	10 897·8 (6207·4 to 19 268·3)	43·2 (24·6 to 76·3)	−20·4% (−51·7 to 27·0)	246·5 (231·8 to 263·7)	776 472·6 (480 410·2 to 1193 913·1)	−22·1% (−51·2 to 23·7)
Benin		2279·5 (1395·6 to 3466·8)	130·8 (80·1 to 199·0)	−32·4% (−57·7 to 7·4)	26·4 (23·9 to 29·4)	201 591·5 (125 635·3 to 302 384·8)	−32·0% (−57·0 to 6·6)	4401·6 (2996·0 to 6128·2)	40·3 (27·4 to 56·1)	−19·8% (−44·0 to 13·6)	68·4 (65·3 to 71·7)	291 806·7 (202 073·4 to 398 444·7)	−24·4% (−47·0 to 7·2)
Botswana		144·4 (73·2 to 241·1)	54·9 (27·8 to 91·6)	−39·1% (−64·5 to −1·4)	4·4 (3·8 to 5)	13 462·0 (7409·5 to 21 710·8)	−36·9% (−61·1 to −0·9)	619·4 (287·5 to 1634·5)	27·4 (12·7 to 72·3)	−24·8% (−64·9 to 74·5)	12 (11·3 to 12·7)	31 665·3 (16 710·2 to 71 675·8)	−29·8% (−61·3 to 31·4)
Burkina Faso		7863·2 (5187·8 to 11 487·4)	251·0 (165·6 to 366·7)	−25·8% (−53·3 to 11·9)	77·1 (66 to 92·5)	690 903·5 (461 593·4 to 1000 212·3)	−25·3% (−52·8 to 11·4)	11 975·9 (8713·2 to 15 962·8)	66·2 (48·2 to 88·2)	−19·3% (−43·4 to 12·2)	166 (154·2 to 181·6)	865 168·7 (630 845·8 to 1185 243·4)	−21·0% (−45·4 to 11·0)
Burundi		4093·8 (2371·5 to 6555·7)	192·2 (111·3 to 307·8)	−11·0% (−47·4 to 51·3)	55·1 (48 to 66·3)	363 461·3 (216 259·4 to 574 260·1)	−10·0% (−45·8 to 49·9)	8310·3 (5682·3 to 11 549·4)	73·9 (50·5 to 102·7)	−9·1% (−38·1 to 27·3)	128·6 (120·8 to 140·7)	529 406·3 (357 471·7 to 742 469·7)	−8·3% (−37·4 to 32·9)
Cameroon		6258·2 (3672·2 to 9581·5)	164·9 (96·8 to 252·5)	−24·4% (−54·5 to 25·8)	88·2 (78·8 to 101·9)	557 630·0 (336 578·0 to 843 271·3)	−24·0% (−53·2 to 24·0)	10 085·7 (6925·9 to 13 896·7)	43·1 (29·6 to 59·4)	−23·5% (−47·0 to 10·4)	207·9 (197·1 to 221·3)	725 980·2 (494 335·8 to 1006 694·6)	−22·2% (−46·0 to 14·3)
Cape Verde		9·7 (6·1 to 15·6)	18·2 (11·5 to 29·2)	−51·4% (−72·9 to −9·5)	0·9 (0·7 to 1)	1050·8 (708·7 to 1567·0)	−47·2% (−67·8 to −11·7)	30·6 (25·1 to 37·5)	5·9 (4·8 to 7·2)	−41·0% (−56·8 to −18·5)	2·8 (2·7 to 2·9)	2049·9 (1639·3 to 2616·7)	−38·6% (−55·5 to −16·9)
Central African Republic		1697·6 (975·3 to 2767·3)	238·7 (137·1 to 389·1)	5·6% (−44·8 to 106·8)	18·8 (16·5 to 22)	150 158·8 (88 012·8 to 241 541·0)	5·1% (−43·4 to 100·4)	4453·1 (2826·9 to 6432·6)	90·8 (57·7 to 131·2)	14·2% (−26·0 to 70·4)	44·4 (42·1 to 47·7)	255 730·0 (166 050·5 to 364 652·4)	12·1% (−26·7 to 73·8)
Chad		15 781·7 (10 407·8 to 21 969·7)	593·7 (391·6 to 826·5)	8·9% (−25·5 to 60·2)	73·1 (65·5 to 83·4)	1365 268·3 (906 113·0 to 1892 831·9)	8·9% (−25·0 to 59·3)	20 404·6 (14 352·0 to 26 710·7)	145·1 (102·1 to 189·9)	6·9% (−23·1 to 47·9)	154·8 (146·8 to 165·2)	1571 879·9 (1087 907·8 to 2098 327·8)	9·0% (−22·3 to 54·9)
Comoros		84·0 (41·8 to 150·4)	68·4 (34·0 to 122·5)	−44·8% (−71·4 to 14·9)	2·6 (2·3 to 3)	7876·6 (4238·6 to 13 610·3)	−42·3% (−68·1 to 14·5)	323·3 (216·7 to 449·8)	40·8 (27·4 to 56·8)	−10·2% (−39·5 to 33·1)	7·6 (7·2 to 8)	16 930·9 (11 780·1 to 23 363·2)	−21·6% (−47·4 to 24·3)
Congo (Brazzaville)		373·9 (192·0 to 672·5)	48·7 (25·0 to 87·6)	−33·1% (−65·1 to 22·5)	17·4 (15·3 to 20)	36 408·1 (20 718·0 to 61 676·6)	−29·4% (−60·4 to 22·9)	1189·9 (773·4 to 1757·4)	25·7 (16·7 to 38·0)	−14·9% (−43·0 to 26·6)	38·2 (35·9 to 40·8)	69 096·1 (46 586·4 to 100 371·0)	−18·2% (−44·4 to 18·2)
Côte d'Ivoire		5985·6 (3620·8 to 8933·9)	163·8 (99·1 to 244·5)	−16·0% (−49·1 to 39·5)	84·1 (74·4 to 95·3)	534 417·1 (330 742·1 to 790 067·0)	−15·5% (−48·0 to 37·9)	10 266·2 (7243·7 to 13 848·8)	45·2 (31·9 to 61·0)	−11·6% (−39·0 to 24·5)	203·8 (193·3 to 216·4)	725 035·0 (508 292·7 to 986 063·1)	−11·9% (−38·6 to 29·1)
DR Congo		19 117·5 (11 386·8 to 29 651·3)	136·3 (81·2 to 211·4)	−18·3% (−52·5 to 38·8)	451·3 (397 to 508·3)	1751 494·5 (1081 983·8 to 2652 323·1)	−16·4% (−50·1 to 38·1)	35 624·4 (24 846·3 to 48 812·3)	46·0 (32·1 to 63·1)	−9·9% (−35·2 to 26·4)	853·4 (801·8 to 913·4)	2451 530·4 (1723 931·9 to 3432 384·7)	−11·6% (−38·5 to 28·5)
Djibouti		128·2 (61·7 to 201·4)	122·9 (59·1 to 193·1)	−46·9% (−73·0 to −8·4)	2 (1·8 to 2·4)	11 475·9 (5806·1 to 17 711·2)	−45·9% (−71·2 to −8·5)	405·7 (249·5 to 642·0)	45·6 (28·0 to 72·1)	−19·4% (−51·0 to 37·9)	7·6 (7·3 to 8)	21 607·6 (13 742·9 to 31 544·7)	−31·4% (−56·1 to 8·4)
Equatorial Guinea		64·2 (29·4 to 121·3)	50·1 (22·9 to 94·6)	−35·6% (−69·1 to 35·7)	2·7 (2·3 to 3·1)	6193·9 (3134·3 to 11 084·5)	−32·1% (−64·4 to 34·1)	144·2 (77·8 to 302·9)	17·1 (9·2 to 35·8)	−21·6% (−56·5 to 35·3)	6·5 (6·1 to 6·9)	9956·1 (5927·2 to 17 329·9)	−22·7% (−53·9 to 30·8)
Eritrea		1788·7 (1050·9 to 2710·2)	215·7 (126·7 to 326·8)	−16·4% (−48·6 to 30·0)	16 (14·1 to 19·1)	156 636·5 (93 568·2 to 235 183·5)	−16·0% (−47·7 to 29·2)	4759·9 (3022·3 to 7094·2)	90·8 (57·7 to 135·3)	9·7% (−23·0 to 51·4)	44·9 (42·5 to 47·8)	270 182·5 (186 479·9 to 376 819·4)	−2·1% (−30·1 to 30·9)
Ethiopia		14 662·4 (8367·3 to 22 488·9)	100·1 (57·1 to 153·6)	−63·2% (−79·2 to −33·1)	267·6 (235·8 to 304·3)	1321 515·1 (790 948·9 to 1985 265·0)	−62·1% (−77·9 to −32·2)	46 786·1 (32 822·4 to 63 577·3)	47·1 (33·0 to 63·9)	−40·3% (−59·2 to −13·3)	804·1 (766·2 to 846)	2450 575·1 (1753 401·5 to 3316 658·7)	−49·9% (−64·9 to −24·4)
Gabon		68·6 (35·7 to 122·8)	28·6 (14·9 to 51·2)	−40·4% (−67·5 to 14·1)	4·6 (4·1 to 5·2)	7064·0 (4095·8 to 11 648·7)	−35·5% (−61·7 to 12·8)	294·4 (176·7 to 449·9)	17·1 (10·2 to 26·1)	−27·9% (−52·1 to 9·0)	11·6 (11 to 12·2)	15 234·1 (10 329·4 to 21 277·1)	−26·5% (−48·8 to 5·2)
Ghana		1698·1 (951·0 to 2682·3)	41·8 (23·4 to 66·0)	−41·5% (−66·8 to −3·5)	77·6 (68·9 to 86·9)	164 857·7 (99 272·7 to 248 251·1)	−38·7% (−62·2 to −3·5)	3849·7 (2619·2 to 5413·1)	14·0 (9·6 to 19·7)	−33·5% (−53·9 to −5·9)	205·6 (195·2 to 216·2)	271 270·2 (195 346·3 to 365 145·2)	−31·8% (−50·4 to −7·6)
Guinea		2984·6 (1878·3 to 4389·9)	148·4 (93·4 to 218·3)	−32·4% (−57·4 to 7·7)	45 (40·3 to 50·2)	266 500·4 (171 441·5 to 389 267·0)	−31·6% (−56·2 to 7·3)	5999·5 (4383·7 to 7841·1)	47·7 (34·9 to 62·4)	−19·9% (−42·4 to 9·7)	107·1 (101·8 to 112·6)	391 461·1 (284 077·4 to 519 750·0)	−23·9% (−45·3 to 6·6)
Guinea-Bissau		1204·9 (767·1 to 1753·0)	411·3 (261·8 to 598·4)	−14·4% (−45·7 to 31·4)	6·3 (5·5 to 7·6)	104 378·8 (66 903·6 to 150 724·6)	−14·3% (−45·3 to 30·8)	1682·1 (1043·9 to 2581·4)	91·0 (56·5 to 139·6)	−10·4% (−41·0 to 32·6)	15·1 (14·2 to 16·4)	125 110·6 (82 375·4 to 175 481·6)	−11·7% (−41·4 to 27·8)
Kenya		8915·5 (6858·6 to 11 128·5)	122·0 (93·9 to 152·3)	−28·9% (−39·8 to −16·9)	121·6 (108·4 to 138·7)	794 310·0 (618 078·8 to 985 907·2)	−28·1% (−38·7 to −16·3)	33 624·4 (29 662·4 to 38 323·7)	72·8 (64·2 to 83·0)	−5·0% (−14·0 to 5·3)	363·4 (346·9 to 383·6)	1614 755·6 (1399 232·1 to 1847 865·1)	−14·0% (−22·3 to −4·8)
Lesotho		803·9 (565·2 to 1113·6)	295·0 (207·4 to 408·6)	−33·4% (−53·5 to −8·1)	5·9 (5·3 to 7)	70 426·0 (49 886·6 to 97 368·0)	−33·1% (−52·7 to −8·2)	1845·1 (1319·5 to 2567·9)	86·7 (62·0 to 120·6)	−28·6% (−48·1 to −5·2)	14·5 (13·8 to 15·6)	108 693·0 (79 602·0 to 143 969·7)	−29·5% (−47·5 to −8·3)
Liberia		1225·2 (750·4 to 1795·7)	173·5 (106·3 to 254·3)	−36·4% (−60·5 to −0·5)	17·5 (15·7 to 19·9)	109 419·7 (68 809·5 to 158 384·3)	−35·4% (−59·3 to −0·4)	2358·0 (1682·3 to 3135·2)	52·3 (37·3 to 69·5)	−23·0% (−46·6 to 9·6)	43·8 (41·7 to 46·4)	154 832·0 (109 999·9 to 207 750·4)	−27·9% (−50·9 to 3·3)
Madagascar		6672·3 (4200·5 to 10 049·2)	179·0 (112·7 to 269·6)	−14·8% (−48·2 to 36·0)	79·4 (70·2 to 91)	590 625·9 (377 901·5 to 877 806·8)	−14·1% (−47·0 to 35·3)	13 624·8 (9392·6 to 18 370·9)	56·3 (38·8 to 75·9)	2·4% (−29·6 to 43·8)	225·9 (215·6 to 239·5)	890 306·0 (636 210·6 to 1204 622·6)	−3·9% (−33·7 to 35·7)
Malawi		5400·3 (3424·2 to 8032·0)	182·9 (116·0 to 272·0)	−22·7% (−50·4 to 17·8)	71·2 (59·3 to 88·6)	479 530·1 (310 959·6 to 705 047·0)	−22·3% (−49·1 to 16·8)	11 246·1 (8216·4 to 14 999·7)	65·3 (47·7 to 87·1)	−17·2% (−40·7 to 15·9)	172·9 (160·4 to 191·2)	690 123·7 (498 827·9 to 927 761·1)	−19·4% (−41·6 to 11·7)
Mali		7311·4 (4606·5 to 10 942·2)	225·7 (142·2 to 337·8)	−18·2% (−48·6 to 26·9)	61·2 (55 to 68·1)	640 588·8 (410 767·9 to 951 603·4)	−17·9% (−47·9 to 26·0)	11 031·5 (7875·8 to 14 852·5)	62·8 (44·8 to 84·5)	−11·6% (−38·0 to 23·9)	135·9 (129·2 to 143·5)	783 234·2 (548 382·4 to 1096 832·5)	−13·8% (−41·0 to 24·9)
Mauritania		708·9 (457·0 to 1031·8)	114·7 (73·9 to 166·9)	−39·6% (−62·0 to −7·4)	13·7 (12·2 to 15·3)	63 963·4 (42 280·8 to 91 715·2)	−38·4% (−60·1 to −7·3)	1240·9 (910·4 to 1648·0)	30·4 (22·3 to 40·3)	−30·3% (−48·7 to −4·5)	33·7 (31·9 to 35·4)	87 082·0 (64 461·7 to 116 077·5)	−32·8% (−50·9 to −7·5)
Mozambique		4383·0 (2550·0 to 6857·7)	90·8 (52·8 to 142·0)	−44·8% (−68·7 to −7·9)	76·7 (68·8 to 86·6)	394 626·3 (240 315·2 to 605 072·0)	−43·7% (−67·2 to −8·1)	13 896·7 (8917·7 to 19 801·6)	49·6 (31·9 to 70·7)	−15·0% (−46·1 to 26·3)	213·8 (204 to 224·9)	736 991·6 (513 362·6 to 996 700·3)	−27·6% (−51·2 to 2·2)
Namibia		405·3 (240·6 to 622·3)	122·0 (72·4 to 187·3)	−11·8% (−50·6 to 47·0)	7·1 (6·2 to 8)	36 493·6 (22 156·5 to 55 372·7)	−10·6% (−47·6 to 45·7)	825·1 (567·7 to 1133·9)	33·6 (23·1 to 46·2)	−26·6% (−49·6 to 5·4)	16·5 (15·6 to 17·5)	52 587·8 (36 223·5 to 73 050·6)	−19·9% (−45·8 to 15·7)
Niger		19 868·4 (13 540·1 to 27 759·1)	484·6 (330·3 to 677·1)	−16·6% (−42·3 to 23·5)	100·9 (91·7 to 117·1)	1718 067·7 (1179 119·7 to 2392 924·7)	−16·2% (−41·8 to 23·2)	25 221·5 (18 429·6 to 33 494·2)	127·0 (92·8 to 168·7)	−12·2% (−36·9 to 21·6)	203 (193 to 218·6)	1955 127·5 (1411 198·9 to 2635 702·1)	−13·8% (−38·2 to 20·9)
Nigeria		102 676·3 (72 764·8 to 136 171·0)	327·6 (232·2 to 434·5)	−20·2% (−45·4 to 16·8)	504·5 (458·2 to 566·3)	8896 509·9 (6339 728·2 to 11 759 454·7)	−20·0% (−45·0 to 16·4)	143 688·7 (110 833·9 to 182 165·5)	78·7 (60·7 to 99·8)	−20·2% (−40·9 to 11·2)	1166·9 (1116·7 to 1232·2)	10 608 997·7 (8049 712·0 to 13 617 151·7)	−19·7% (−42·0 to 12·2)
Rwanda		1590·7 (847·1 to 2619·8)	92·7 (49·4 to 152·6)	−48·3% (−71·5 to −10·0)	34·6 (31·1 to 39·1)	144 657·4 (80 366·9 to 233 980·4)	−47·3% (−69·6 to −11·2)	3419·2 (2254·0 to 4967·8)	29·4 (19·4 to 42·7)	−33·0% (−54·0 to −1·1)	101·1 (97 to 106·4)	221 947·9 (148 658·1 to 319 454·1)	−38·3% (−58·4 to −9·6)
São Tomé and Príncipe		19·5 (12·2 to 29·4)	64·5 (40·2 to 97·1)	−51·7% (−70·2 to −21·5)	0·7 (0·6 to 0·8)	1857·2 (1210·7 to 2706·1)	−49·2% (−67·1 to −20·3)	35·2 (25·0 to 47·2)	18·4 (13·1 to 24·7)	−41·5% (−59·0 to −17·7)	1·8 (1·7 to 1·9)	2654·0 (1924·5 to 3514·5)	−41·4% (−58·1 to −16·1)
Senegal		4281·7 (2931·1 to 5879·4)	165·9 (113·6 to 227·8)	−43·1% (−60·7 to −18·4)	65·7 (59·4 to 72·3)	381 413·4 (267 104·8 to 517 474·7)	−42·1% (−59·8 to −17·9)	7382·5 (5451·3 to 9880·1)	48·9 (36·1 to 65·4)	−29·6% (−49·5 to −3·2)	140·5 (133·7 to 147·6)	508 324·3 (387 802·7 to 654 295·5)	−35·1% (−51·6 to −12·0)
Sierra Leone		2189·0 (1232·3 to 3411·3)	216·0 (121·6 to 336·6)	−39·4% (−65·1 to −0·8)	21·4 (19·4 to 24·1)	192 794·8 (110 213·9 to 296 653·9)	−38·8% (−64·2 to −1·0)	3426·6 (2421·9 to 4724·2)	53·0 (37·5 to 73·1)	−35·4% (−56·1 to −6·8)	53·2 (50·9 to 56·2)	250 597·1 (169 606·4 to 357 249·5)	−35·8% (−57·2 to −4·6)
Somalia		9140·7 (6032·5 to 12 821·2)	449·6 (296·7 to 630·6)	3·6% (−29·1 to 61·4)	41·7 (38·5 to 45)	790 940·5 (526 312·9 to 1103 839·9)	3·7% (−28·7 to 60·4)	19 738·3 (11 519·2 to 33 461·5)	181·9 (106·2 to 308·4)	9·1% (−25·8 to 71·9)	106·4 (102·3 to 110·6)	1180 409·6 (771 609·6 to 1756 029·8)	7·1% (−23·6 to 55·2)
South Africa		3026·2 (2311·3 to 3901·1)	56·8 (43·4 to 73·2)	−70·1% (−77·6 to −60·4)	76·8 (68·2 to 85·8)	278 589·2 (215 849·3 to 352 805·6)	−68·6% (−75·9 to −58·9)	13 447·1 (11 909·0 to 15 060·3)	25·0 (22·2 to 28·0)	−41·3% (−47·9 to −33·2)	250·1 (241 to 261·1)	603 602·0 (525 308·5 to 695 861·9)	−54·3% (−60·8 to −46·3)
South Sudan		7313·9 (4648·5 to 10 442·3)	384·2 (244·2 to 548·5)	41·5% (−15·4 to 190·0)	48·3 (42 to 55·3)	637 044·2 (410 416·8 to 904 237·4)	41·0% (−14·6 to 182·4)	17 578·1 (9835·9 to 33 311·9)	143·1 (80·0 to 271·1)	49·6% (−12·9 to 162·6)	150·3 (142·4 to 158·6)	1003 469·9 (641 331·3 to 1638 115·2)	46·2% (−8·9 to 153·8)
Swaziland		362·0 (227·4 to 528·3)	206·8 (129·9 to 301·8)	−50·9% (−69·5 to −27·2)	3·5 (3·1 to 4)	31 940·2 (20 330·8 to 46 116·0)	−50·2% (−68·4 to −27·0)	684·1 (460·6 to 974·5)	53·1 (35·7 to 75·6)	−39·3% (−57·8 to −13·6)	8·2 (7·7 to 8·7)	44 938·3 (31 379·2 to 61 671·3)	−44·4% (−60·9 to −23·2)
Tanzania		5852·2 (3634·8 to 8937·7)	62·9 (39·1 to 96·1)	−28·9% (−55·7 to 12·9)	179·8 (159·1 to 203·6)	547 025·7 (361 044·6 to 812 132·6)	−26·8% (−52·4 to 12·7)	23 427·8 (15 717·0 to 35 028·4)	43·9 (29·4 to 65·6)	−1·9% (−34·7 to 46·1)	491·3 (467·8 to 517·7)	1168 718·8 (846 242·0 to 1617 761·5)	−12·3% (−37·3 to 19·6)
The Gambia		398·7 (264·1 to 560·3)	106·0 (70·2 to 149·0)	−38·9% (−59·1 to −10·8)	9·3 (8·4 to 10·5)	36 357·0 (24 815·1 to 49 994·2)	−37·2% (−56·4 to −10·2)	545·1 (401·0 to 720·1)	27·2 (20·0 to 36·0)	−31·4% (−49·7 to −7·4)	19·1 (18·1 to 20·3)	44 030·6 (32 795·2 to 58 059·3)	−32·1% (−50·3 to −7·4)
Togo		1559·0 (980·2 to 2282·1)	134·6 (84·6 to 197·0)	−32·7% (−57·0 to 1·5)	28·2 (25·2 to 32·1)	140 295·2 (91 301·6 to 201 846·1)	−31·6% (−55·6 to 1·2)	2863·7 (2082·7 to 3784·6)	39·2 (28·5 to 51·8)	−23·0% (−44·7 to 4·7)	67·6 (64·2 to 71·5)	199 850·0 (143 997·8 to 262 242·9)	−25·1% (−44·8 to 1·2)
Uganda		7894·3 (4941·8 to 11 805·8)	106·7 (66·8 to 159·6)	−6·1% (−42·4 to 50·1)	170·3 (146·9 to 205·3)	718 903·9 (464 100·8 to 1051 099·1)	−5·2% (−39·8 to 47·1)	16 144·2 (11 037·4 to 22 507·4)	41·2 (28·2 to 57·5)	−5·6% (−34·2 to 32·3)	395·7 (370·5 to 430·3)	1062 506·6 (771 647·2 to 1398 299·0)	−3·4% (−30·4 to 37·2)
Zambia		3374·5 (2093·3 to 5156·8)	116·8 (72·5 to 178·6)	−46·9% (−69·3 to −17·2)	66·3 (58·9 to 74·9)	305 451·8 (196 493·9 to 457 269·1)	−45·6% (−67·5 to −16·9)	11 444·1 (8257·5 to 15 218·0)	70·4 (50·8 to 93·7)	−29·9% (−50·6 to −2·9)	174·5 (165·9 to 184)	598 647·9 (447 372·6 to 786 072·2)	−35·4% (−53·7 to −12·4)
Zimbabwe		5222·2 (3866·2 to 6929·2)	210·9 (156·1 to 279·8)	15·3% (−18·6 to 62·2)	60·6 (54·2 to 68·1)	462 731·2 (343 959·4 to 610 113·3)	15·9% (−17·3 to 61·1)	7746·4 (5930·0 to 9784·5)	49·7 (38·1 to 62·8)	1·4% (−23·4 to 34·1)	125·3 (118·4 to 133·4)	585 143·0 (456 316·4 to 739 907·6)	8·4% (−17·7 to 42·8)

Data are from GBD 2015 estimates for both sexes, presented for children younger than 5 years and all ages. Data in parentheses are 95% uncertainty intervals. DALY=disability-adjusted life-year.

Mortality from diarrhoea varied by location. The highest rates of under-5 mortality due to diarrhoea were in sub-Saharan Africa and South Asia, in particular in Chad (594 deaths [95% UI 392–827] per 100 000) and Niger (485 deaths [330–677] per 100 000; figure 1). However, due to their moderate-to-high burden and large populations, India (105 000 deaths, 90 000–122 000) and Nigeria (103 000 deaths, 73 000–136 000) combined had 42% of the 499 000 global under-5 deaths due to diarrhoea in 2015 (table 1).

**Figure 4:**
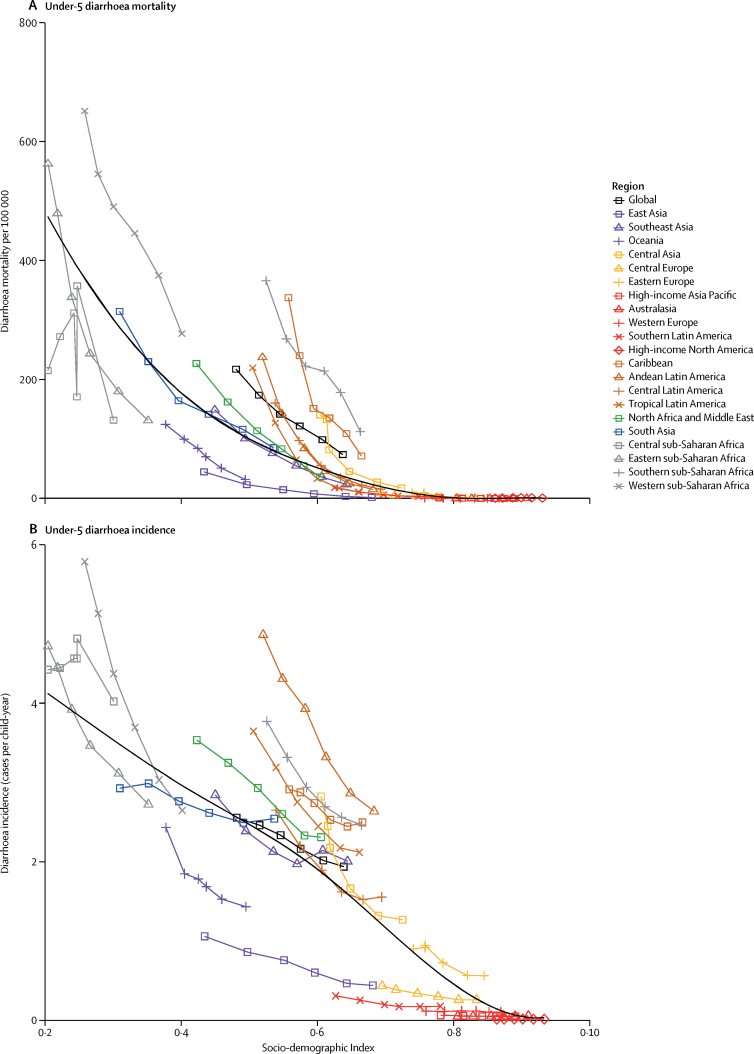
Trends in under-5 diarrhoea mortality and incidence and SDI by region, 1990–2015 The diarrhoea mortality rate per 100 000 population (A) and incidence per child-year (B) for each region is shown. Points represent 5 year increments from 1990 to 2015. The black line is a least-squares cubic spline regression using the age-standardised diarrhoea mortality rate for each geographic location and represents the expected rate based on SDI alone, where estimates above the black line are higher than expected and those below are lower than expected on the basis of SDI alone. More information on the formulation and theory of the SDI is available in the GBD 2015 cause of death capstone paper.^1^ SDI=Socio-demographic Index. GBD=Global Burden of Disease.

The mortality rate due to diarrhoea decreased by 39·2% (95% UI 24·0–51·2) among children younger than 5 years between 2005 (122·1 deaths [109·3–136·5] per 100 000) and 2015 (74·3 deaths [66·6–83·0] per 100 000) but with variation by region, which is shown against the SDI for each region in Figure 4A. Between 2005 and 2015, the fastest reductions in under-5 mortality rate due to diarrhoea were in east Asia and tropical and Andean Latin America (>65% reduction during this time period). The greatest absolute reduction in mortality rate due to diarrhoea was in sub-Saharan Africa. The diarrhoea mortality rate decreased by more than 100 deaths per 100 000 in western sub-Saharan Africa (from 445 to 277 deaths per 100 000), eastern sub-Saharan Africa (from 243 to 131 deaths per 100 000), and southern sub-Saharan Africa (from 214 to 113 deaths per 100 000; figure 3). Under-5 diarrhoea incidence decreased more slowly than did diarrhoea mortality due to diarrhoea (figure 4B). At the global level, diarrhoea incidence in this age group decreased by 10·4% (95% UI 9·1–11·6) between 2005 and 2015. Diarrhoea incidence decreased the fastest in western and eastern sub-Saharan Africa but was largely unchanged in the high-income super-region (figure 4B).

We estimated that there were 2·39 billion (95% UI 2·30 billion to 2·50 billion) episodes of diarrhoea in 2015, of which 957·5 million (871·1 million to 1·0575 billion) occurred in children younger than 5 years (table 1). From 2005 to 2015, diarrhoea incidence decreased by 10·4% (9·1–11·6) in children under-5 and by 5·9% (5·0–6·7) among all ages and both rates of change were less than the declines in mortality rates. In 2015, diarrhoeal diseases caused 71 590 000 DALYs (66 443 000–77 206 000) with most occurring in children younger than 5 years (45 109 000, 40 694 000–50 119 000; table 1). Most DALYs due to diarrhoea are from YLLs (65 858 000 [92·0%] DALYs due to diarrhoea).

**Figure 5:**
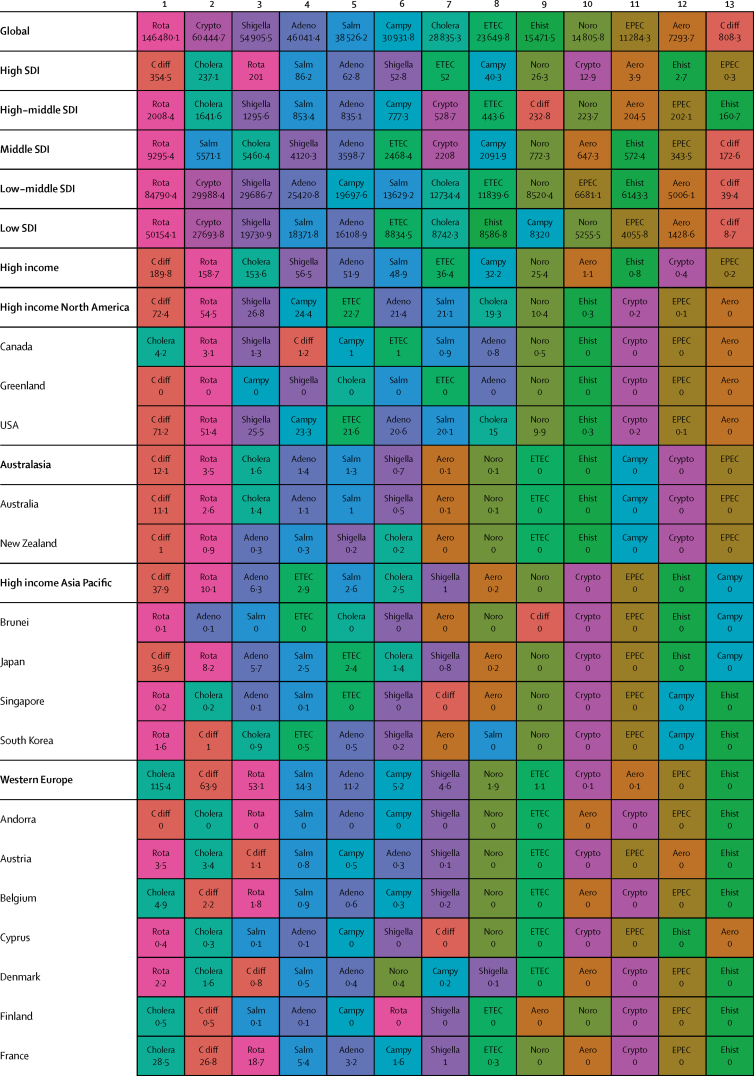

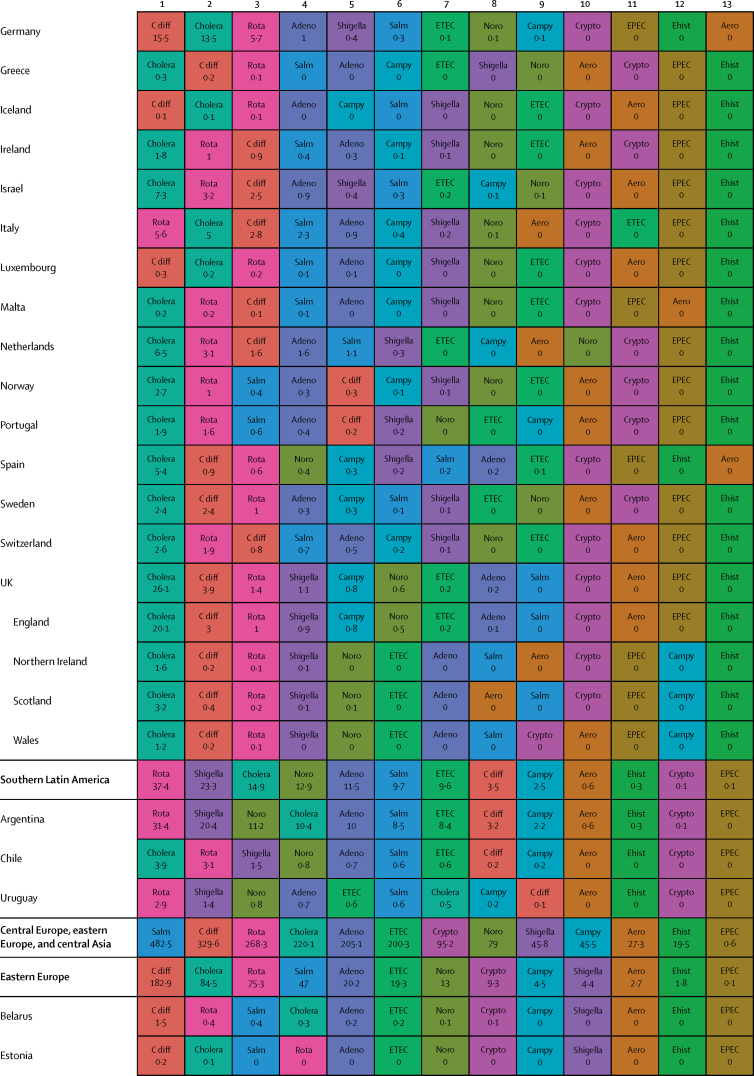

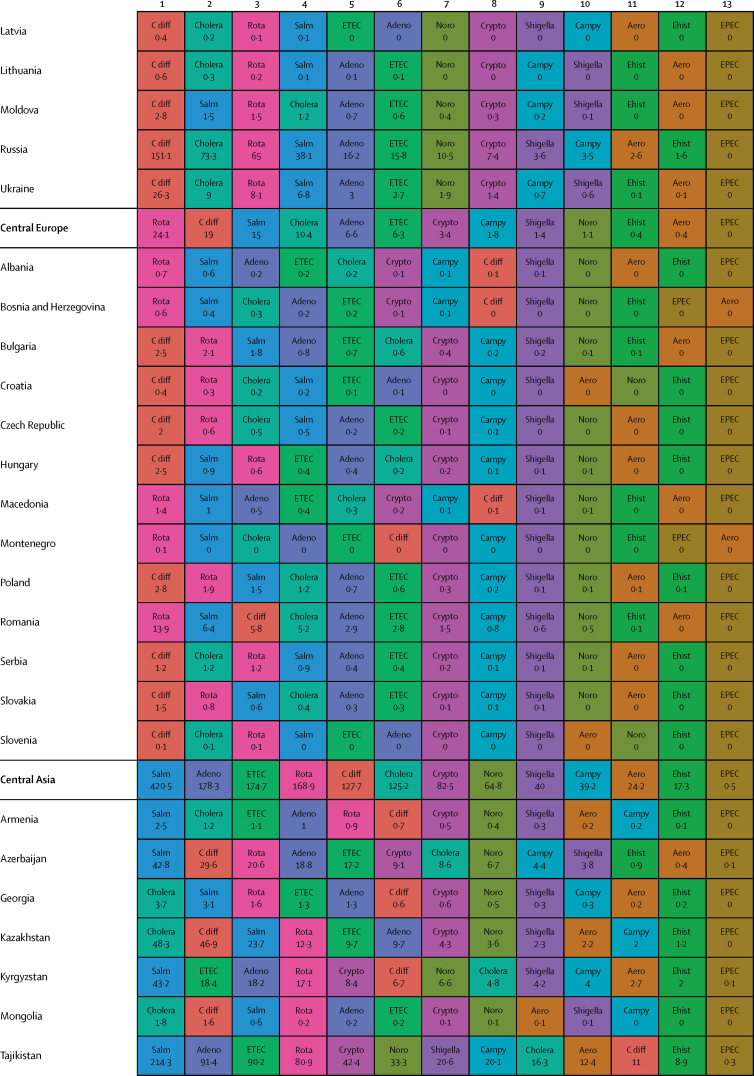

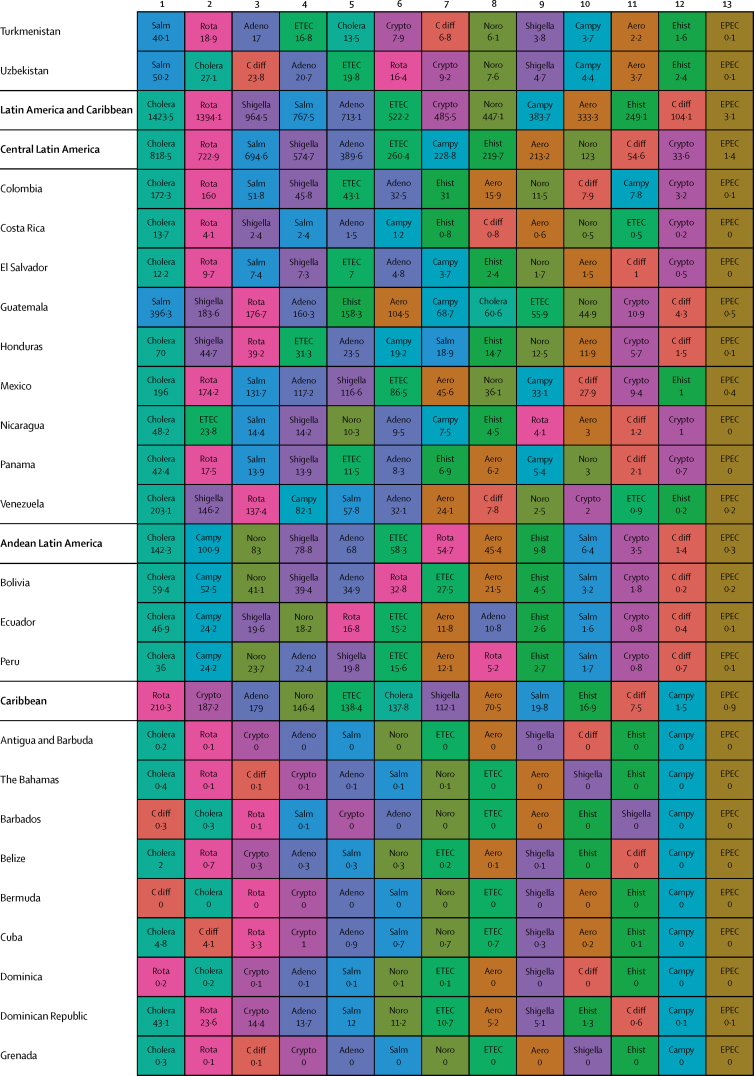

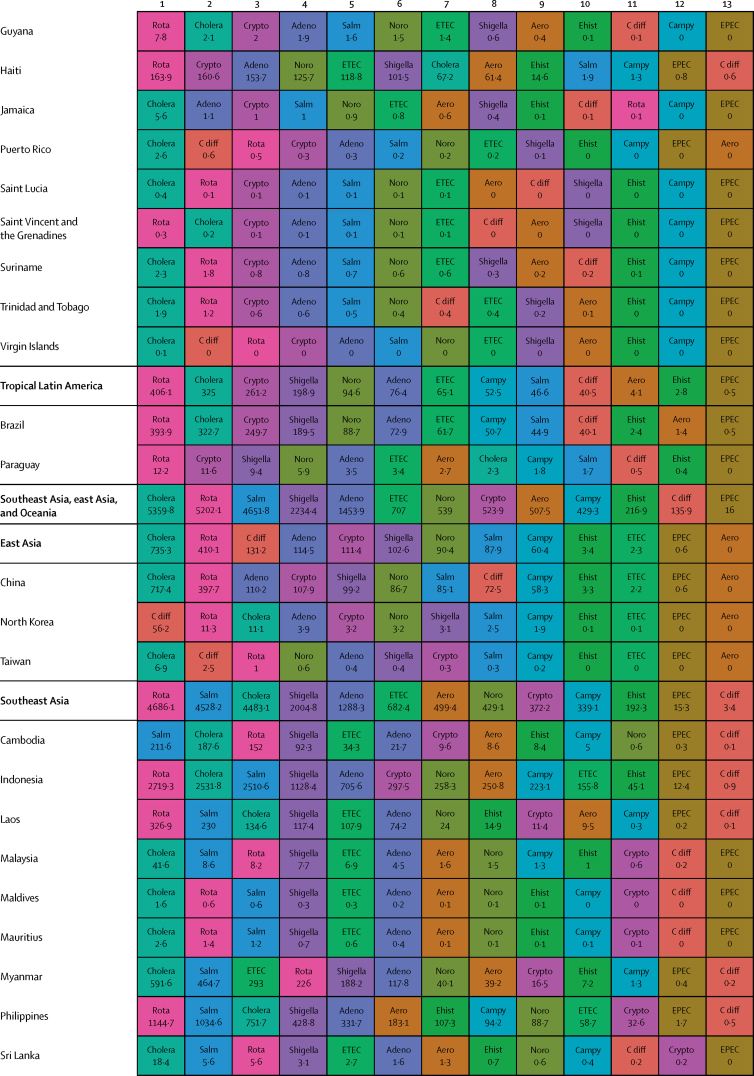

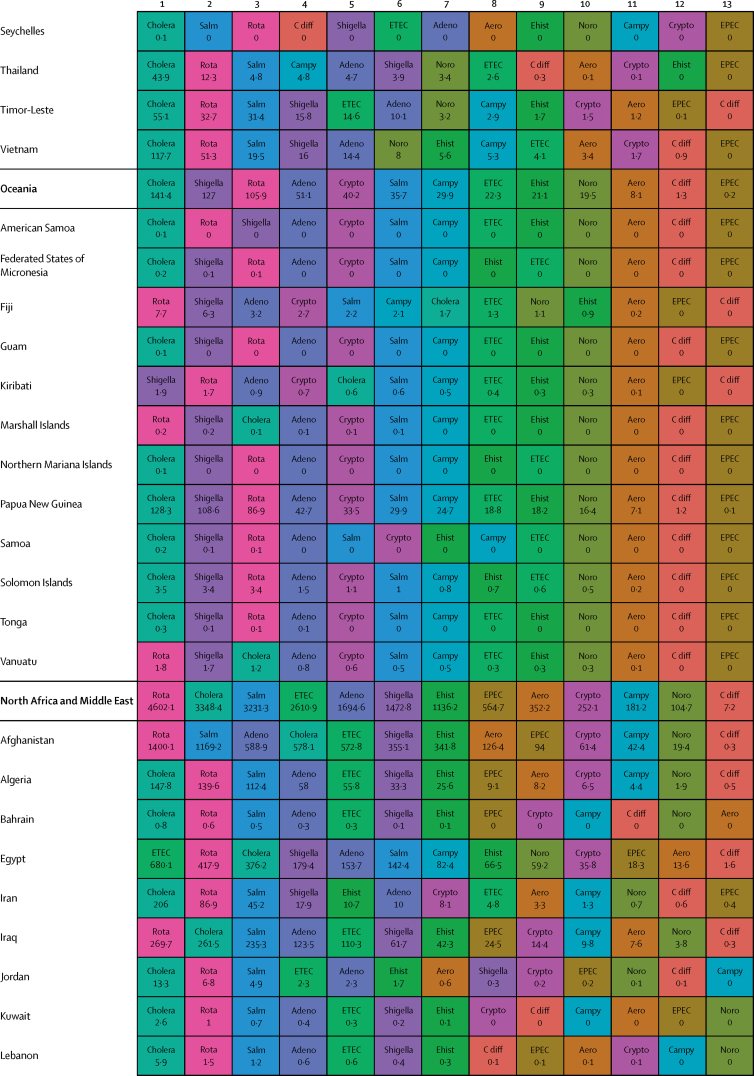

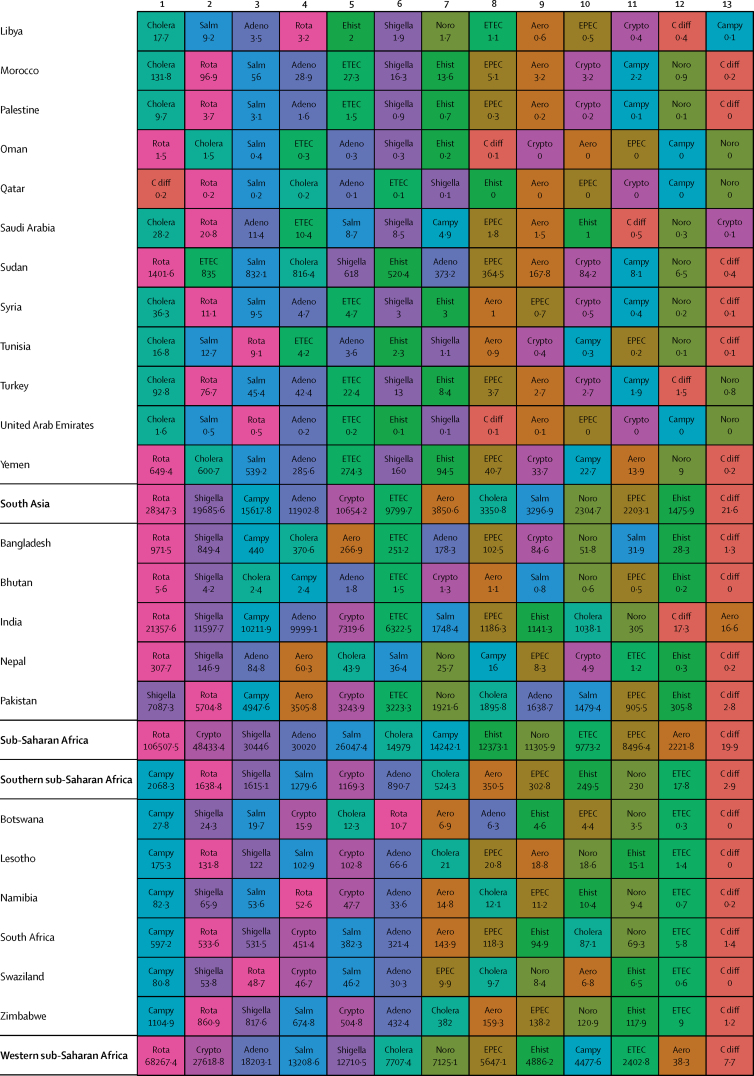

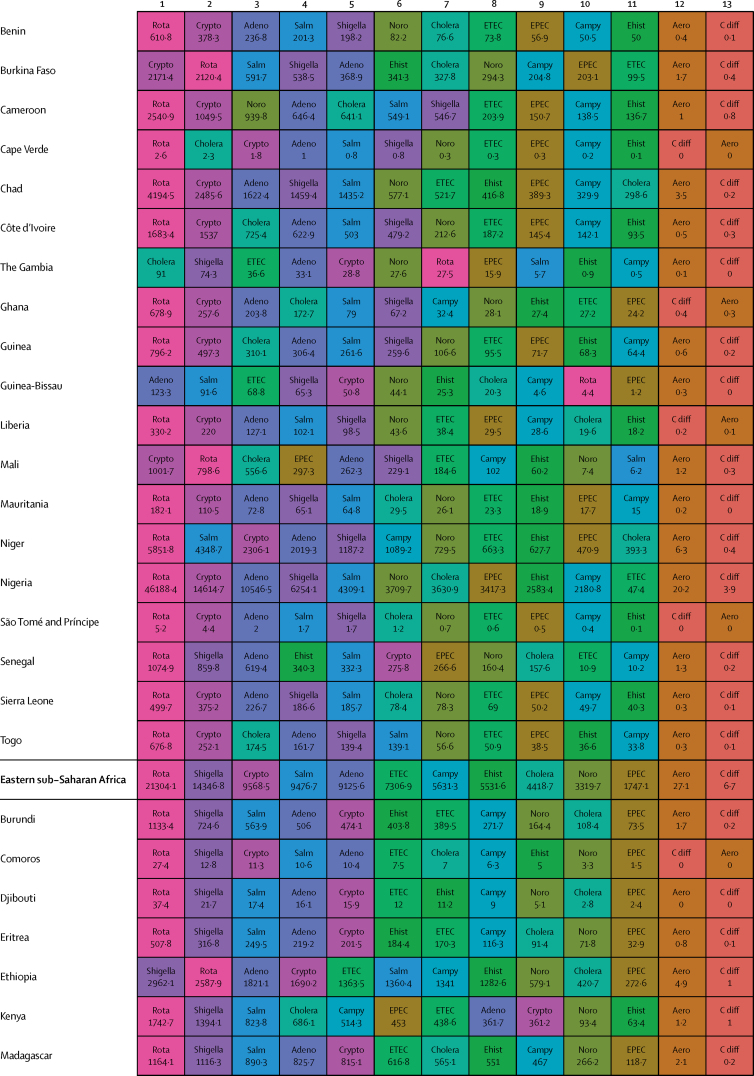

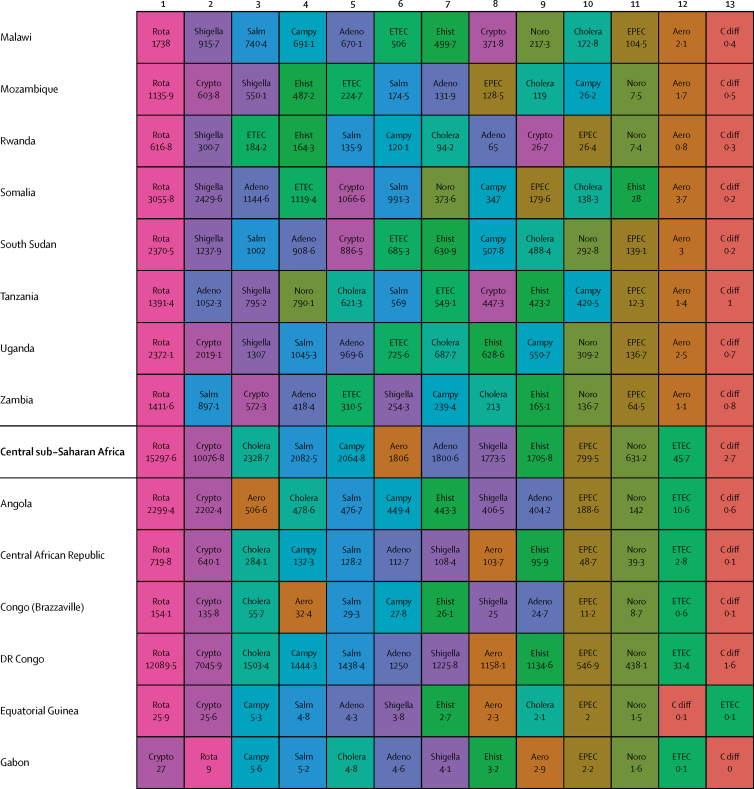
Number of under-5 diarrhoea deaths by aetiology and geography in 2015 Each aetiology is represented by a colour across geographies, ordered left to right by geographical ranking. SDI=Socio-demographic Index. Rota=rotavirus. Crypto=*Cryptosporidium* spp. Shigella=*Shigella* spp. Adeno=adenovirus. Salm=*Salmonella* spp. Campy=*Campylobacter* spp. Cholera=*Vibrio cholerae*. ETEC=enterotoxigenic *Escherichia coli*. Ehist=*Entamoeba histolytica* (amoebiasis). Noro=norovirus. tEPEC=typical enteropathogenic *Escherichia coli*. Aero=*Aeromonas* spp. C diff=*Clostridium difficile.*

Diarrhoeal episodes and deaths were attributed to 13 pathogens in GBD 2015. Aetiologies could be established for 96·1% of all deaths due to diarrhoea in children younger than 5 years and 72·0% of diarrhoeal deaths at all ages. The three most common aetiologies to which diarrhoea mortality was attributed in 2015 among children younger than 5 years were rotavirus (146 000 deaths, 95% UI 118 000–183 000; 29·3%, 24·6–35·9), *Cryptosporidium* spp (60 400 deaths, 13 709·1–134 506·4; 12·1%, 2·8–26·9), and *Shigella* spp (54 900 deaths, 27 000–94 700; 11·0%, 5·5–18·7%;), which combined accounted for more than 50% of deaths due to diarrhoea in this age group (table 2). Figure 5 shows the number of under-5 deaths by aetiology and geography in 2015. Adenovirus was an important cause of death in children younger than 5 years, accounting for 9·2% (3·3–19·7) of deaths due to diarrhoea in this age group (46 000 deaths, 16 200–97 700). Among children aged 5–14 years, *V cholerae* was the leading cause of death (12 814 deaths, 9031–16 943; 24·8%, 17·6–32·3) and *Shigella* spp were the leading cause of death among adults aged 15–99 years (100 013 deaths, 47 119–173 200; 13·1%, 6·7–21·0).

**Table 2:** Deaths due to diarrhoeal disease in children younger than 5 years by aetiology

	**Global**	**India**	**Nigeria**	**Pakistan**	**Niger**	**DR Congo**	**Chad**	**Ethiopia**	**Somalia**	**Kenya**	**Indonesia**
**Adenovirus**											
Number	46 041·4 (16 217·8 to 97 703·3)	9999·1 (4141·7 to 19 713·2)	10 546·5 (2987·1 to 26 037·5)	1638·7 (597·5 to 3660·3)	2019·3 (566·0 to 4904·6)	1250·0 (418·6 to 2735·8)	1622·4 (446·2 to 3933·2)	1821·1 (348·0 to 5304·6)	1144·6 (239·1 to 2988·3)	361·7 (137·1 to 736·6)	705·6 (225·4 to 1633·8)
Percentage	9·2% (3·3 to 19·7)	9·6% (4·0 to 18·7)	10·3% (3·1 to 23·3)	4·9% (1·9 to 10·0)	10·2% (3·0 to 23·6)	6·6% (2·5 to 13·0)	10·3% (3·1 to 23·8)	12·4% (3·1 to 30·1)	12·4% (3·1 to 31·1)	4·1% (1·6 to 8·1)	8·2% (3·0 to 17·3)
**Aeromonas**											
Number	7293·7(−48 278·1 to 59 103·2)	16·6(−68·5 to 92·9)	20·2(−52·0 to 75·3)	3505·8(−21 980·2 to 25 096·6)	6·3(−6·2 to 15·5)	1158·1(−11 215·5 to 11 984·0)	3·5(−7·6 to 12·6)	4·9(−8·4 to 13·9)	3·7(−4·3 to 8·2)	1·2(−5·2 to 6·8)	250·8(−2615·4 to 3118·4)
Percentage	1·4% (−9·7 to 12·0)	0·0% (−0·1 to 0·1)	0·0% (−0·1 to 0·1)	10·4% (−60·1 to 69·2)	0·0% (0·0 to 0·1)	6·3% (−56·6 to 61·6)	0·0% (0·0 to 0·1)	0·0% (−0·1 to 0·1)	0·0% (0·0 to 0·1)	0·0% (−0·1 to 0·1)	2·9% (−32·5 to 34·7)
**Amoebiasis**											
Number	15 471·5(−32 445·3 to 102 357·9)	1141·3(−6879·9 to 13 660·1)	2583·4(−3921·0 to 17 925·3)	305·8(−2101·2 to 3878·8)	627·7(−322·1 to 3467·5)	1134·6(−3550·5 to 7564·8)	416·8(−477·0 to 2696·8)	1282·6(−2706·2 to 8244·0)	28·0(−6·5 to 261·1)	63·4(−180·2 to 635·6)	45·1(−255·6 to 530·3)
Percentage	3·1% (−6·3 to 20·7)	1·1% (−6·0 to 12·9)	2·6% (−3·6 to 17·4)	0·9% (−6·2 to 11·1)	3·2% (−1·6 to 17·7)	6·0% (−17·9 to 44·1)	2·6% (−2·9 to 17·2)	8·8% (−17·2 to 55·0)	0·3% (−0·1 to 2·9)	0·7% (−2·2 to 6·8)	0·5% (−2·9 to 6·6)
***Campylobacter* spp enteritis**											
Number	30 931·8 (8321·5 to 62 515·8)	10 211·9 (3254·9 to 19 104·0)	2180·8(−74·6 to 7547·0)	4947·6 (1314·0 to 10 378·1)	1089·2 (39·5 to 2853·5)	1444·3 (312·8 to 3288·8)	329·9(−23·7 to 1268·5)	1341·0 (262·4 to 2986·5)	347·0 (21·0 to 906·7)	514·3 (128·8 to 1139·6)	223·1 (1·3 to 824·3)
Percentage	6·2% (1·7 to 12·5)	9·7% (3·2 to 17·8)	2·1% (−0·1 to 7·8)	14·7% (4·4 to 27·8)	5·5% (0·2 to 13·7)	7·6% (1·9 to 15·9)	2·1% (−0·1 to 7·9)	9·2% (1·8 to 19·6)	3·8% (0·2 to 9·5)	5·8% (1·3 to 12·4)	2·6% (0·0 to 9·1)
**Cholera**											
Number	28 835·3 (20 612·2 to 39 716·0)	1038·1 (440·4 to 2330·6)	3630·9 (1849·7 to 6494·8)	1895·8 (666·9 to 4623·8)	393·3 (194·0 to 704·0)	1503·4 (575·3 to 3438·0)	298·6 (146·2 to 543·3)	420·7 (138·7 to 1024·2)	138·3 (67·7 to 258·7)	686·1 (436·9 to 1009·9)	2531·8 (953·7 to 4852·3)
Percentage	5·8% (4·1 to 7·9)	1·0% (0·4 to 2·1)	3·5% (2·0 to 6·0)	5·7% (2·1 to 13·4)	2·0% (1·1 to 3·3)	7·9% (3·3 to 16·7)	1·9% (1·0 to 3·4)	2·9% (1·2 to 6·3)	1·5% (0·8 to 2·7)	7·8% (4·8 to 11·8)	29·6% (14·5 to 51·3)
**Clostridium difficile**											
Number	808·3 (701·8 to 932·9)	17·3 (9·4 to 27·5)	3·9 (1·9 to 6·8)	2·8 (1·4 to 4·8)	0·4 (0·1 to 0·7)	1·6 (0·7 to 3·1)	0·2 (0·1 to 0·4)	1·0 (0·3 to 2·1)	0·2 (0·1 to 0·3)	1·0 (0·6 to 1·7)	0·9 (0·3 to 2·0)
Percentage	0·2% (0·1 to 0·2)	0·0% (0·0 to 0·0)	0·0% (0·0 to 0·0)	0·0% (0·0 to 0·0)	0·0% (0·0 to 0·0)	0·0% (0·0 to 0·0)	0·0% (0·0 to 0·0)	0·0% (0·0 to 0·0)	0·0% (0·0 to 0·0)	0·0% (0·0 to 0·0)	0·0% (0·0 to 0·0)
**Cryptosporidiosis**											
Number	60 444·7 (13 709·1 to 134 506·4)	7319·6 (648·3 to 18 136·6)	14 614·7 (2742·3 to 35 386·8)	3243·9 (493·1 to 8139·9)	2306·1 (172·6 to 6074·6)	7045·9 (2147·0 to 14 117·3)	2485·6 (363·8 to 6496·0)	1690·2 (249·0 to 4360·4)	1066·6 (123·3 to 2853·8)	361·2 (6·5 to 991·9)	297·5 (3·5 to 1004·3)
Percentage	12·1% (2·8 to 26·9)	7·0% (0·6 to 17·7)	14·3% (2·7 to 33·5)	9·6% (1·6 to 23·1)	11·7% (0·9 to 30·9)	37·1% (13·5 to 64·1)	15·6% (2·5 to 37·8)	11·6% (1·8 to 27·8)	11·8% (1·4 to 29·5)	4·1% (0·1 to 11·4)	3·5% (0·0 to 11·0)
**Enteropathogenic *E coli* infection**											
Number	11 284·3 (733·6 to 32 034·3)	1186·3 (39·3 to 3756·3)	3417·3 (27·5 to 9893·2)	905·5 (56·9 to 2555·2)	470·9(−44·5 to 1549·9)	546·9 (22·1 to 1565·8)	389·3 (2·9 to 1314·6)	272·6 (2·8 to 978·7)	179·6 (1·9 to 655·7)	453·0 (44·9 to 1116·5)	12·4 (0·3 to 98·9)
Percentage	2·3% (0·1 to 6·2)	1·1% (0·0 to 3·6)	3·3% (0·0 to 9·3)	2·7% (0·1 to 7·4)	2·4% (−0·2 to 7·3)	2·9% (0·1 to 7·8)	2·4% (0·0 to 7·9)	1·8% (0·0 to 6·3)	1·9% (0·0 to 6·4)	5·1% (0·5 to 12·2)	0·1% (0·0 to 1·1)
**Enterotoxigenic** E coli **infection**											
Number	23 649·8 (9553·8 to 44 337·2)	6322·5 (2398·6 to 12 103·4)	47·4 (26·1 to 69·0)	3223·3 (1262·2 to 6603·6)	663·3 (78·0 to 1764·1)	31·4 (4·2 to 203·8)	521·7 (69·8 to 1401·9)	1363·5 (436·2 to 2947·2)	1119·4 (452·2 to 2229·2)	438·6 (162·4 to 863·7)	155·8 (4·3 to 458·9)
Percentage	4·7% (2·0 to 8·9)	6·0% (2·3 to 11·5)	0·0% (0·0 to 0·1)	9·6% (4·2 to 17·2)	3·3% (0·5 to 8·3)	0·2% (0·0 to 1·1)	3·3% (0·5 to 8·1)	9·3% (3·7 to 18·2)	12·2% (5·6 to 22·0)	4·9% (1·8 to 9·6)	1·8% (0·1 to 5·1)
**Norovirus**											
Number	14 805·8 (4161·0 to 33 675·0)	305·0 (33·8 to 1598·5)	3709·7 (742·3 to 9151·2)	1921·6 (619·8 to 4319·4)	729·5 (109·4 to 1871·4)	438·1 (79·8 to 1126·3)	577·1 (88·9 to 1498·4)	579·1 (47·7 to 1571·4)	373·6 (33·5 to 1069·1)	93·4 (6·2 to 291·0)	258·3 (43·5 to 648·2)
Percentage	3·0% (0·8 to 6·7)	0·3% (0·0 to 1·5)	3·6% (0·7 to 8·9)	5·7% (2·1 to 12·1)	3·7% (0·5 to 9·4)	2·3% (0·5 to 5·3)	3·6% (0·7 to 9·1)	4·0% (0·4 to 10·2)	4·0% (0·3 to 10·7)	1·0% (0·1 to 3·1)	3·0% (0·6 to 7·1)
**Other *Salmonella* spp infections**											
Number	38 526·2 (12 214·6 to 84 247·0)	1748·4 (272·7 to 4998·4)	4309·1 (723·7 to 10 611·6)	1479·4 (239·2 to 3617·8)	4348·7 (1369·7 to 9044·0)	1438·4 (382·7 to 3140·4)	1435·2 (308·8 to 3763·9)	1360·4 (322·9 to 3230·7)	991·3 (279·3 to 2273·0)	823·8 (267·6 to 1835·7)	2510·6 (657·5 to 6251·4)
Percentage	7·7% (2·5 to 16·6)	1·7% (0·3 to 4·7)	4·2% (0·7 to 10·1)	4·4% (0·8 to 10·3)	21·9% (7·8 to 42·7)	7·6% (2·4 to 16·1)	9·0% (2·1 to 22·8)	9·3% (2·5 to 20·7)	10·8% (3·5 to 22·7)	9·2% (3·2 to 19·1)	29·3% (8·7 to 64·8)
**Rotaviral enteritis**											
Number	146 480·1 (118 037·0 to 183 451·1)	21 357·6 (13 150·8 to 33 967·0)	46 188·4 (32 194·8 to 64 903·3)	5704·8 (3731·6 to 8203·9)	5851·8 (3790·5 to 8370·1)	12 089·5 (7172·8 to 19 007·5)	4194·5 (2360·3 to 7250·0)	2587·9 (1439·9 to 4212·0)	3055·8 (1652·1 to 5141·9)	1742·7 (1132·0 to 2654·7)	2719·3 (1466·4 to 3992·1)
Percentage	29·3% (24·6 to 35·9)	20·4% (13·0 to 32·0)	45·0% (38·5 to 51·7)	17·0% (14·1 to 20·2)	29·5% (25·6 to 33·8)	63·2% (58·4 to 68·1)	26·6% (17·4 to 41·0)	17·7% (14·6 to 21·1)	33·4% (21·5 to 52·9)	19·6% (13·5 to 29·1)	31·8% (27·7 to 36·3)
**Shigellosis**											
Number	54 905·5 (27 026·9 to 94 731·4)	11 597·7 (5522·6 to 20 088·7)	6254·1 (2363·2 to 12 181·5)	7087·3 (3338·5 to 12 739·5)	1187·2 (360·1 to 2594·6)	1225·8 (459·4 to 2409·8)	1459·4 (459·2 to 3387·5)	2962·1 (1116·2 to 5935·6)	2429·6 (1131·5 to 4689·0)	1394·1 (665·0 to 2405·8)	1128·4 (420·8 to 2427·6)
Percentage	11·0% (5·5 to 18·7)	11·1% (5·5 to 18·4)	6·1% (2·6 to 11·6)	21·1% (11·0 to 35·9)	6·0% (2·0 to 12·1)	6·5% (2·9 to 11·1)	9·2% (3·2 to 20·2)	20·2% (9·4 to 35·3)	26·5% (14·0 to 43·9)	15·6% (8·0 to 25·3)	13·2% (5·6 to 24·5)

Data are presented globally and for the ten countries with the highest-burden of diarrhoeal deaths, taken from GBD 2015 for both sexes.

Rotavirus was the leading cause of diarrhoea mortality in children younger than 5 years in most countries; among the ten countries with the highest diarrhoea mortality burden, only Pakistan and Ethiopia had leading causes of of diarrhoeal death that were not rotavirus (table 2). Between 2005 and 2015, rotavirus deaths among children younger than 5 years decreased by 44% (95% UI 33·0–52·0), representing the only aetiology for which the attributable fraction significantly decreased among children under-5 during this time. Rotavirus was also an important cause of diarrhoeal death at older ages—nearly 23% of rotavirus deaths occurred in people older than 5 years old (52 697 deaths, 47 400–57 700) and it was responsible for 199 000 deaths among all ages (165 000–241 000). *Cryptosporidium* spp were one of the leading causes in most of sub-Saharan Africa, but almost exclusively among children younger than 5 years; 93% of the 64 818 *Cryptosporidium* spp deaths occurred in children younger than 5 years. *Shigella* spp were notably different from rotavirus and *Cryptosporidium* spp in that only a third of their 164 300 deaths (95% UI 85 000–278 700) were in children younger than 5 years.

*C difficile* was the main aetiology of diarrhoeal death in high-income countries at all ages (figure 5) and was the only aetiology that increased in attributable fraction between 2005 and 2015 (39·8% increase, 95% UI 29·6–49·9), particularly among adults aged 70 years or older (60·8% increase, 49·0–71). Cholera mortality patterns have distinct geographic variation with the highest attributable fractions in sub-Saharan Africa and southeast Asia (figure 5).

**Figure 6:**
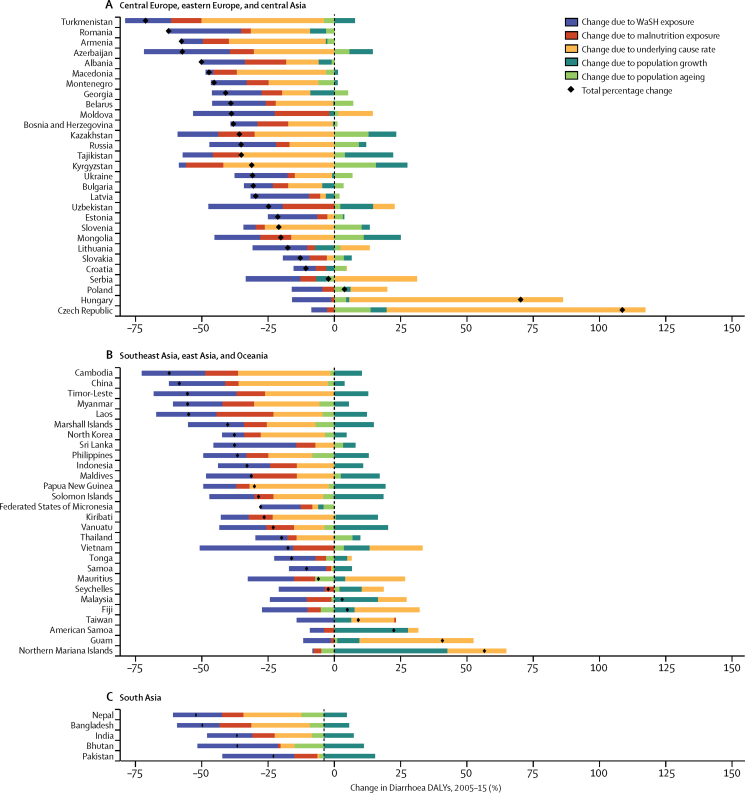
Risk factor and cause decomposition of changes in attributable DALYs among all ages in central Europe, eastern Europe, and central Asia, southeast Asia, east Asia, and Oceania, and South Asia, 2005–15 Changes from 2005 to 2015 are shown for (A) central Europe, eastern Europe, and central Asia, (B) southeast Asia, east Asia, and Oceania, and (C) South Asia. Black dots represent the overall rate of change in DALYs attributable to each risk or cause. Colours represent the population and cause–rate contribution to the rate of change. Bars to the left of zero show a reduction in attribution and bars to the right show an increase. Red bars show the change in risk factor or cause attribution after accounting for the other factors. DALYs=disability-adjusted life-years.

**Figure 7:**
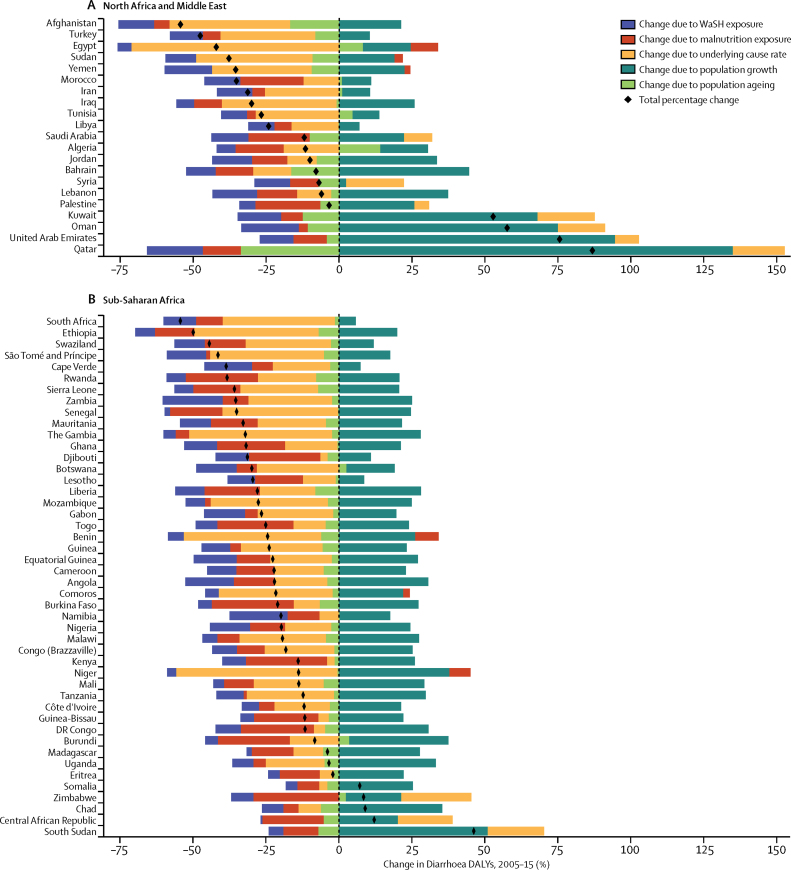
Risk factor and cause decomposition of changes in attributable DALYs among all ages in north Africa and the Middle East and sub-Saharan Africa, 2005–15 Changes from 2005 to 2015 are shown for (A) north Africa and the Middle East and (B) sub-Saharan Africa. Black dots represent the overall rate of change in DALYs attributable to each risk or cause. Colours represent the population and cause–rate contribution to the rate of change. Bars to the left of zero show a reduction in attribution and bars to the right show an increase. Red bars show the change in risk factor or cause attribution after accounting for the other factors. DALYs=disability-adjusted life-years.

**Figure 8:**
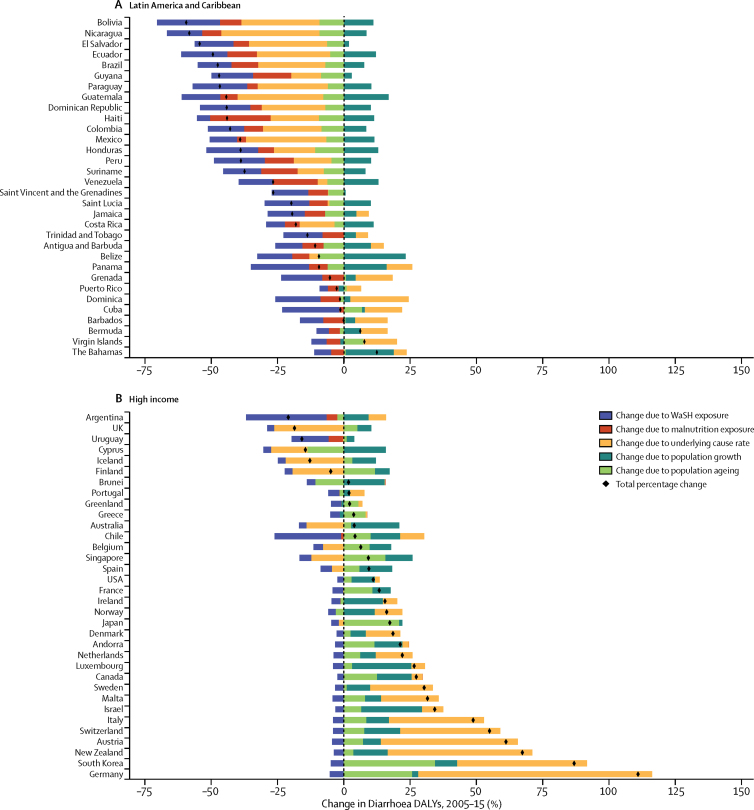
Risk factor and cause decomposition of changes in attributable DALYs among all ages in Latin America and Caribbean and high-income countries, 2005–15 Changes from 2005 to 2015 are shown for (A) Latin America and Caribbean and (B) high-income countries. Black dots represent the overall rate of change in DALYs attributable to each risk or cause. Colours represent the population and cause–rate contribution to the rate of change. Bars to the left of zero show a reduction in attribution and bars to the right show an increase. Red bars show the change in risk factor or cause attribution after accounting for the other factors. DALYs=disability-adjusted life-years.

The leading risk factors for diarrhoea were unchanged from 2005 to 2015. In 2015, unsafe water was responsible for 61·1 million DALYs (95% UI 49·4 million to 69·6 million; 85·4% of diarrhoeal DALYs) and unsafe sanitation was responsible for 40·0 million DALYs (36·0 million to 44·4 million). Among children younger than 5 years, wasting was the leading risk factor for DALYs due to diarrhoea, responsible for 86·3% (72·3–91·4; 38·9 million DALYs, 31·8–44·3) of the 45·1 million diarrhoea DALYs (40·7–50·1). Other risk factors for children were also responsible for under-5 DALYs, such as suboptimal breastfeeding (35·7%, 24·6–46·75), vitamin A deficiency (12·9%, 7·3–18·6), and zinc deficiency (6·5%, 0·6–13·8). The number of DALYs due to diarrhoea decreased for most countries between 2005 and 2015 (figure 6, figure 7, and figure 8 and table 1). Reductions in childhood undernutrition prevalence and improvements in safe water, sanitation, and hygiene (WaSH) have appreciably contributed to reductions in diarrhoea DALYs in many countries (figure 6–8). At the global level, between 2005 and 2015, diarrhoea DALYs attributable to unsafe water and poor sanitation have decreased by 13·4% and those attributable to childhood undernutrition have decreased 10·0% during this time.

Diarrhoea DALYs attributable to unsafe WaSH decreased in all countries, with the greatest reduction in Vietnam (35·2%; figure 6B), but sub-Saharan Africa lagged in WaSH-related diarrhoea DALYs, where the global minimum change occurred in the Central African Republic (0·6%; figure 7B). Outside high-income regions, the smallest reductions in DALYs attributable to WaSH were in eastern sub-Saharan Africa (7·2% reduction) and the Caribbean (7·2% reduction). The largest regional reductions in DALYs attributable to WaSH occurred in South America: the decrease was 28·3% in southern Latin America and 20·8% in Andean Latin America. DALYs due to diarrhoea attributable to childhood undernutrition decreased in most countries, ranging from a 29·3% decrease in Zimbabwe to a 9·3% increase in Egypt, with substantial reductions in DALYs due to undernutrition-associated diarrhoea in many countries of sub-Saharan Africa, including the Democratic Republic of the Congo, Kenya, and Burkina Faso (figure 7B). The largest regional reductions in DALYs due to childhood undernutrition were in central sub-Saharan Africa (21·7% reduction) and the Caribbean (19·7% reduction).

Our results can be explored in further detail online with the Institute for Health Metrics and Evaluation's GBD Compare visualisation platform.

## Discussion

GBD 2015 provides the most comprehensive assessment of the global burden of diarrhoeal diseases to date. The results show that deaths due to diarrhoea among children younger than 5 years decreased by 34·3% between 2005 and 2015 and decreased by 20·8% among people of all ages. Despite substantial reductions, diarrhoea remains an important preventable burden of disease, particularly in south Asia and sub-Saharan Africa. With immediate and sustained actions to decrease both the incidence and mortality attributed to diarrhoea, including appropriate case management, the burden of this prominent public health threat could still be further substantially reduced.^23^, ^24^

Rotavirus is the most common cause of mortality due to diarrhoea. Between 2005 and 2015, under-5 mortality due to rotavirus decreased by 43·6%, faster than the decrease in all-diarrhoea mortality. This decrease is probably due in large part to the introduction of rotavirus vaccine and the scale-up of vaccination related to support from Gavi, the Vaccine Alliance. With Gavi support by the end of 2015, 37 countries had introduced the vaccine, but only about 20% of under-5 children in Gavi-eligible countries have received the rotavirus vaccine.^25^ As of March, 2017, 91 countries have introduced the rotavirus vaccine.^26^

Our results suggest that development of additional vaccines might be warranted. *Cryptosporidium* spp were the second most common cause of diarrhoea deaths among children younger than 5 years. Few therapeutic options for *Cryptosporidium* spp exist^27^ and there are no vaccine candidates, an apparent gap in treatment and prevention. Several candidate combination vaccines against ETEC and *Shigella* spp are in development^28^ and such a vaccine might prevent a large burden of diarrhoeal disease, including in older children and adults given that nearly two-thirds of deaths due to *Shigella* spp occurred in adults and children older than 5 years.

The reduction in DALYs due to diarrhoea is largely attributable to reductions in mortality and can probably be traced to improvements in treatment and prevention, such as reductions in childhood undernutrition prevalence and expanded access to safe water and sanitation.^24^, ^29^ Our results suggest that large strides in reducing childhood undernutrition, especially in tropical Latin America and some countries in sub-Saharan Africa, as well as increasing access to safe water and sanitation such as in south and southeast Asia, have contributed to substantial reductions in diarrhoeal DALYs. Reducing exposure to these risk factors was a key focus of the Millennium Development Goals and is included in the SDGs (Figure 6, Figure 7, Figure 8).

In GBD 2015, water and sanitation are modelled as polytomous risk factors (eg, piped, chlorinated) compared with a dichotomous variable, such as improved or unimproved water and sanitation sources as defined by the Joint Monitoring Programme.^30^ Despite large improvements in safe sanitation, our results suggest that use of safe water has increased only slightly.^20^ Interventions that focus on provision of improved water sources without regard for the transport and treatment of the water are less effective than are infrastructural improvements in water provision, such as piped and chlorinated systems.^20^

Childhood undernutrition is a risk factor for infectious diseases other than diarrhoea, including lower respiratory infections and measles.^31^ The reduction in childhood undernutrition is therefore crucial to decreasing under-5 mortality, and direct interventions, such as improved agriculture and supplementary nutritional programmes, and indirect interventions, such as encouraging lower fertility rates and expanded maternal education, are rightly emphasised in SDG 2.^32^, ^33^

Althouh diarrhoea-associated mortality decreased substantially between 2005 and 2015, the morbidity associated with diarrhoea has not decreased nearly as fast, suggesting that much of this reduction might be attributable to appropriate case management including access to health care and the use of oral rehydration solution.^34^ The effectiveness of oral rehydration solution in the prevention of severe dehydration and death further emphasises the fact that diarrhoea-attributable mortality is largely preventable, even in low-resource settings, with appropriate treatment.

The GBD 2015 estimates of diarrhoea mortality in children younger than 5 years in 2015 (498 900, 95% UI 447 500–557 600) are slightlty lower than those produced by the WHO Department of Evidence, Information and Research and the Maternal and Child Epidemiology Estimation (MCEE) group (526 000; appendix p 35).^35^ The total envelope for under-5 mortality was nearly 1·5 million fewer deaths in GBD 2015 compared with the MCEE group estimates. A comparison of aetioloies for diarrhoea-attributable mortality among children younger than 5 years between the Child Health Epidemiology Research Group (CHERG), from which the MCEE developed,^36^ and GBD 2015 estimates for the year 2010, is shown in the appendix (p 34).

There are several reasons to use counterfactual analyses for the attribution of diarrhoea aetiologies. First, multiple pathogens can be present in a single case of diarrhoea, and these pathogens might interact with each other, making it difficult to attribute each case of diarrhoea to one pathogen.^11^, ^13^ Second, the presence of a given pathogen might not be directly related to diarrhoea. For example, the same pathogens might exist in stool from a healthy individual and from someone with diarrhoea, so simply measuring the presence of a given pathogen might not accurately describe diarrhoea burden.^11^ By engaging in scientific debate and learning from the categorical attribution approach used in previous iterations of GBD (2010),^37^ we decided to use a counterfactual approach in future work. Unlike categorical attribution that assigns one outcome to one aetiology, counterfactual analyses allow for multicausality of diarrhoea episodes.

### Comparison with GBD 2013

The GBD 2015 estimates of diarrhoea burden differ from those of previous GBD iterations, including cause attribution. Global under-5 deaths due to diarrhoea in 2010, the most recent shared estimation year, are generally lower in GBD 2015 compared with GBD 2013 estimates. These differences can be traced to three high-population and high-burden countries: Pakistan, Nigeria, and India (appendix).^15^ GBD estimates in India are now made at the subnational level, which has added data and geographic resolution to this country. This modelling change in India has also reduced the non-fatal diarrhoea estimates in the country (appendix).

### Diagnostics

For GBD 2015, we have updated our case definitions for the diarrhoeal aetiologies to reflect detection using molecular methods. This advancement of molecular diagnostic tools enables identification of pathogens that may have previously gone undetected and can more accurately determine the prevalence of pathogens.^13^, ^38^ These diagnostics are more sensitive in pathogen detection than traditional laboratory methods, particularly for bacterial organisms,^13^ and could allow for improvements in case management, epidemiological tracking, and assessing the effectiveness of interventions, such as vaccines. To adopt a molecular diagnostic case definition for our diarrhoeal aetiologies, we introduced a source of uncertainty in our estimates because of the necessity of adjusting our estimates of the proportion of diarrhoea episodes that test positive for each aetiology, according to the non-molecular diagnostic methods, for misclassification of exposure.^39^

### Data limitations

Our estimates of diarrhoea mortality, morbidity, and cause attribution are limited by data availability, especially the sparsity of data in sub-Saharan Africa, the region of the world with the highest diarrhoea burden. It is difficult to assess a systematic bias in morbidity or mortality estimates caused by data gaps because it is not clear that missing data in some countries means that deaths due to diarrhoea are disproportionally higher or lower compared with other preventable causes. We account for confounding effects of diarrhoea and other causes by making use of regional information to inform the fraction of all-cause mortality attributable to diarrhoea. Data sparsity is also reflected in the uncertainty interval for the particular geography (table 1). A list of all GBD 2015 data sources is available for each country online. The MAL-ED study will be a great resource in elucidating the burden of community diarrhoea and its aetiologies, especially in Africa and Latin America.^40^, ^41^ There is also a general dearth of data on diarrhoea in populations older than 5 years, and although we model diarrhoeal aetiologies in these age groups, the OR from the oldest age group in GEMS, which is still younger than 5 years (2–5 years), are assumed to be representative in older ages.^42^ Moreover, our statistical models have a limitation in predicting cases based on very small numbers or when data are absent.

ETEC estimates in GBD 2015 represent the combined burden of the ST and LT genotypes, of which ST is recognised as more frequently associated with diarrhoea.^43^ Although the OR of diarrhoea given detection would be higher for ST if the genotypes were to be differentiated, there would be a tradeoff in the proportion of diarrhoea episodes that test positive for ST-ETEC. The modelling strategy for cholera attribution is limited by case reporting to WHO. Although cholera is a notifiable disease to WHO, many countries underreport or fail to report at all for various social and economic reasons.^18^

### Next steps

Malnutrition or regular illness during the first few years of life has negative effects on future cognitive development, education, and productivity. Despite being the fourth most common cause of DALYs in children younger than 5 years globally, the full burden of non-fatal diarrhoea might remain unknown.^44^, ^45^ Results from many studies have implicated diarrhoea as a risk factor for malnutrition and impaired physical growth, while others have suggested that diarrhoea, possibly mediated by malnutrition, might also impair cognitive development.^46^, ^47^, ^48^ Capturing these sequelae by cause will increase the quantified burden of diarrhoea to more completely measure its effects on child health and potential. Future iterations of GBD will incorporate geospatial data on diarrhoeal burden to map the spatiotemporal distribution of diarrhoea and its aetiologies on a 5 km by 5 km geographic scale, as has been done for malaria.^49^ This work will provide important insight into higher spatial resolution space-time trends in diarrhoea.

### Conclusion

Despite substantial reductions in diarrhoea mortality in many countries, the burden of this preventable disease remains concentrated in the poorest children. Understanding the contribution of each cause to the burden of diarrhoea and how this varies geographically will enable interventions to be targeted. Vaccine use and a continued focus on improving access to WaSH indicators, reducing childhood undernutrition, and providing appropriate treatment and case management will accelerate reductions in diarrhoea disease burden.

Correspondence to: Dr Ali H Mokdad, Institute for Health Metrics and Evaluation, University of Washington, Seattle, WA 98121, USA **mokdaa@uw.edu**

**This online publication has been corrected. The corrected version first appeared at thelancet.com/infection on June 14, 2017**
