# Corrections

**DOI:** 10.1016/S1473-3099(17)30391-2

**Published:** 2017-09

*GBD Diarrhoeal Diseases Collaborators. Estimates of global, regional, and national morbidity, mortality, and aetiologies of diarrhoeal diseases: a systematic analysis for the Global Burden of Disease Study 2015.* Lancet Infect Dis *2017; published online June 1. http://dx.doi.org/10.1016/S1473-3099(17)30276-1*—In this Article, the equation to correct the proportion estimates for exposure misclassification due to diagnostic error has been corrected in the fourth paragraph of the Aetiology section and on appendix p 14. These corrections have been made to the online version as of June 14, 2017.

