# Supplementary material for: Estimates of global, regional, and national morbidity, mortality, and aetiologies of diarrhoeal diseases: a systematic analysis for the Global Burden of Disease Study 2015
Source: Lancet Infect Dis. 2017 Sep;17(9):909–48. doi: 10.1016/S1473-3099(17)30276-1 (PMC5589208; doi:10.1016/S1473-3099(17)30276-1)
Supplement: Supplementary appendix [file mmc1.pdf]

THE LANCET
Infectious Diseases

Supplementary appendix

This appendix formed part of the original submission and has been peer reviewed. We post it as supplied by the authors.

This online publication has been corrected. The corrected version first appeared at thelancet.com/infection on June 14, 2017.

Supplement to: GBD Diarrhoeal Diseases Collaborators. Estimates of global, regional, and national morbidity, mortality, and aetiologies of diarrhoeal diseases: a systematic analysis for the Global Burden of Disease Study 2015. *Lancet Infect Dis* 2017; published online June 1. http://dx.doi.org/10.1016/S1473-3099(17)30276-1.

Appendix to The global burden of diarrhoeal diseases: results from the Global Burden of Disease (GBD) study 2015

This appendix provides methodological detail, supplemental figures, and comprehensive information on input data and data transformation.

Contents

Detail on mortality modeling 2
Details on morbidity modeling 7
Details on aetiology attribution 11
Comparison to GBD 2013 27
Comparison to WHO-MCEE 33
References 36

Summary of diarrhoea mortality modeling

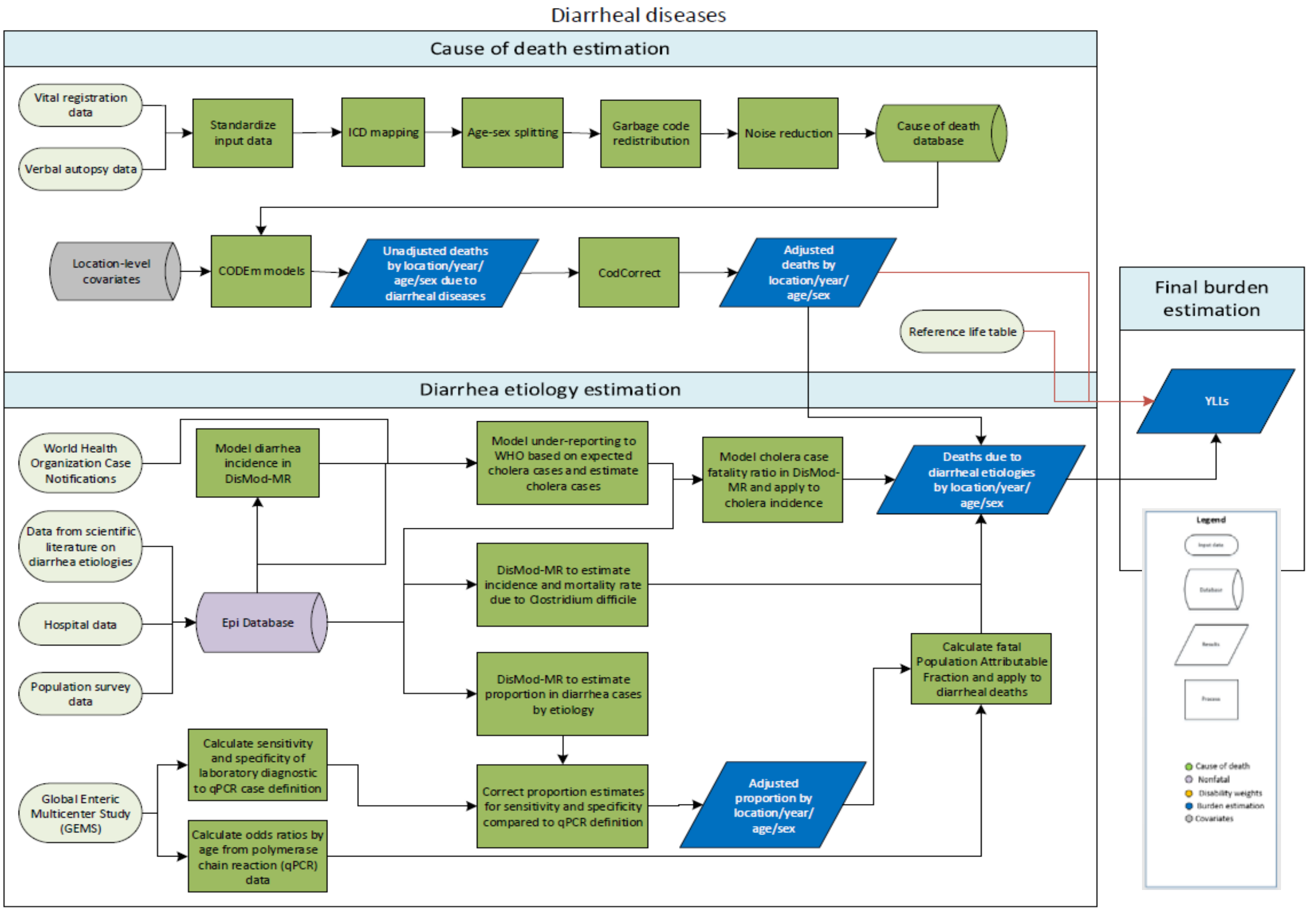

Appendix Figure 1. Analytic flowchart for diarrhoeal mortality estimation, including aetiologic attribution.

The Cause of Death database for the Global Burden of Disease study has a combination of public and private data from surveillance systems, vital registration systems, and verbal autopsy. To build the database, we first identified verbal autopsy studies, irrespective of cause, by searching PubMed and Google Scholar for all studies with the term "verbal autopsy", and did country-specific searches on Google using the country name and "verbal autopsy". We also identified studies from systematic reviews of diarrhoea mortality and updated them by searching for "verbal autopsy child diarrhoea" and "verbal autopsy child diarrhoea" in Google Scholar. We included studies that used VA, had over 50 deaths, provided the number of deaths due to diarrhoea, and were conducted for at least one year to avoid seasonality. A summary of the input data is shown in **Appendix Figure 2.** Diarrhoea mortality was defined by ICD9 and ICD10 codes (ICD9 codes 001-001.9, 003-006.9, 007.4-007.8, 008.01-008.02, 008.04, 008.2-

009.9, and 787.91; ICD10 codes: A00-A00.9, A02-A04.1, A04.3, A04.5-A07, A07.2-A07.4, A08-A09.9, and R19.7).

A key component of cause of death modeling in GBD is the redistribution of poorly coded causes of death such as “infection”, “fever”, or “dehydration” to specific causes of death.[1] This processing of *garbage* codes, causes of death that cannot or should not be considered underlying causes of death, reallocates a number of deaths into diarrhoeal diseases. The garbage code redistribution was informed by an IHME expert review of the data and subsequent modeling.[1]

For published studies where age groups did not match the GBD age groups, we performed an age-sex split based on the global age distribution of diarrhoea mortality. GBD age groups include: 0-6 days, 7-27 days, 28-364 days, 1-5 years, then in 5-year age groups to age 80+. Where necessary, the overall mortality envelope and population estimates by age, sex, and location were used to calculate cause fraction and mortality rates for each data point.

There were 510,000 data points on diarrhoea mortality that were used in the modeling. We also excluded early neonatal mortality data in the Philippines (1994–1998) and India Civil Registration System data for many states (1986–1995). Overall, 14,622 data points were excluded or outliered (2.8% of data points).

Diarrhoeal disease mortality was estimated in the Cause of Death Ensemble model (CODEm) platform.[2,3] CODEm is a Bayesian statistical model and uses spatial priors from a hierarchical structure to inform the mortality models. CODEm is based on five general principles: identifying all available data, maximizing the comparability and quality of the dataset, developing a diverse set of plausible models, assessing the predictive validity of each plausible individual model and of ensemble models, and choosing the model or ensemble model with the best performance in out-of-sample predictive analysis. CODEm produces a large suite of models based on either cause fraction or mortality rate, uses linear and space-time Gaussian process regression (ST-GPR), and a covariate selection process. Each sub-model is evaluated using out-of-sample predictive validity. Thirty percent of the data are excluded from the initial model fits and 15% are used to evaluate component models and 15% used to build the ensembles. The sub-models are ranked using 15% of the data based on their out-of-sample predictive validity. The proportion weighting of the ensemble sub-models is evaluated using the remaining 15% of the hold-out data. This weighting scheme evaluates ensemble models that are built with ranked sub-models contributing proportionally more or fewer draws to the final ensemble. The final ensemble model is evaluated against other ensemble models using the same fit statistics (in-sample, out-of-sample root mean squared error and data coverage). Detailed information on this process can be found in Foreman et al 2012[4] and in the GBD 2015 Mortality and Causes of Death manuscript.[5]

Covariates are selected independently for each sub-model and the selection is based on an algorithm that captures plausible relationships between the covariates and diarrhoeal

mortality and provides a diversity of plausible models (**Appendix Table 1**). For every covariate, the direction of effect and a level of biologic proximity to diarrhoea mortality was defined. Each model includes all combination of covariates if the direction of effect is along the assumed direction and the coefficient is significant at $p < 0.05$ level. Also, if adding a more distal covariate changes the statistical significance of a more proximal covariate to non-significant or changes the direction of effect, it will be dropped from the set. The reason for this algorithm to give priority and emphasis on covariates that are more causally and proximately related to diarrhoea such as unsafe water, unsafe sanitation and malnutrition rather than more contextual and macro covariates such as education and income per capita.

Diarrhoea mortality is estimated for 21 age groups, 591 locations, both sexes, and every year from 1980-2015. We estimated diarrhoea mortality separately for males and females and for children under 5 years and older than 5 years. Data-rich and data-poor geographic locations were modeled separately and these models were then hybridized for a global model.

Like all models of mortality in GBD, diarrhoea mortality models are single-cause, requiring in effect that the sum of all mortality models must be equal to the all-cause mortality envelope. We correct diarrhoea mortality, and other causes of mortality, by re-scaling them according to the uncertainty around the cause-specific mortality rate. This process is called CoDCorrect and is essential to ensure internal consistency among causes of death.

Appendix Table 1. Covariates in CODEm. CODEm uses a covariate selection algorithm and chooses from the covariates listed in the table below. Covariates are selected from this list while considering prior information about the strength of the association and direction of effect between the covariate and diarrhoeal mortality. The strength of the relationship is ranked from 1 (in causal pathway) to 3 (likely related to diarrhoea mortality). The direction of the relationship is indicated by +, -, and 0 where 0 indicates that a covariate can have either direction of effect in the model.

Covariate	***Strength of Relationship***	***Direction***
*Diarrhoea Summary Exposure Variable (SEV)**	1	+
Rotavirus vaccine	1	-
Safe sanitation	1	-
Safe Water	1	-
*Sanitation SEV**	1	+
*Water SEV**	1	+
Malnutrition <2 SD	2	+
Education per Capita	3	-
LDI per Capita	3	-
Population <150/km2	3	0
Population >1000/km2	3	0
Population -15:15 Latitude	3	+
Socio-demographic status	3	-

*Summary exposure variables are a risk-weighted prevalence of exposure, scaled so that 1 is 100% of the population exposed and 0 is 0%.[6]

Appendix Figure 2. Diarrhoea mortality input data geographic distribution. The number of verbal autopsy or vital registration data points for all ages and from 1980-2015 are shown. Countries in white have no data. Subnational estimation occurs in the United States, Mexico, Brazil, South Africa, the United Kingdom, Saudi Arabia, India, China, and Japan.

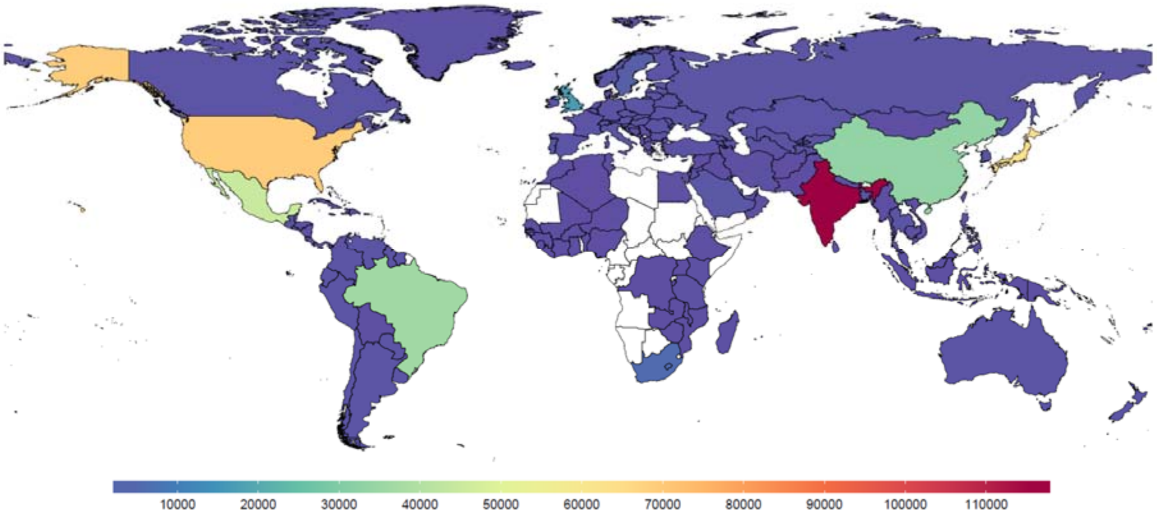

Summary of diarrhoea morbidity modeling

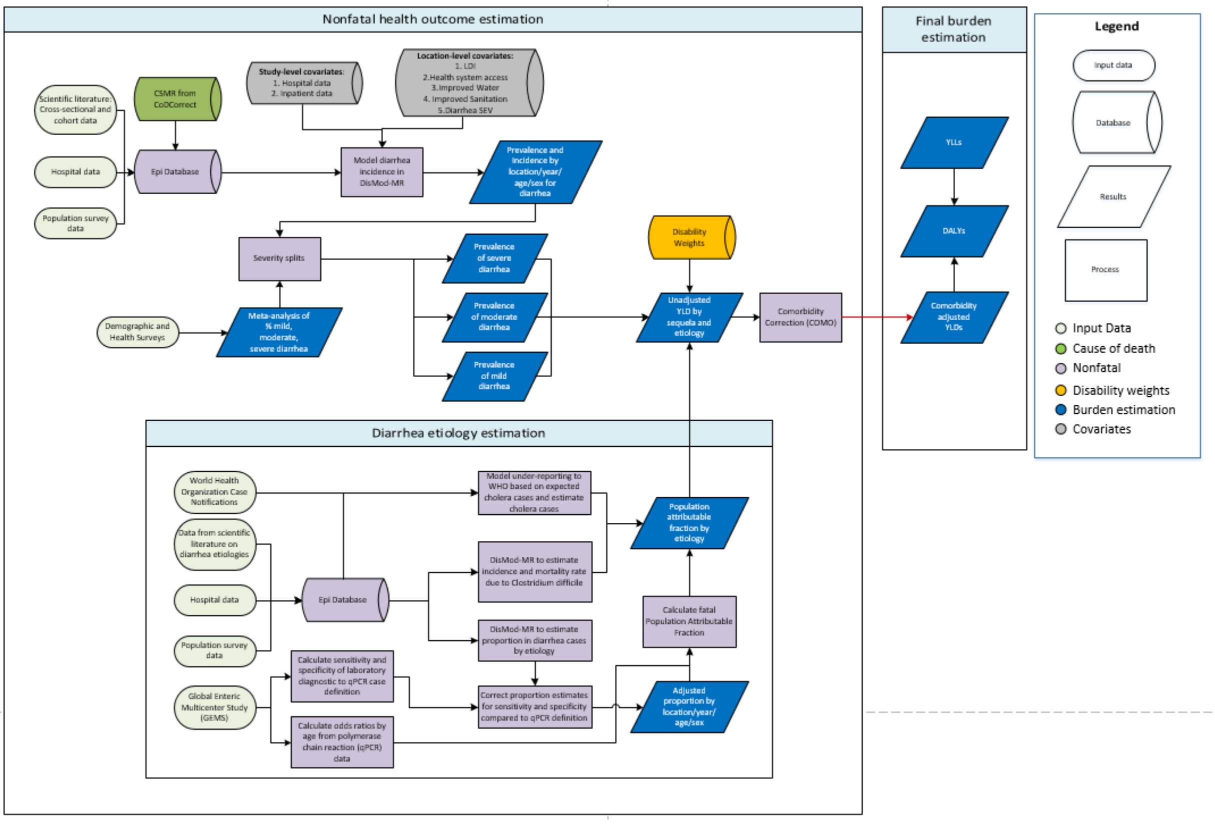

Appendix Figure 3. Analytic flowchart for diarrhoeal morbidity estimation strategy including aetiologic attribution.

Diarrhoea morbidity was modeled in the DisMod-MR 2.1 platform.[7] DisMod is a Bayesian, hierarchical, meta-regression tool that relates incidence, prevalence, recovery, and mortality in a compartmental model of disease progression. We set the average duration of illness at 4.2 days in the model. Input data are from population representative surveys such as the Demographic and Health Survey (DHS), hospital inpatient and outpatient data (ICD9 codes 001-009.9 and ICD10 codes A00-A09), and from the scientific literature. Input data include all data used in GBD 2013 and a new review of data sources from 2012-August 2015. A summary of the input data is provided in **Appendix Figure 4** and the PubMed search string is also listed (**Search strings**). Diarrhoea incidence and prevalence data were extracted concurrently with the aetiology proportion data described on Appendix pages 16-19.

Diarrhoeal disease episodes are characterized as three or more loose stools in a 24 hour period. The reference category for our input data is community based diarrhoea episodes such as data from population-representative surveys or community cohorts. Input data that are from a different population, such as hospital outpatient or inpatient groups, are adjusted by study-

level covariates so that they are consistent with the reference category. This step occurs in DisMod.

Data from population-representative surveys, such as the Demographic and Health Surveys and the Multiple Indicator Cluster Surveys, were used and identified using the Global Health Database (GHDx: www.ghdx.healthdata.org). DisMod prevalence input data must be point prevalence. Maternal reported 2-week period prevalence from the surveys was converted to point prevalence in 1-year age increments. Period prevalence was converted to point prevalence using the following formula:

$$p_{point} = p_{period} * \frac{d}{d - 1 + r}$$

Where *d* is duration in days and *r* is the recall period in days.

Hospital data and healthcare utilization (MarketScan, United States only) data were identified using the ICD9 codes 001-009.9 and ICD10 codes A00-A09. To be consistent with the survey data, we transformed the hospital and MarketScan data from incidence to prevalence using an average duration of 4.2 days. Mortality rates from the final Cause of Death model are also used in DisMod as excess mortality rates, a ratio of mortality to prevalence.

Country-level covariates also inform the model. These include the proportion of the population that have access to improved sanitation, access to improved water sources, health system access, income per capita, and the summary exposure variable (SEV) for diarrhoea (**Appendix Table 2**). The diarrhoea SEV is the sum of risk-weighted prevalence of exposure for each of the risk factors associated with diarrhoea.[6] The risk factors for diarrhoea in the GBD are unsafe water and sanitation, no handwashing with soap, childhood malnutrition, vitamin and zinc deficiency, and sub-optimal breastfeeding.

Diarrhoeal diseases have three severity levels: mild, moderate, and severe (**Appendix Table 3**). The proportion of diarrhoea cases that are assigned to each comes from an analysis of Demographic and Health Surveys. Mild cases are the proportion of diarrhoea cases that did not seek medical care; moderate cases are the proportion that sought medical care but did not have severe dehydration or seizures; and severe cases are the proportion that sought medical care with severe dehydration or seizures.[8]

To estimate years lived with disability (YLDs) from diarrhoeal diseases, we applied disability weight estimates for each of the possible disease states for prevalent cases of diarrhoeal diseases and the percent of prevalent cases that fall into each state. In the case of diarrhoeal diseases, we assumed that there were three mutually exclusive disease states – mild, moderate, and severe – with corresponding disability weights of 0.074, 0.188, and 0.247, respectively. Disability weights are values between 0 and 1 where 0 represents perfect health and 1 represents death. The disability weights were separately estimated in the Disability Weights Survey portion of the GBD study and were systematically constructed based on responses from more than 6,000 survey respondents.[9]

Appendix Figure 4. Geographic distribution of diarrhoea morbidity modeling. The number of data points by country is shown. Overall, there are 24,463 data points from 639 unique sources.

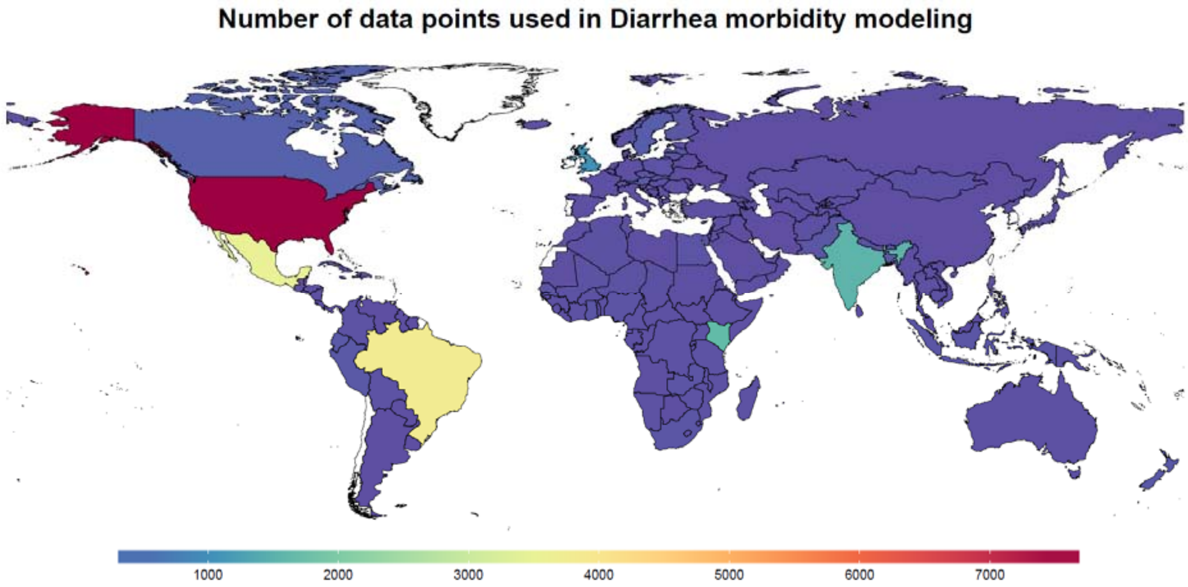

Appendix Table 2. Summary of covariates used in the diarrhoea DisMod-MR meta-regression model. Study-level covariates are binary indicators that are used in DisMod to make data directly comparable from disparate sources. Hospital data are systematically lower than the referent category and so are adjusted upward in the modelling process. Country-level covariates are representative of the country-years used in the model and inform the prediction in areas without data. Numbers in parentheses are 95% uncertainty intervals.

Covariate name	Type of covariate	Parameter	Beta coefficient
Hospital data	Study	Prevalence	0.40 (0.38-0.43)
Hospital inpatient population	Study	Prevalence	0.44 (0.42-0.47)
Hospital data from middle- or low-income country	Study	Prevalence	0.06 (0.05-0.06)
Improved sanitation	Country	Prevalence	1.52 (1.35-1.68)
Improved water source	Country	Prevalence	0.05 (0.04-0.06)
Diarrhoea SEV	Country	Prevalence	1.03 (1.0-1.06)
Health system access	Country	Excess mortality	0.49 (0.48-0.50)
Income per capita	Country	Excess mortality	2.13 (2.09-2.18)

Appendix Table 3. Details on the severity levels for diarrhoea in GBD 2015 and the associated disability weight (DW) with that severity. Numbers in parentheses are 95% uncertainty intervals.

Severity level	Lay description	Disability Weight (95% CI)	Percent of Cases (95% CI)
Mild	Has diarrhoea defined as 3 or more loose stools in a 24 hour period with no dehydration	0.074 (0.049-0.104)	24.3% (23.2-25.3%)
Moderate	Has diarrhoea defined as 3 or more loose stools in a 24 hour period and sought medical treatment without dehydration	0.188 (0.125-0.264)	61.7% (60.5-62.8%)
Severe	Has diarrhoea defined as 3 or more loose stools in a 24 hour period and sought medical treatment with dehydration	0.247 (0.164-0.348)	14.0% (13.1-15%)

Summary of aetiology population attributable fraction strategy

We estimated diarrhoeal disease aetiologies separately from overall diarrhoea mortality and morbidity using a counterfactual strategy for enteric adenovirus, *Aeromonas*, *Entamoeba histolytica* (amoebiasis), *Campylobacter enteritis*, cryptosporidiosis, typical enteropathogenic *Escherichia coli* (t-EPEC), enterotoxigenic *Escherichia coli* (ETEC), norovirus, non-typhoidal salmonella infections, rotavirus, and *Shigella*. *Vibrio cholerae* and *Clostridium difficile* were modeled separately.

For all aetiologies except *V cholerae* and *C difficile,* the population attributable fraction (PAF) was calculated from the proportion of diarrhoea cases that are positive for each aetiology and the odds ratio of diarrhoea given the detection of that aetiology. This is a counterfactual approach, meaning that the PAF represents the relative reduction in diarrhoea episodes and deaths if there was no exposure to a given aetiology. As diarrhoea can be caused by multiple pathogens and the pathogens may co-infect, PAFs can overlap and add up to more than 100%.

We used the following formula to estimate PAF:[10]

$$PAF = Proportion * (1 - \frac{1}{OR})$$

Where *Proportion* is the proportion of diarrhoea cases positive for an aetiology and *OR* is the odds ratio of diarrhoea given the presence of the pathogen. Both of these values are described in detail below.

Molecular diagnostic methods

For GBD 2015, we used a systematic reanalysis of the Global Enteric Multicenter Study (GEMS) that uses quantitative polymerase chain reaction (qPCR) as the diagnostic tool for pathogen detection to estimate the odds ratios of diarrhoea given pathogen detection. Validation studies have shown that this approach is more sensitive than traditional laboratory diagnostic methods in detecting diarrhoeal pathogens.[11,12]

The qPCR test results are a continuous variable corresponding to the relative quantity of genetic target in the sample. To be consistent with a binary presence/absence of pathogen case definition from the literature review, we dichotomized the continuous qPCR test result using the lowest value of the cycle threshold (Ct) that accurately discriminated between cases and non-cases in GEMS (**Appendix Figure 5A**). We used the lower Ct value that represented the smallest false positive samples (positive in non-diarrhoea samples) when we had multiple Ct values for the cutpoint (**Appendix Figure 5B**). The Ct values range from 0 to 35 cycles representing the relative concentration of the target gene in the stool sample. A low value indicates a higher concentration of the pathogen while a value of 35 indicates the analytic level of detection. Values above 35 are not reproducible due to the stochasticity involved in the physical distribution of the clinical specimen to wells in the array where the singleplex qPCR is

performed. The case definition for each pathogen is a Ct value that is below the established cutoff point.

We used a mixed effects conditional logistic regression model, matching for case-control pairs, random effects for GEMS sites, and accounting for all pathogens to calculate the odds ratio by age for each of our etiologies. This means that an odds ratio by age for each aetiology is applied regardless of the year or geographic location.

Appendix Figure 5. Plotted representation of qPCR cycle threshold (Ct) and the diagnostic discrimination between cases and controls in GEMS (accuracy). A). The relationship between Ct and accuracy is shown for rotavirus, **B).** The relationship between Ct and accuracy is shown for adenovirus. Adenovirus has two inflection points in the smoothed accuracy curve and so the lower of the two points is chosen as the cutoff.

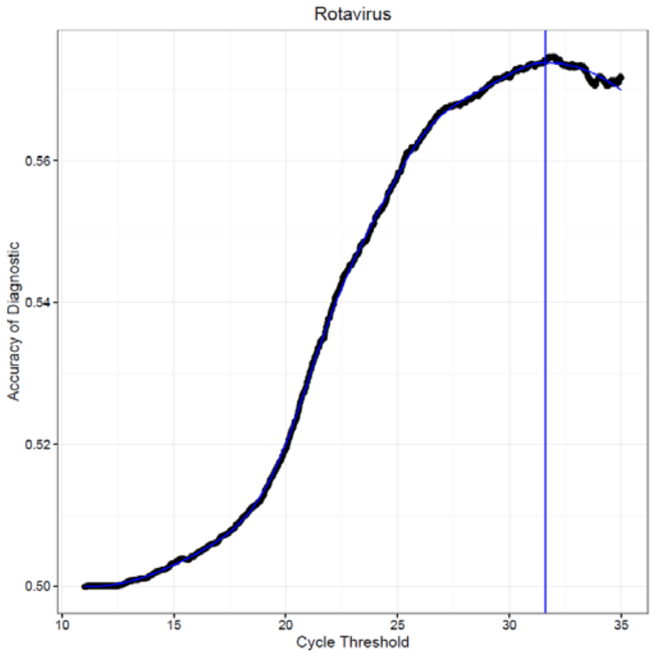

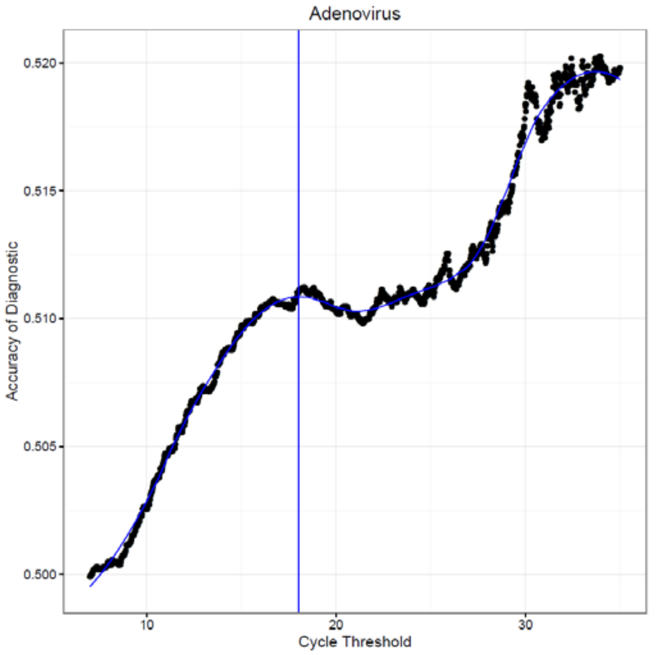

Modeled aetiologic proportion

We modeled the proportion data using the meta-regression tool DisMod-MR to estimate the proportion of positive diarrhoea cases for each separate aetiology by location/year/age/sex and to adjust for covariates. DisMod adjusts data to be comparable before performing meta-regressions. A binary indicator for if the proportion data come from inpatient populations, assumed to be a proxy for severe and fatal diarrhoea episodes, is used as a scalar to differentiate the relative frequency of detection in fatal and non-fatal diarrhoea episodes for distinct PAFs for fatal and non-fatal diarrhoea episodes.

We used the estimated sensitivity and specificity of the laboratory diagnostic technique used in the GEMS study compared to the qPCR case definition among cases to adjust our proportion before we computed the PAF:[13,14]

$$Proportion_{True} = \frac{(Proportion_{Observed} + Specificity - 1)}{(Sensitivity + Specificity - 1)}$$

We used this correction to account for the fact that the proportions we used are based on a new test that is not consistent with the laboratory-based case definition (qPCR versus GEMS conventional laboratory testing for pathogens).[15] A summary of the sensitivity and specificity of the non-molecular diagnostics to the molecular case definition for each pathogen is shown in **Appendix Figure 6.**

Appendix Figure 6. The sensitivity and specificity of the non-molecular diagnostic methods to the molecular-based case definition is shown for each pathogen. The sensitivity and specificity values are from diarrhoea cases in the Global Enteric Multicenter Study. The error bars represent the 95% confidence interval based on random-sampling bootstrap of the individual-level data.

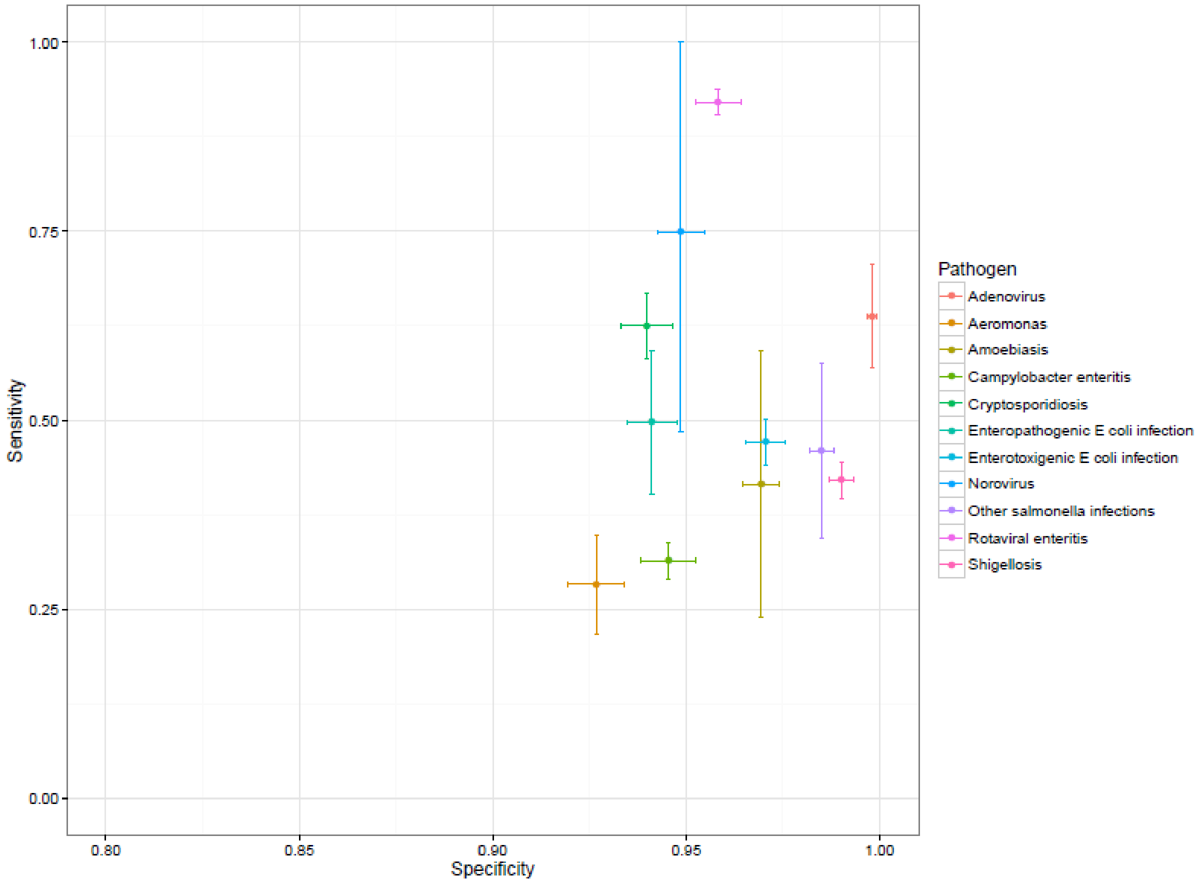

Our literature review extracted the proportion of any enteropathogenic *Escherichia coli* (EPEC) without differentiating between typical (tEPEC) and atypical (aEPEC). In order to be consistent with the odds ratios that we obtained, which described tEPEC, we adjusted our proportion estimates of any EPEC to typical EPEC only. This adjustment was informed by a subset of our literature review that reported both atypical and typical EPEC. We estimated a ratio, by super-region, of tEPEC to any EPEC and adjusted our proportion estimates accordingly. We found that the majority of EPEC diarrhoea cases were positive for atypical EPEC, consistent with other published work.[16]

For *Vibrio cholerae* (cholera), we used the literature review to estimate expected number of cholera cases for each country-year using the incidence of diarrhoea, estimated using DisMod-MR, and the proportion of diarrhoea cases that are positive for cholera. We assigned cholera PAF using odds ratios from the qPCR results to estimate a number of cholera-

attributable cases. We compared this expected number of cholera cases to the number reported to the World Health Organization at the country-year level.[17] We modeled the underreporting fraction to correct the cholera case notification data for all countries using health system access and the diarrhoea SEV scalar to predict total cholera cases. We used the age-specific proportion of positive cholera samples in DisMod and our incidence estimates to predict the number of cholera cases for each age/sex/year/location. Finally, we modeled the case fatality ratio of cholera using DisMod-MR and to estimate the number of cholera deaths.

For *C. difficile*, we modeled incidence and mortality in DisMod-MR for each age, sex, year, location. DisMod-MR is a Bayesian meta-regression tool that uses spatio-temporal information as priors to estimate prevalence, incidence, remission, and mortality for *C. difficile* infection. DisMod-MR uses a compartmental model to relate prevalence, incidence, remission, and mortality. We set remission in our model to 1 month.

Aetiology proportion data

The proportion of diarrhoea episodes where each aetiology is detected is extracted from a systematic literature review. Inclusion criteria are sample population greater than 100 individuals, studies lasting longer than 1-year in duration, and from non-epidemic locations. We excluded studies that reported on diarrhoeal outbreaks and those that used acute gastroenteritis with or without diarrhoea as the case definition. We did not set language restrictions to the search criteria.

For GBD 2015, we updated our review of literature to include studies published between January 2012 and May 2015 (**Appendix Table 4**). The PubMed search strings are provided below. We identified 2,847 studies, of which 152 met our criteria of inclusion and were included. We extracted data points for location, sex, year, and age. The geographic distribution of aetiology data points is shown in **Appendix Figure 7**. We assigned an age range based on the prevalence-weighted mean age of diarrhoea in the appropriate year/sex/location if the age of the study participants was not reported.

We modeled *Vibrio cholerae* independently from the other aetiologies because of its epidemic tendency. We conducted a systematic review of literature for studies published between January 1980 and June 2015 that reported the proportion of diarrhoea cases that tested positive for cholera or the case fatality of cholera (**Search string 2**). We excluded studies specifically about outbreaks and with less than one year of follow-up.

We also modeled *Clostridium difficile* independently from the aetiologies because it was not included as a pathogen in GEMS. We conducted a systematic literature review for the prevalence and incidence of *C. difficile* between January 1990 and May 2015 (**Search string 3**). We used inpatient and outpatient hospital visits coded for *C. difficile* as our incidence data. However, nearly all of the hospital data came from Western countries (**Appendix Figure 7**).

Appendix Table 4. Summary of diarrhoea aetiology data. The number and a description of the data and data types that are used in the diarrhoea aetiologic attribution modeling is shown for each aetiology. Each data source has one or more data point depending on the number of year, age-, and sex-specific values are reported.

Aetiology	Total data points	Data points new to GBD 2015	Unique data sources	Number (percent) of GBD Locations	Number (percent) from Inpatient population	Number (percent) from single pathogen study	Number (percent) from Children Under 5yrs
Adenovirus	331	127 (38.4%)	92	52 (9.3%)	178 (53.8%)	0 (0%)	119 (36%)
Aeromonas	116	14 (12.1%)	41	22 (3.9%)	64 (55.2%)	4 (3.4%)	31 (26.7%)
Amoebiasis	204	57 (27.9%)	70	37 (6.6%)	106 (52%)	11 (5.4%)	63 (30.9%)
Campylobacter enteritis	482	187 (38.8%)	141	60 (10.7%)	230 (47.7%)	77 (16%)	148 (30.7%)
Cholera O1 serogroup	2204	2074 (94.1%)	92	146 (26%)	288 (13.1%)	997 (45.2%)	83 (3.8%)
Clostridium difficile	19219	212 (1.1%)	111	30 (5.3%)	8724 (45.4%)	0 (0%)	2109 (11%)
Cryptosporidiosis	414	204 (49.3%)	89	49 (8.7%)	273 (65.9%)	63 (15.2%)	100 (24.2%)
Enteropathogenic E coli infection	278	86 (30.9%)	96	53 (9.4%)	162 (58.3%)	25 (9%)	96 (34.5%)
Enterotoxigenic E coli infection	419	48 (11.5%)	113	47 (8.4%)	187 (44.6%)	48 (11.5%)	133 (31.7%)
Norovirus	393	178 (45.3%)	80	46 (8.2%)	147 (37.4%)	96 (24.4%)	127 (32.3%)
Other salmonella infections	644	348 (54%)	155	62 (11.1%)	366 (56.8%)	29 (4.5%)	143 (22.2%)
Rotaviral enteritis	2154	652 (30.3%)	399	107 (19.1%)	1329 (61.7%)	1033 (48%)	1003 (46.6%)
Shigellosis	603	171 (28.4%)	165	61 (10.9%)	275 (45.6%)	32 (5.3%)	180 (29.9%)

Diarrhoea literature review search strings. Search terms used in PubMed systematic literature review for GBD 2015. Search string 1 is for diarrhoea and all aetiologies except cholera and *Clostridium* and are an update from 2012-2015. Search strings 2 and 3, cholera and *Clostridium*, are full reviews from 1990 to 2015.

1. ***Diarrhoea and aetiologies:*** *(diarrhoea[title] OR diarrhoea[MeSH Terms] OR diarrhoea[title] OR diarrhoea[MeSH Terms] OR gastroenteritis[title] OR gastroenteritis[MeSH Terms] OR gastro-enteritis[title] OR salmonella[title/abstract] OR shigell*[title/abstract] OR "enteropathogenic e. coli" [title/abstract] OR enterotoxigenic e. coli[title/abstract] OR campylobacter[title/abstract] OR amoebiasis[title/abstract] OR entamoeb*[title/abstract] OR amoebiasis[title/abstract] OR amebiasis[title/abstract] OR cryptosporidi*[title/abstract] OR rotavirus[title/abstract] OR norovirus[title/abstract] OR adenovirus[title/abstract]) AND ((aetiolog*[title/abstract] OR aetiology[MeSH Terms] OR cause[title/abstract] OR pathogen[title/abstract]))* ***NOT*** *((colitis[title/abstract] OR enterocolitis[title/abstract] OR inflammatory bowel[title/abstract] OR irritable[title/abstract]OR Crohn*[title/abstract] OR HIV[title] OR treatment[title] OR therapy[title])) NOT ((appendicitis[title/abstract] OR esophag*[title/abstract] OR surger*[title/abstract] OR gastritis[title/abstract] OR liver[title/abstract] OR case report[title] OR case-report[title] OR therapy[title] OR treatment[title])) AND (("2012/01/01"[PDat] : "2015/12/31"[PDat]) AND Humans[Mesh])*
2. ***Cholera:*** *(((diarrhoea[title] OR diarrhoea[MeSH Terms] OR diarrhoea[title] OR diarrhoea[MeSH Terms] OR gastroenteritis[title] OR gastroenteritis[MeSH Terms] OR gastro-enteritis[title] AND cholera[title/abstract] OR cholera[MeSH Terms]) AND ((aetiolog*[title/abstract] OR aetiology[MeSH Terms] OR cause[title/abstract] OR pathogen[title/abstract])) NOT ((colitis[title/abstract] OR enterocolitis[title/abstract] OR inflammatory bowel[title/abstract] OR irritable[title/abstract]OR Crohn*[title/abstract] OR HIV[title] OR treatment[title] OR therapy[title])) NOT ((appendicitis[title/abstract] OR esophag*[title/abstract] OR surger*[title/abstract] OR gastritis[title/abstract] OR liver[title/abstract] OR case report[title] OR case-report[title] OR therapy[title] OR treatment[title])) AND (("1990/01/01"[PDat] : "2015/12/31"[PDat]) AND Humans[Mesh])))*
3. ***Clostridium:*** *(((diarrhoea[title] OR diarrhoea[MeSH Terms] OR diarrhoea[title] OR diarrhoea[MeSH Terms] OR gastroenteritis[title] OR gastroenteritis[MeSH Terms] OR gastro-enteritis[title] AND clostridium difficile[title/abstract] OR c. difficile[title/abstract] OR c. difficile[Mesh Terms] OR clostridium difficile[Mesh Terms]) AND ((aetiolog*[title/abstract] OR aetiology[MeSH Terms] OR cause[title/abstract] OR pathogen[title/abstract])) NOT ((colitis[title/abstract] OR enterocolitis[title/abstract] OR inflammatory bowel[title/abstract] OR irritable[title/abstract]OR Crohn*[title/abstract] OR HIV[title] OR treatment[title] OR therapy[title])) NOT ((appendicitis[title/abstract] OR esophag*[title/abstract] OR surger*[title/abstract] OR gastritis[title/abstract] OR liver[title/abstract] OR case report[title] OR case-report[title] OR therapy[title] OR treatment[title])) AND (("1990/01/01"[PDat] : "2015/12/31"[PDat]) AND Humans[Mesh])))*

PRISMA Diagram for the diarrhoea and aetiology input data search. The Preferred Reporting Items for Systematic reviews and Meta-Analyses (PRISMA) provide a structure to track the number of sources and exclusions in systematic reviews.[18] The flowchart below shows the number of sources used in the diarrhoea literature review and population-representative surveys and used in the non-fatal diarrhoea modelling and aetiologic attribution. The chart below shows the update for GBD 2015 and represents sources identified between January 1, 2012 and May 6, 2015.

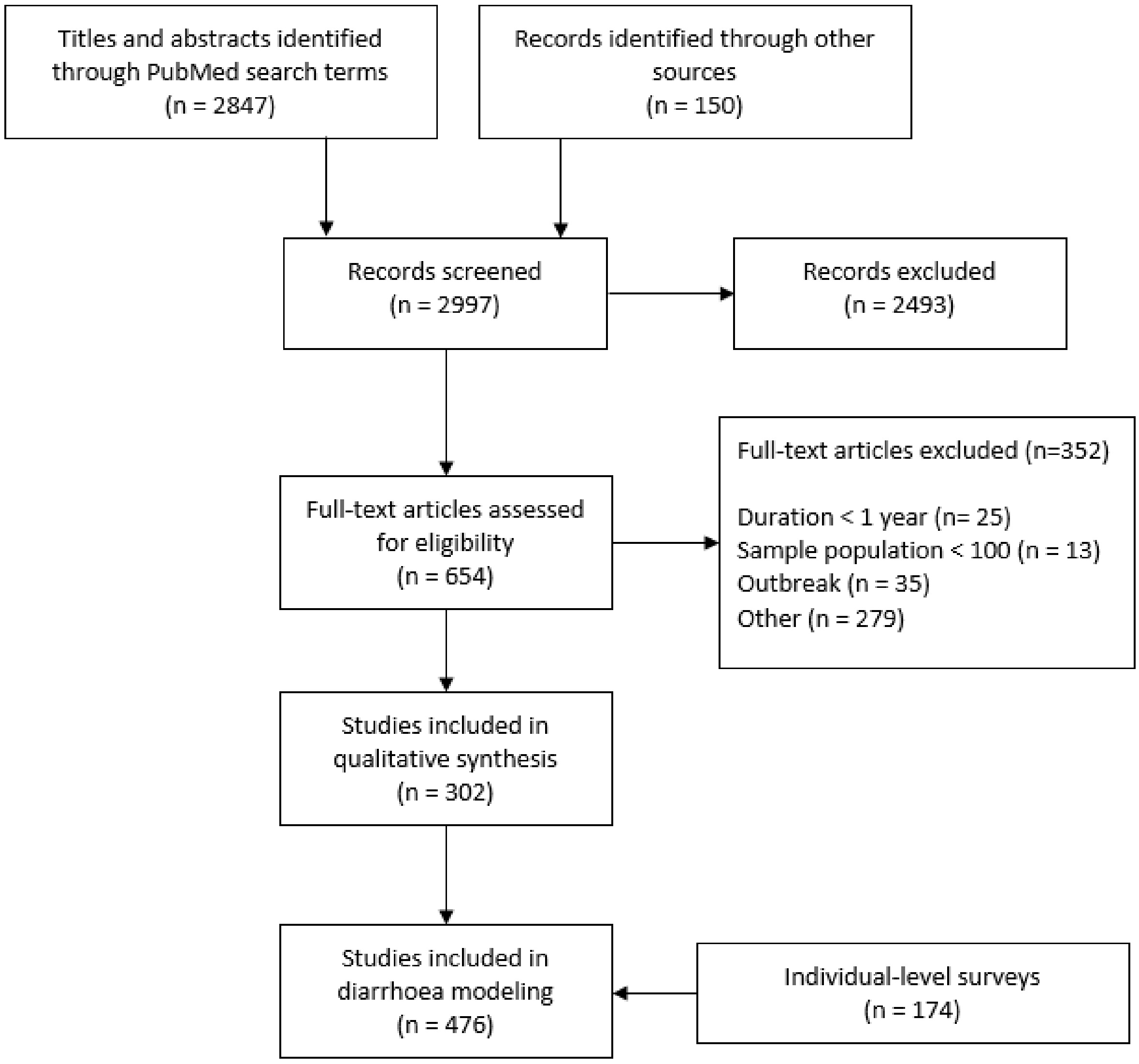

Appendix Figure 7. Geographic distribution of aetiology data. The number of data points that inform aetiologic attribution models by country and by aetiology is shown. White indicates no data points.

Number of data points used in Adenovirus Proportion Modeling (331)

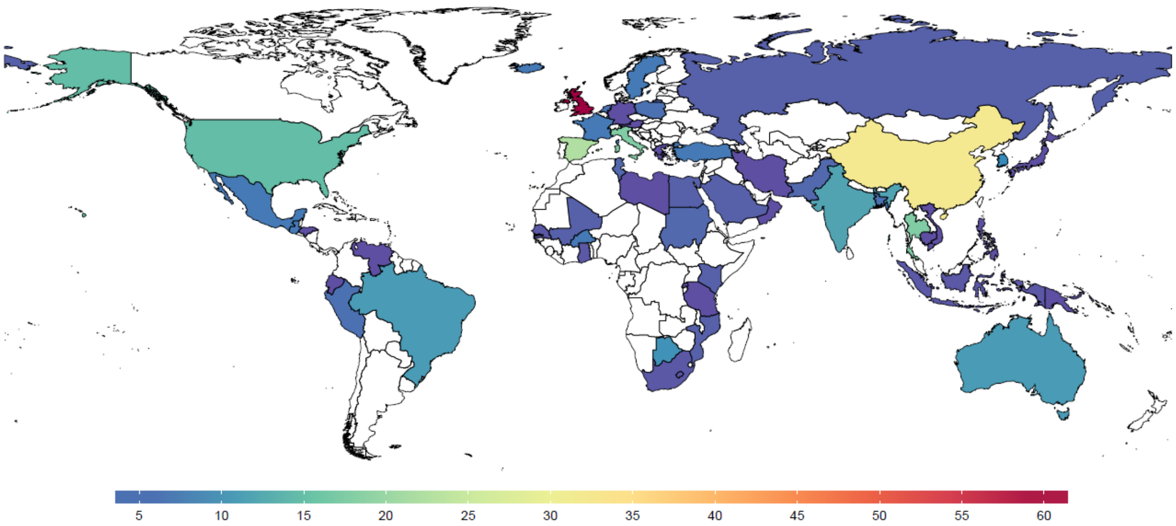

Number of data points used in Aeromonas Proportion Modeling (116)

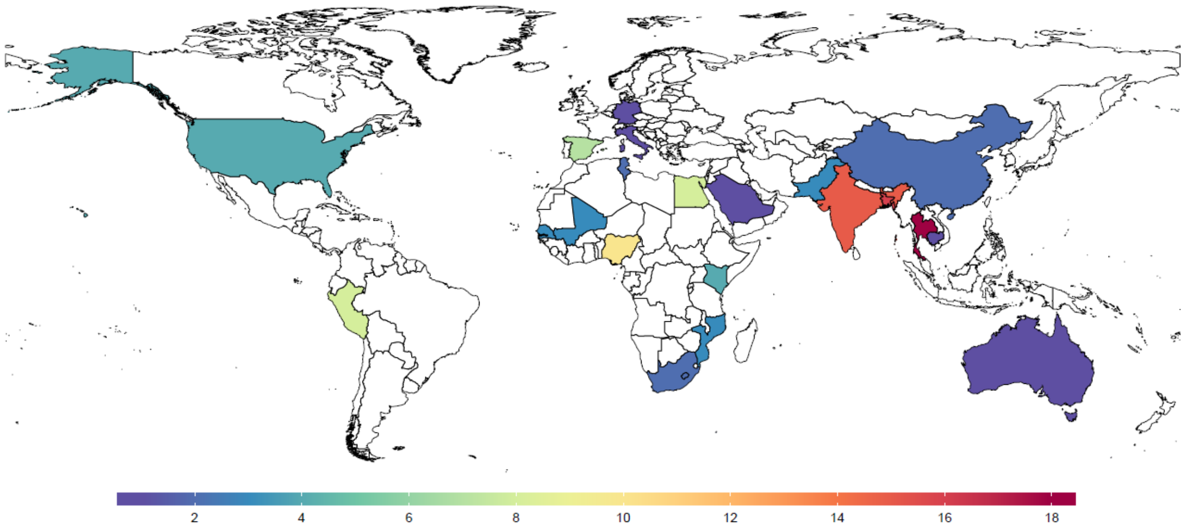

Number of data points used in Amoebiasis Proportion Modeling (204)

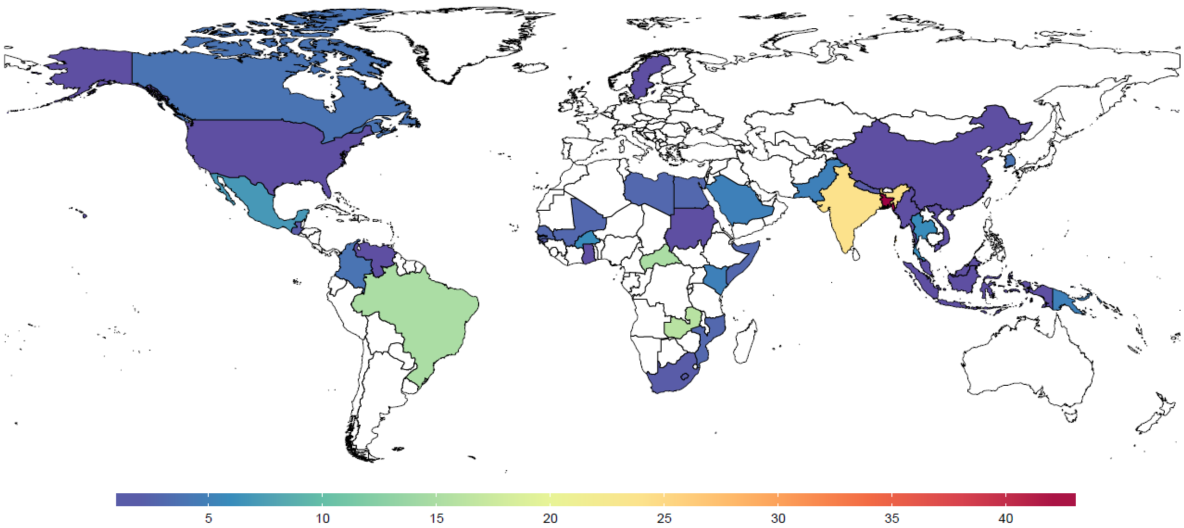

Number of data points used in Campylobacter enteritis Proportion Modeling (482)

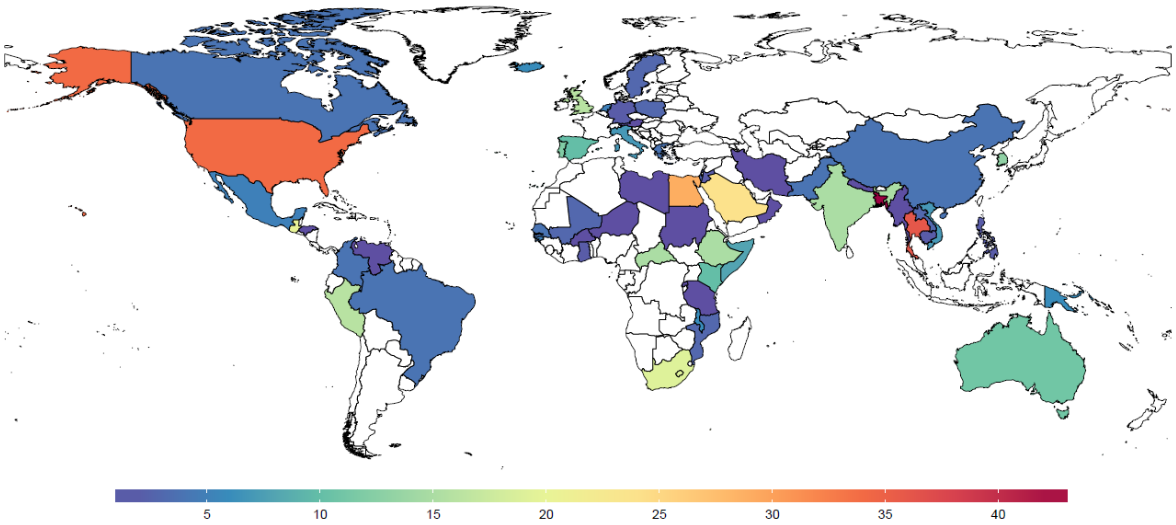

Number of data points used in Cholera O1 serogroup Proportion Modeling (2204)

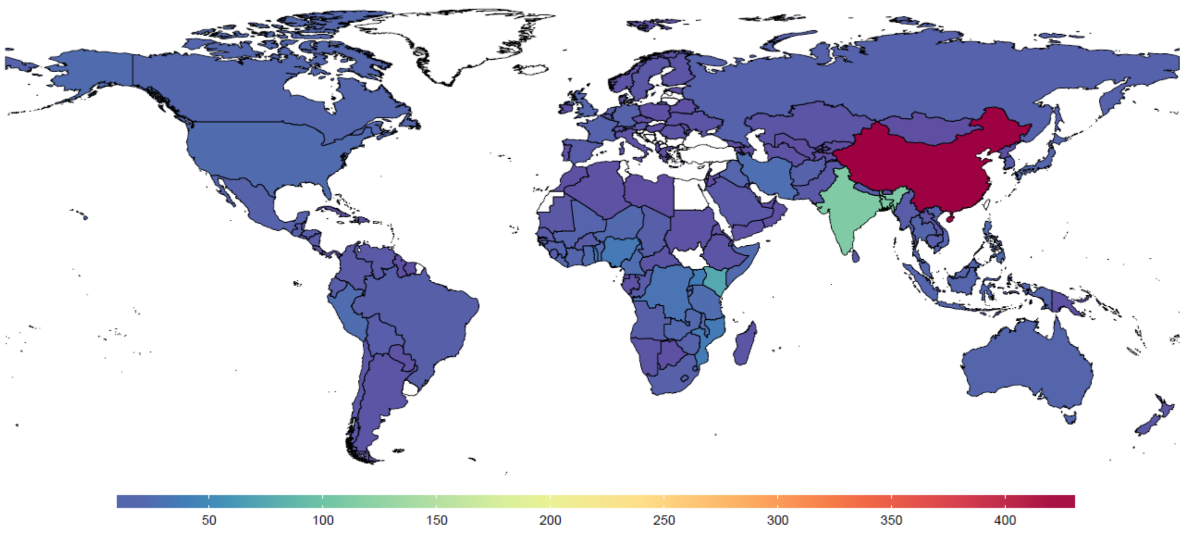

Number of data points used in Clostridium difficile Modeling (19,219)

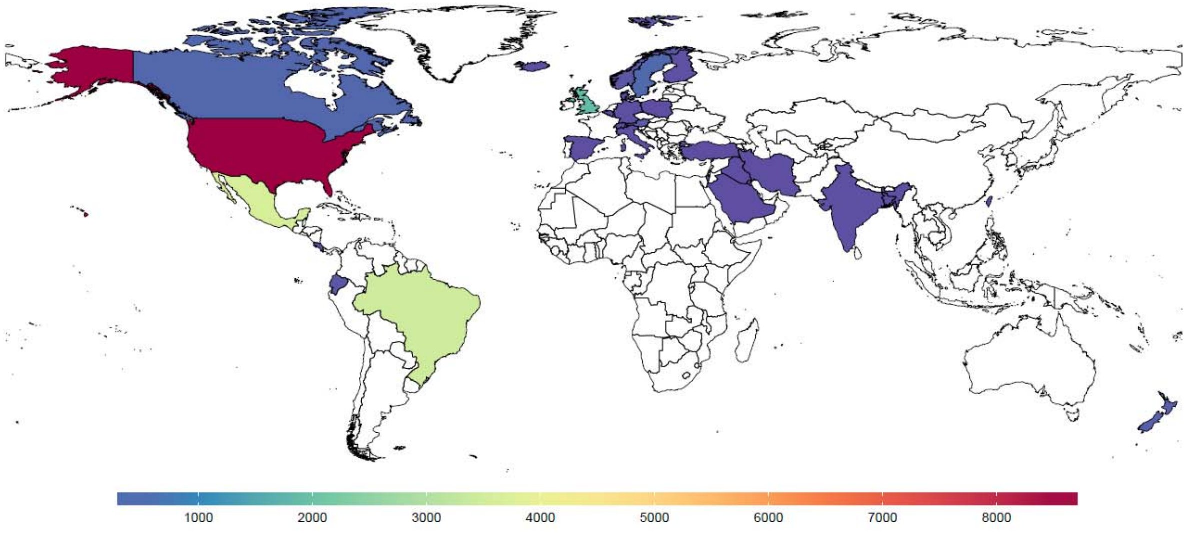

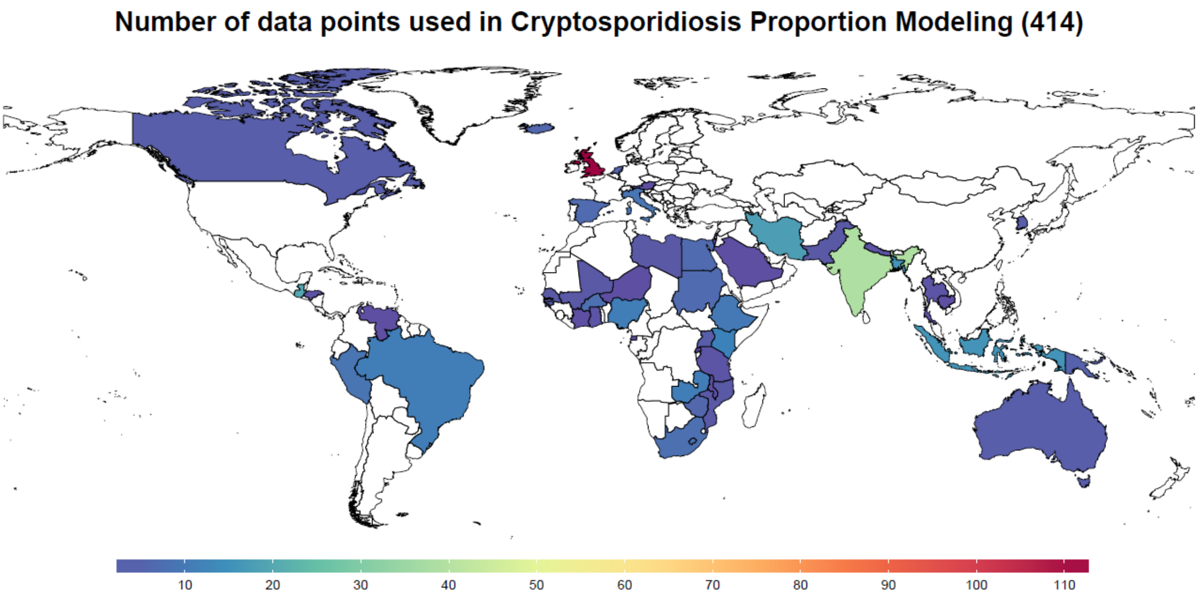
Number of data points used in Cryptosporidiosis Proportion Modeling (414)
10
20
30
40
50
60
70
80
90
100
110

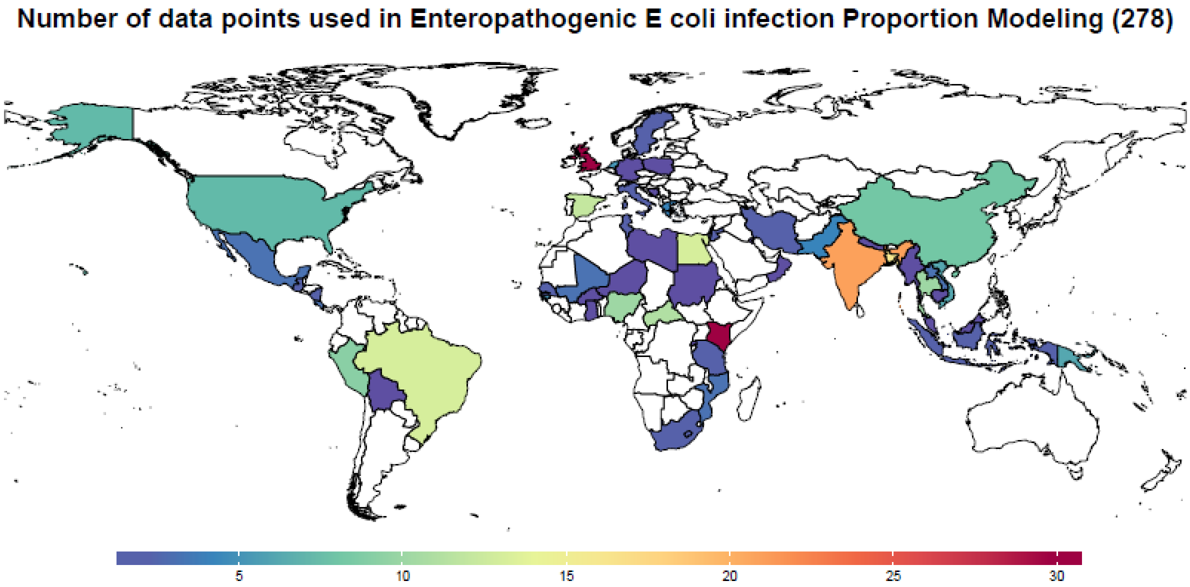
Number of data points used in Enteropathogenic E coli infection Proportion Modeling (278)
5
10
15
20
25
30

Number of data points used in Enterotoxigenic E coli infection Proportion Modeling (419)

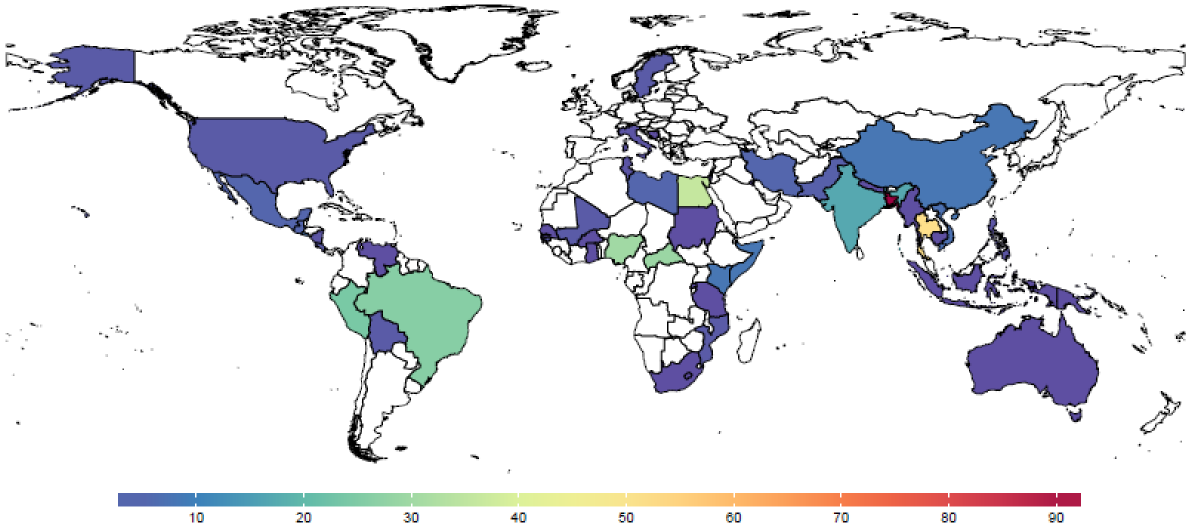

Number of data points used in Norovirus Proportion Modeling (393)

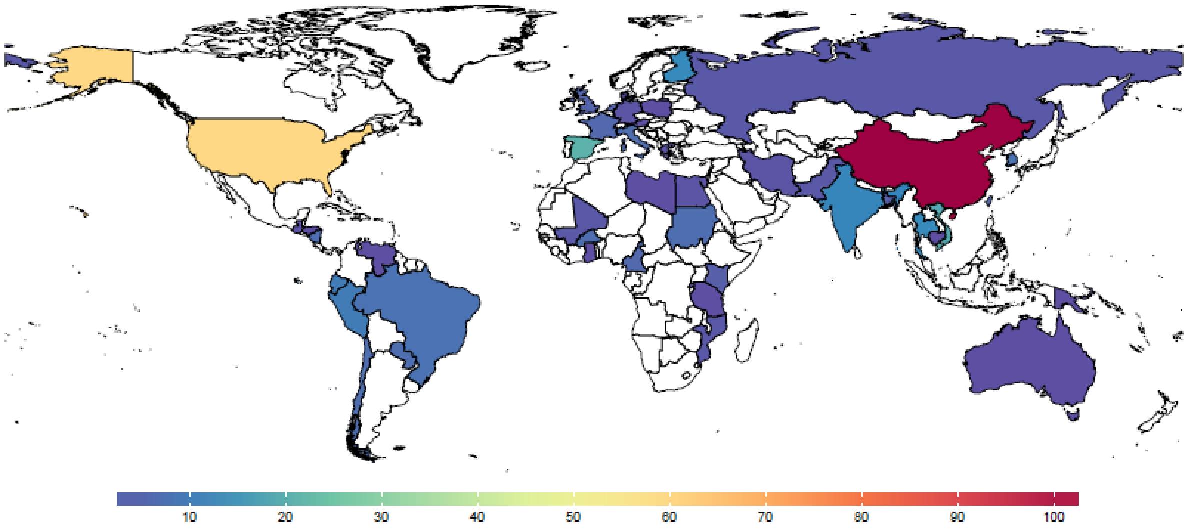

Number of data points used in Other salmonella infections Proportion Modeling (644)

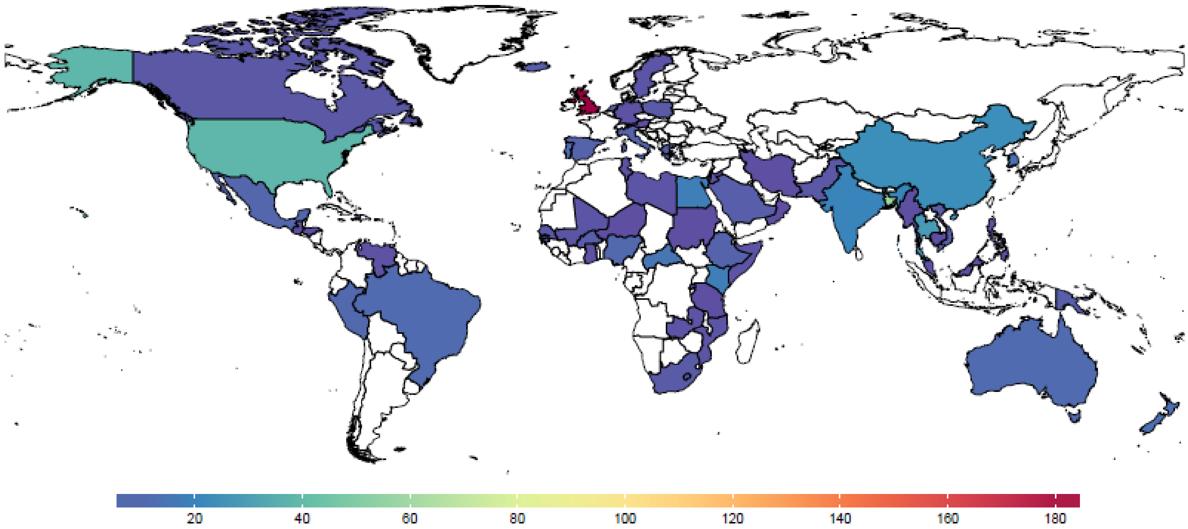

Number of data points used in Rotaviral enteritis Proportion Modeling (2154)

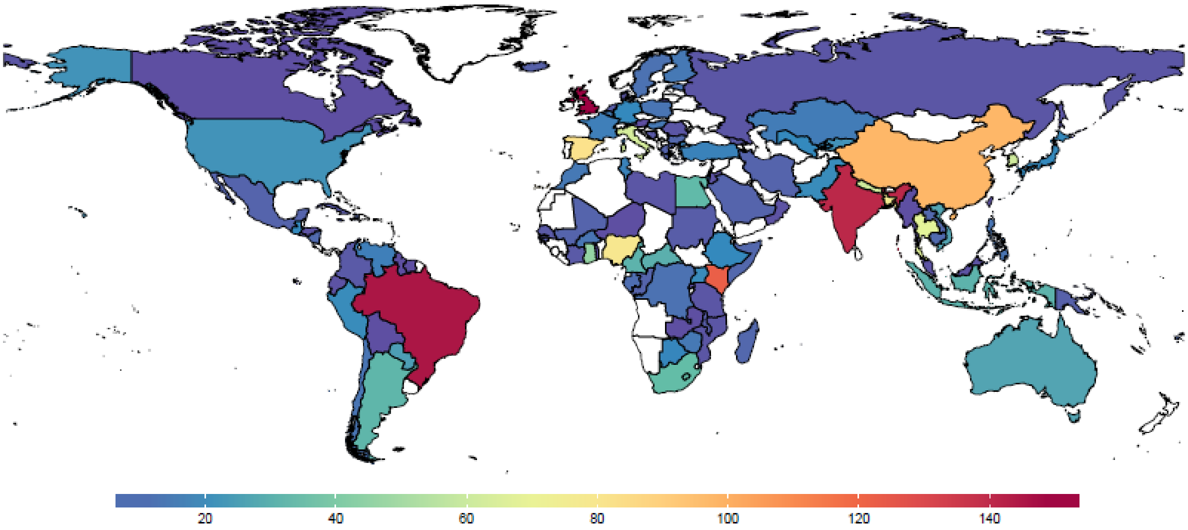

Number of data points used in Shigellosis Proportion Modeling (603)

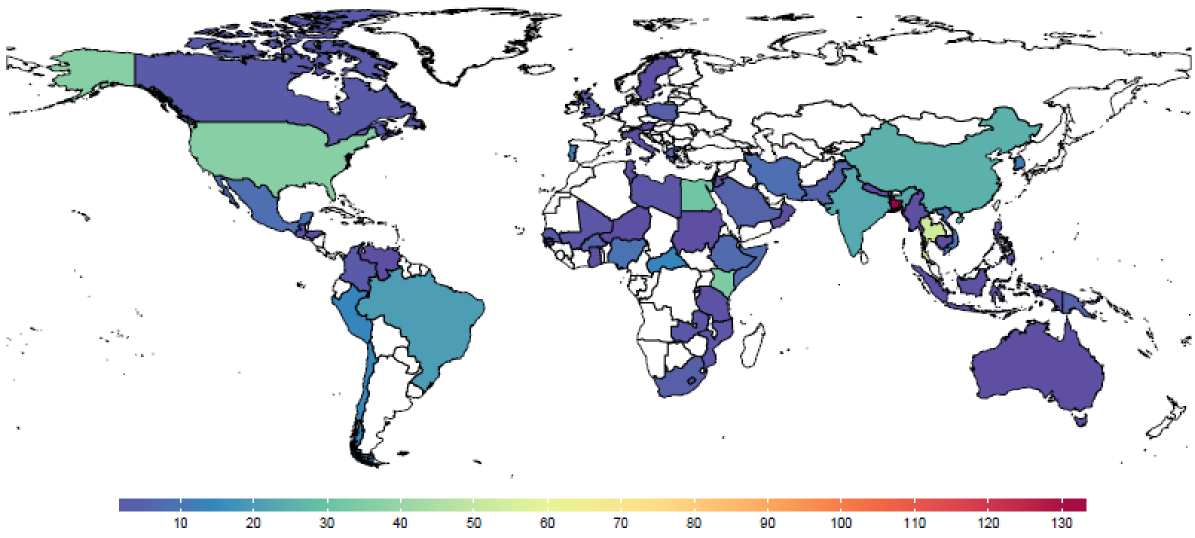

Comparison with GBD 2013

The differences in final estimates for diarrhoea mortality and morbidity between GBD 2013 and GBD 2015 are shown in **Appendix Figure 8.** One major change in estimation between GBD rounds is the introduction of subnational estimation in India and China. Modeling diarrhoea burden at the subnational level increases the number of input data points and improves resolution by allowing for greater within country variation. The number of under-5 deaths in Nigeria and Pakistan in 2010 deviates in GBD 2013 compared to GBD 2015 due mainly to changes in the availability (new data in Nigeria) and value (decrease in diarrhoea rate in Pakistan). These updates in Cause of Death data are especially pronounced in locations with sparse data (**Appendix Figure 2**).

Our findings showed that the attribution of diarrhoea aetiologies increased for most aetiologies compared to GBD 2013 (**Appendix Figure 9**). This is mainly due to two factors. First, we used the new qPCR molecular diagnostic for the detection of a given pathogen in GEMS compared to the conventional laboratory diagnostic methods used in GBD 2013. The qPCR method is more sensitive and specific than the conventional methods, such as bacterial culture or ELISA, which tends to change the odds ratios used in the attributable fraction estimation by correcting for the misclassification of pathogen.[11,12] Second, we used a correction factor to correct for false negatives and false positives of the prevalence of pathogens in diarrhoea patients and to make it comparable with the odds ratios from the qPCR diagnostic method. We corrected our modelled prevalence estimates for the imperfect sensitivity and specificity of the laboratory diagnostic results compared to qPCR since most studies reported diarrhoea based on previous diagnostics. Therefore, the correction for the prevalence of the pathogens widened our uncertainty of the final estimates. Although qPCR is a well-established diagnostic for diarrhoeal pathogens, the application of qPCR remains a novel methodology and further testing of the appropriate cut-offs for continuous measures of pathogen presence is needed.

The attributable fractions for *Aeromonas* and Amoebiasis (*Entamoeba histolytica*) were not statistically significant in children under 5 at the global level. Attributable fractions less than zero suggest that these pathogens are protective which is biologically implausible. We chose to report the full uncertainty interval, including negative values, to accurately portray the statistical result. The odds ratios of diarrhoea given the presence of *Aeromonas* and Amoebiasis were not statistically significant in children ages 0-1 and 1-2 years but were significant in the 2-5-year age group, highlighting that these microorganisms may not be significant pathogens across all age groups.

Appendix Figure 8. Comparing estimates for under-5 diarrhoea burden between GBD 2013[19] **and GBD 2015. A).** Under-5 diarrhoea deaths by country in 1990. **B).** Under-5 diarrhoea death by country in 2010. **C).** Under-5 diarrhoea years lived with disability (YLDs) by country in 1990. **D).** Under-5 diarrhoea YLDs by country in 2010.

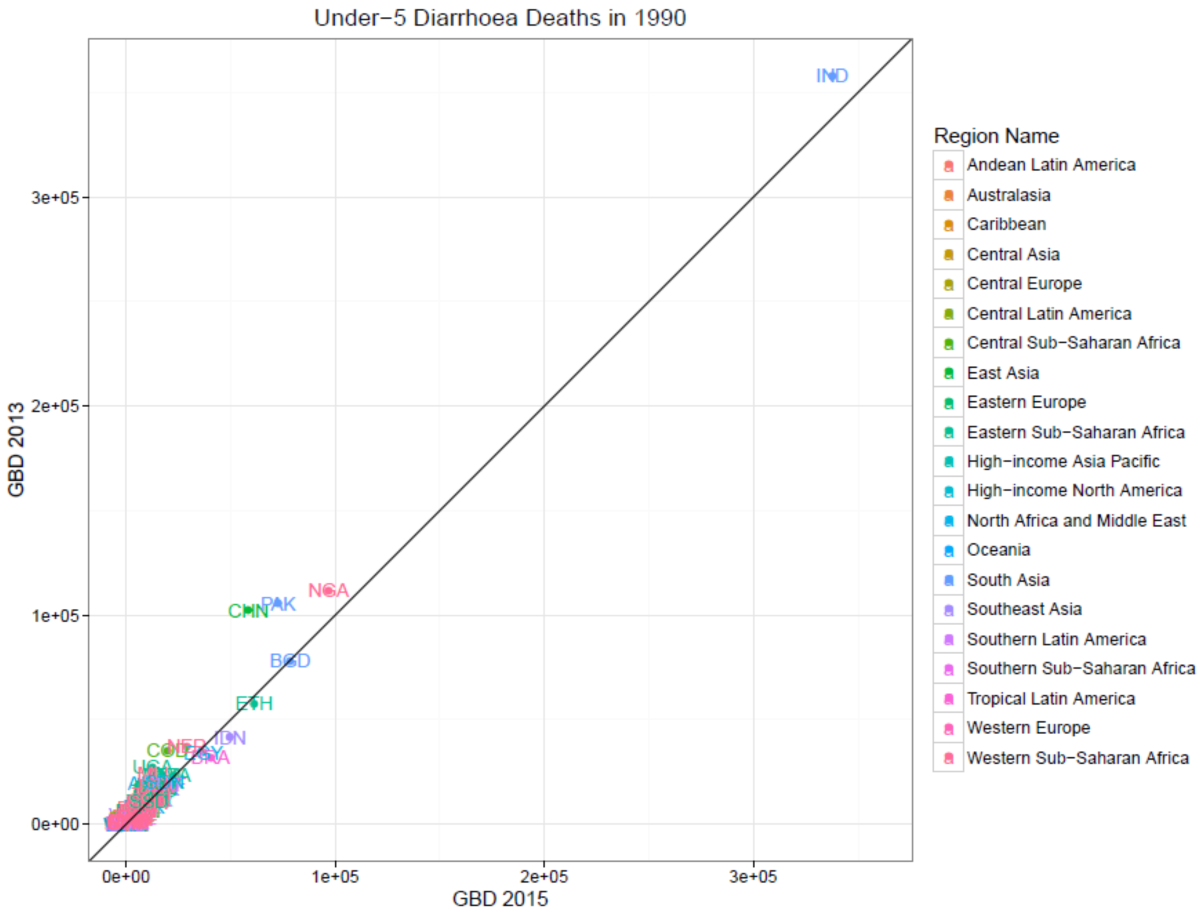

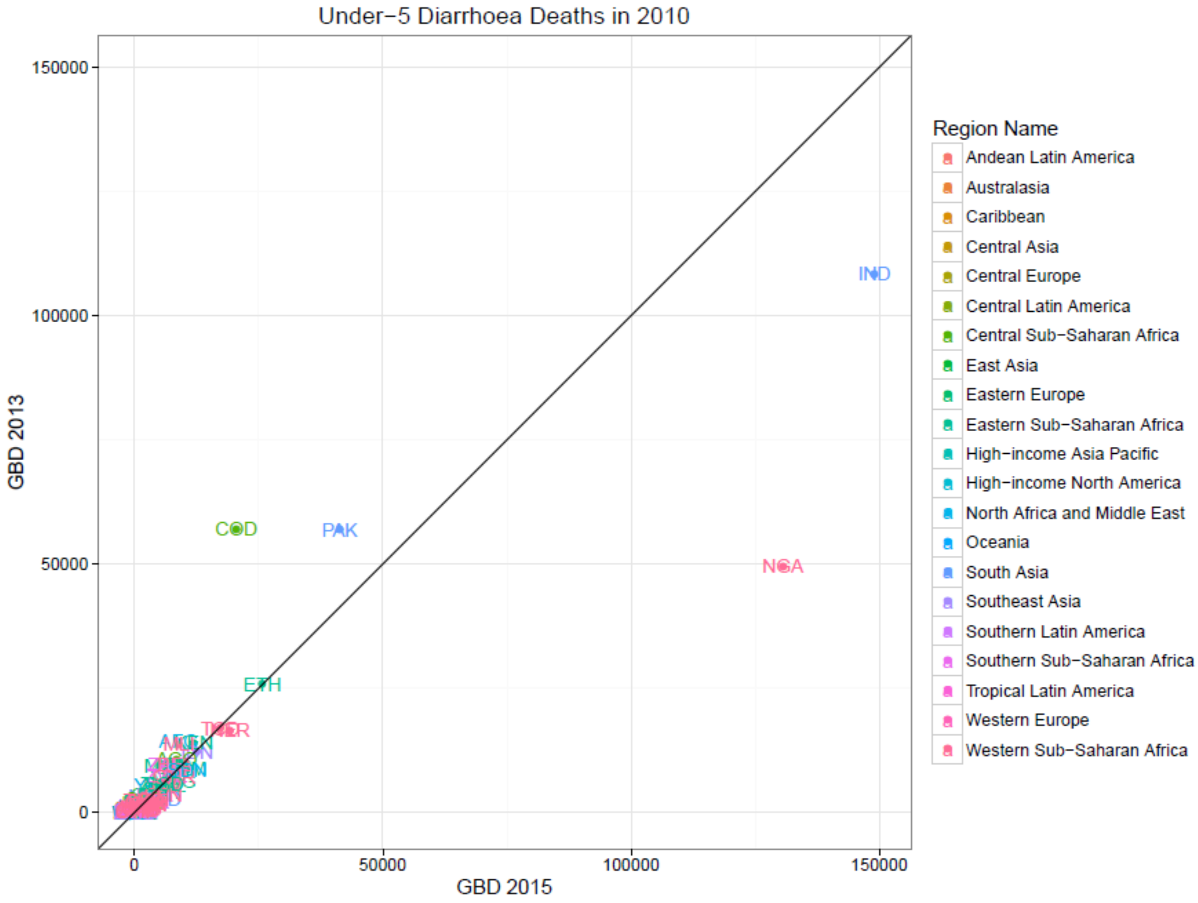
Under−5 Diarrhoea Deaths in 2010
GBD 2013
GBD 2015
150000
100000
50000
0
0
50000
100000
150000
IND
COD
PAK
NGA
ETH
Region Name
Andean Latin America
Australasia
Caribbean
Central Asia
Central Europe
Central Latin America
Central Sub−Saharan Africa
East Asia
Eastern Europe
Eastern Sub−Saharan Africa
High−income Asia Pacific
High−income North America
North Africa and Middle East
Oceania
South Asia
Southeast Asia
Southern Latin America
Southern Sub−Saharan Africa
Tropical Latin America
Western Europe
Western Sub−Saharan Africa

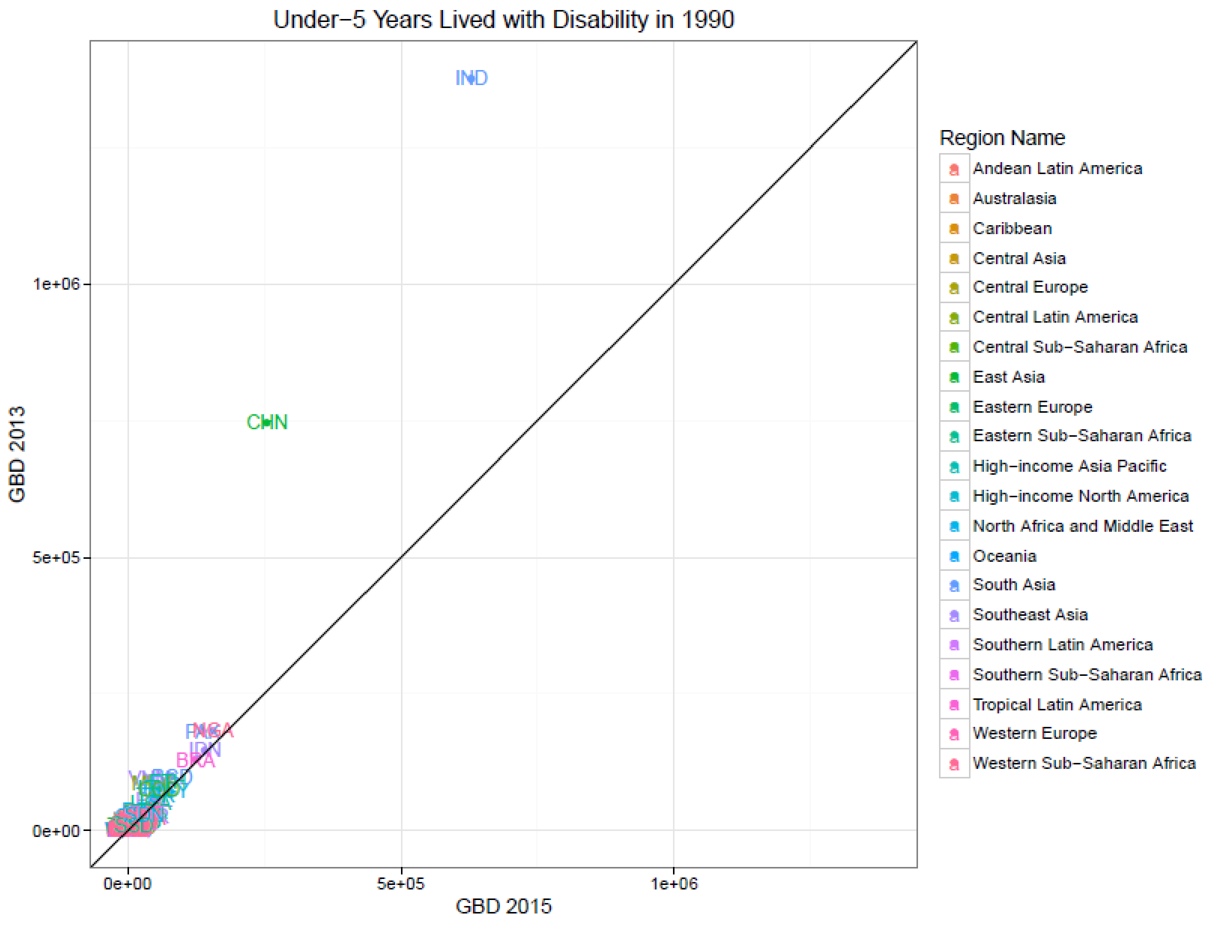

Under−5 Years Lived with Disability in 1990
GBD 2013
GBD 2015
1e+06
5e+05
0e+00
0e+00
5e+05
1e+06
IND
CHN
Region Name
Andean Latin America
Australasia
Caribbean
Central Asia
Central Europe
Central Latin America
Central Sub−Saharan Africa
East Asia
Eastern Europe
Eastern Sub−Saharan Africa
High−income Asia Pacific
High−income North America
North Africa and Middle East
Oceania
South Asia
Southeast Asia
Southern Latin America
Southern Sub−Saharan Africa
Tropical Latin America
Western Europe
Western Sub−Saharan Africa

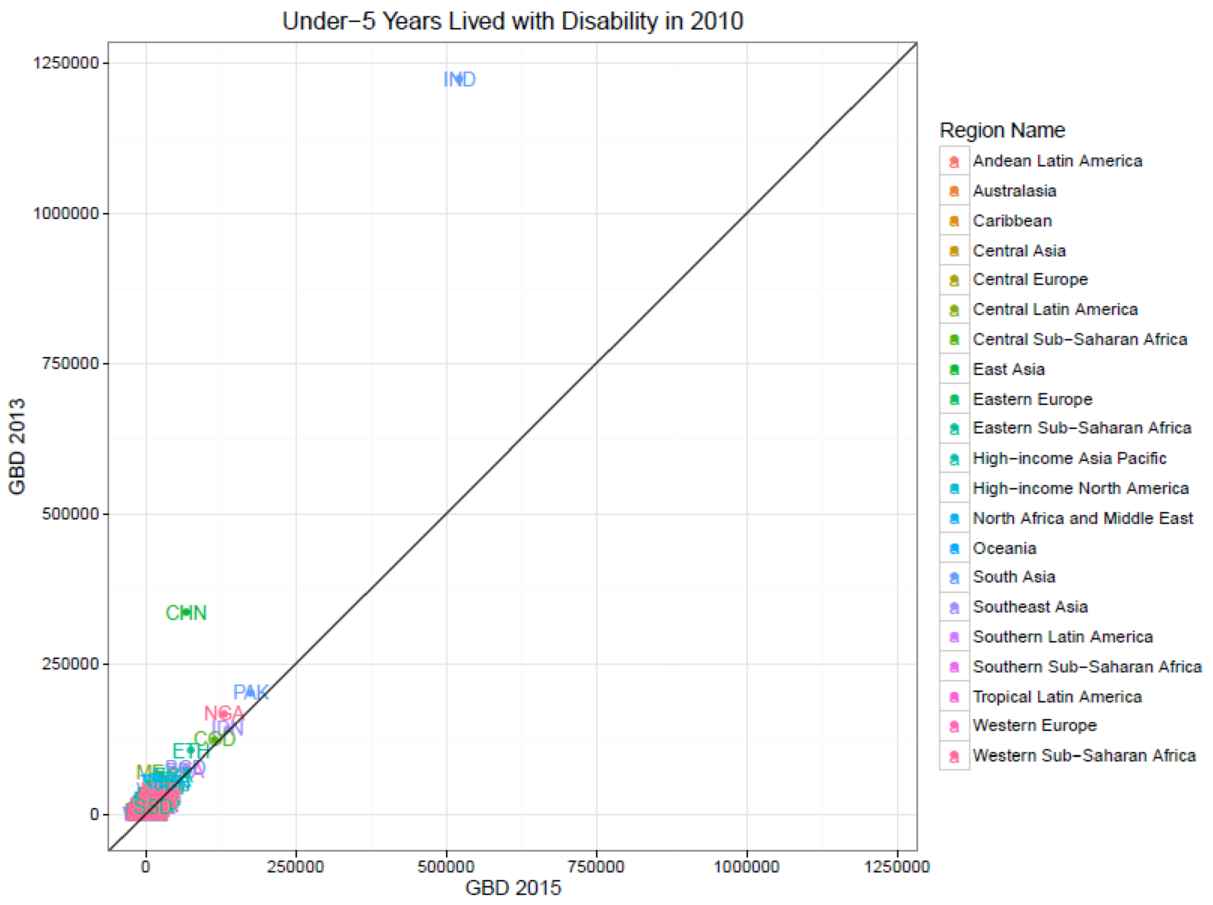

Under−5 Years Lived with Disability in 2010
GBD 2013
GBD 2015
1250000
1000000
750000
500000
250000
0
0
250000
500000
750000
1000000
1250000
IND
CHN
PAK
NGA
IDN
COD
ETH
Region Name
Andean Latin America
Australasia
Caribbean
Central Asia
Central Europe
Central Latin America
Central Sub−Saharan Africa
East Asia
Eastern Europe
Eastern Sub−Saharan Africa
High−income Asia Pacific
High−income North America
North Africa and Middle East
Oceania
South Asia
Southeast Asia
Southern Latin America
Southern Sub−Saharan Africa
Tropical Latin America
Western Europe
Western Sub−Saharan Africa

Appendix Figure 9. Aetiologic attribution to diarrhoea deaths among children under 5 years old in 2010. The top pie plot shows the results at the global level for GBD 2013[19] and the bottom pie plot shows the results at the global level for GBD 2015.

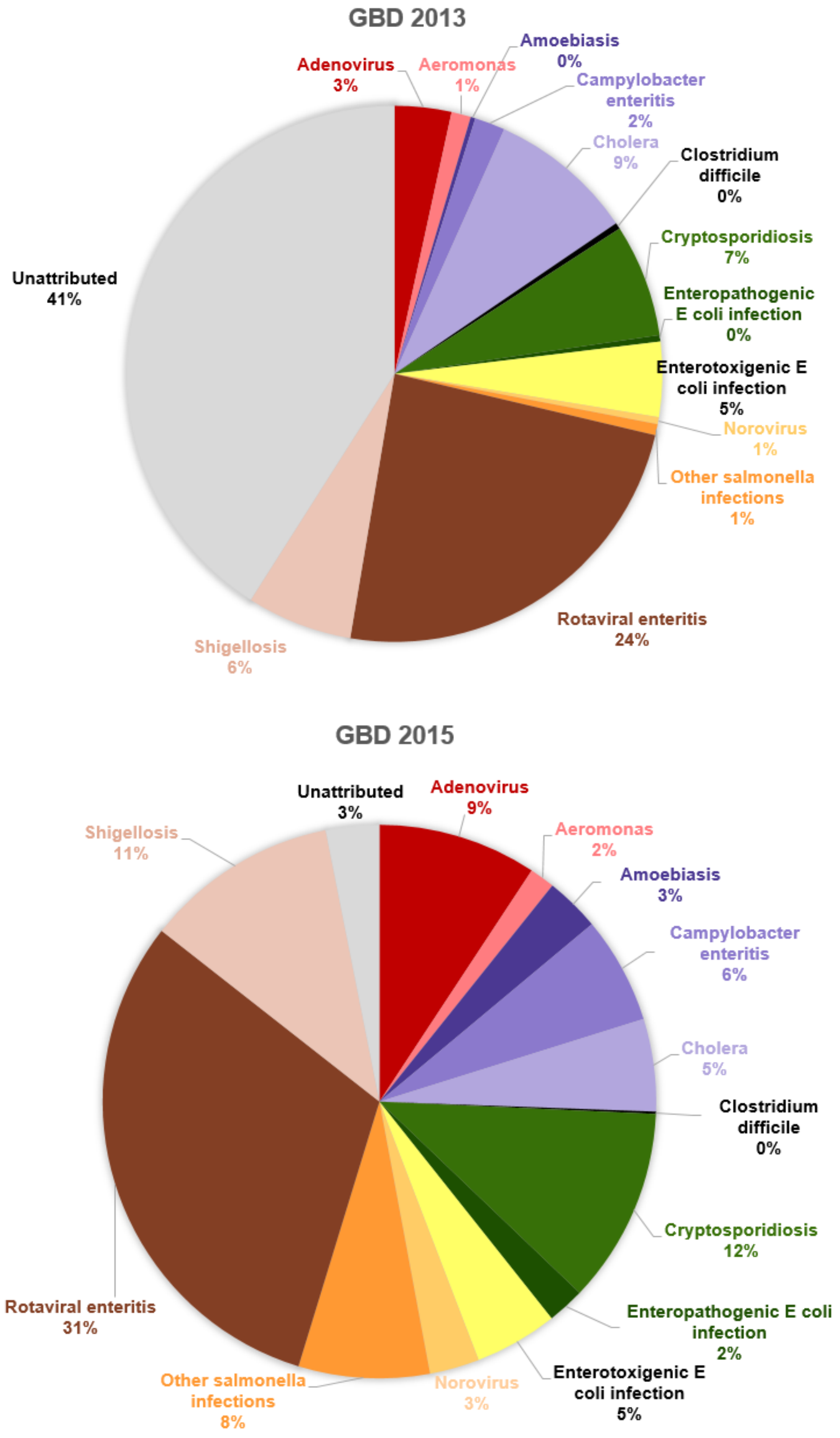

Comparison with WHO-MCEE

A comparison of the most recent estimates for aetiologic attribution to under-5 diarrhoea mortality between GBD 2015 and the CHERG-MCEE group is provided in the main text. The number of under-5 deaths by country in 2015 is shown in **Appendix Figure 10.**[20] Our estimates of diarrhoea mortality in children under 5 in 2015 (498,900) differ only slightly from those produced by the WHO Department of Evidence, Information and Research and the Maternal and Child Epidemiology Estimation (MCEE) group (526,000).[20] The main differences are in India and Nigeria.

GBD 2015 estimates for rotavirus are similar for the year 2010 but disparate for *Cryptosporidium* and *Shigella* which were considerably higher than those generated by CHERG (**Appendix Table 5**). Such differences may arise from varying methodological approaches, detailed elsewhere.[21] Two important differences are the application of molecular diagnostics in GBD 2015, which are not used by the CHERG-MCEE group, as well as the CHERG-MCEE group's categorical attribution of pathogen-specific diarrhoea proportion from hospitalised case data[22] whereas the GBD study uses a counterfactual approach to estimate population attributable fractions.

Appendix Table 5. Comparison of under-5 diarrhoea mortality estimates globally and by aetiology between GBD 2015 and the CHERG group. The values for GBD 2015 are for the year 2010 while the CHERG estimates are for the year 2011. The mortality estimates are presented as the number of deaths in thousands. CHERG did not produce estimates of diarrhoea mortality for *Aeromonas, Clostridium difficile,* or norovirus.

Etiology	CHERG	GBD 2015
Adenovirus	22<br>(12 to 37)	60<br>(22 to 126)
Aeromonas	-	9<br>(0 to 70)
Amoebiasis	1<br>(0 to 19)	20<br>(0 to 133)
Campylobacter	22<br>(11 to 50)	40<br>(11 to 78)
Cholera	9<br>(0 to 37)	34<br>(24 to 47)
Clostridium difficile	-	0.9<br>(0.8 to 1.0)
Cryptosporidiosis	14<br>(3 to 31)	76<br>(18 to 162)
Enteropathogenic *E coli*	79<br>(31 to 146)	14<br>(1 to 38)
Enterotoxigenic *E coli*	42<br>(20 to 76)	31<br>(14 to 57)
Norovirus	-	19<br>(8 to 45)
Salmonella	18<br>(10 to 30)	49<br>(16 to 108)
Rotavirus	197<br>(110 to 295)	199<br>(163 to 246)
Shigellosis	28<br>(12 to 53)	73<br>(36 to 127)
Total Deaths	712<br>(491 to 1049)	644<br>(586 to 708)

Appendix Figure 10. A comparison of under-5 diarrhoea deaths between GBD 2015 and the WHO-MCEE estimates.

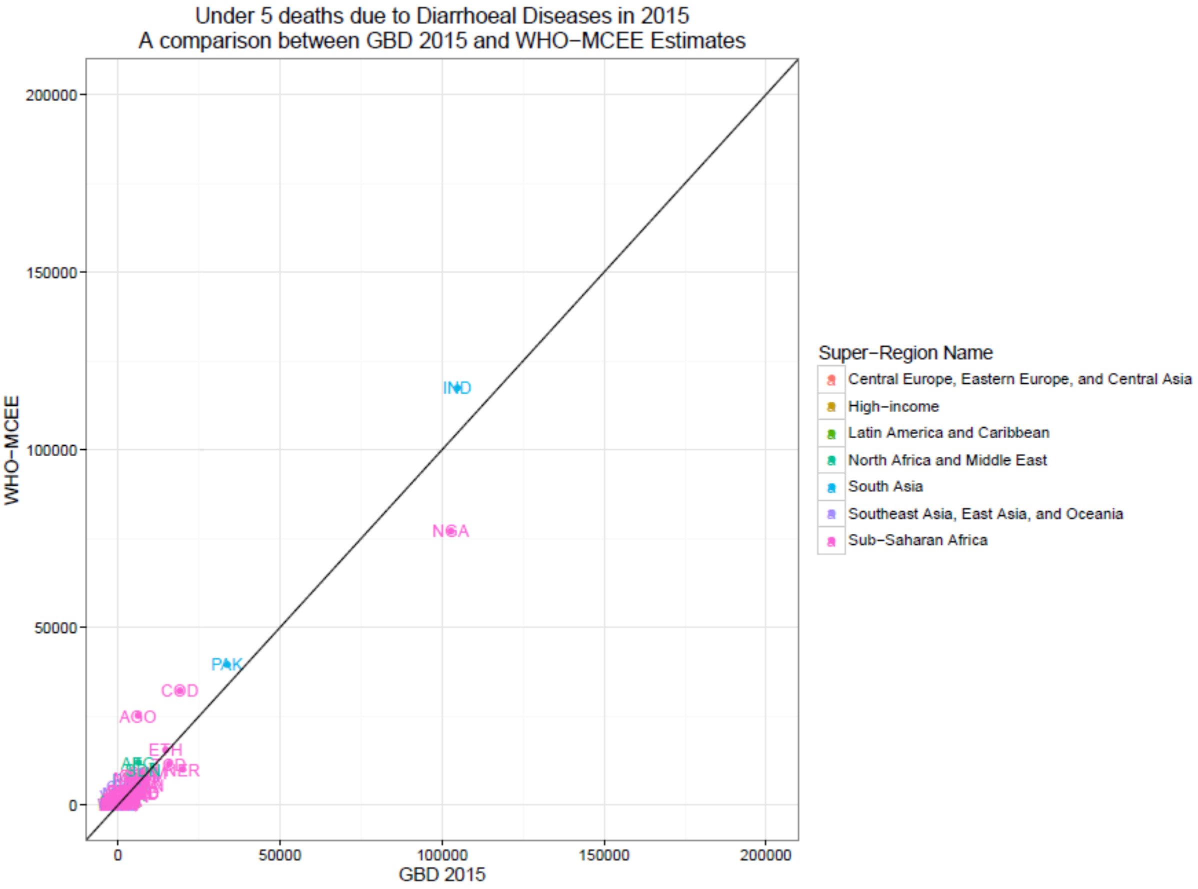

References

1 Naghavi M, Makela S, Foreman K, O'Brien J, Pourmalek F, Lozano R. Algorithms for enhancing public health utility of national causes-of-death data. *Popul Health Metr* 2010; **8**: 9.

2 GBD 2015 Mortality and Causes of Death Collaborators. Global, regional, and national life expectancy, all-cause and cause-specific mortality for 249 causes of death, 1980–2015: a systematic analysis for the Global Burden of Disease Study 2015. *Lancet* 2016; **388**: 1459–544.

3 Foreman KJ, Lozano R, Lopez AD, Murray CJ. Modeling causes of death: an integrated approach using CODEm. *Popul Health Metr* 2012; **10**: 1.

4 Foreman KJ, Lozano R, Lopez AD, Murray CJ. Modeling causes of death: an integrated approach using CODEm. *Popul Health Metr* 2012; **10**: 1.

5 GBD 2015 Mortality and Causes of Death Collaborators. Global, regional, and national life expectancy, all-cause mortality, and cause-specific mortality for 249 causes of death, 1980-2015: a systematic analysis for the Global Burden of Disease Study 2015. *Lancet Lond Engl* 2016; **388**: 1459–544.

6 GBD 2015 Risk Factors Collaborators. Global, regional and national comparative risk assessment of 79 behavioural, environmental and occupational, and metabolic risks or clusters of risks in 195 countries, 1990-2015: a systematic analysis for the Global Burden of Disease Study 2015. *Lancet* 2016; **388**: 1659–724.

7 GBD 2015 Disease and Injury Incidence and Prevalence Collaborators. Global, regional, and national incidence, prevalence, and years lived with disability for 310 diseases and injuries, 1990-2015: a systematic analysis for the Global Burden of Disease Study 2015. *Lancet* 2016; **388**: 1545–602.

8 World Health Organization: Department of Child and Adolescent Health and Development. Handbook Integrated Management of Childhood Illness. 2005.

9 Salomon JA, Haagsma JA, Davis A, *et al.* Disability weights for the Global Burden of Disease 2013 study. *Lancet Glob Health* 2015; **3**: e712-723.

10 Miettinen OS. Proportion of disease caused or prevented by a given exposure, trait or intervention. *Am J Epidemiol* 1974; **99**: 325–32.

11 Liu J, Kabir F, Manneh J, *et al.* Development and assessment of molecular diagnostic tests for 15 enteropathogens causing childhood diarrhoea: a multicentre study. *Lancet Infect Dis* 2014; **14**: 716–24.

12 Liu J, Gratz J, Amour C, *et al.* A laboratory-developed TaqMan Array Card for simultaneous detection of 19 enteropathogens. *J Clin Microbiol* 2013; **51**: 472–80.

13 Reiczigel J, Földi J, Ozsvári L. Exact confidence limits for prevalence of a disease with an imperfect diagnostic test. *Epidemiol Infect* 2010; **138**: 1674–8.

14 Rothman K, Greenland S, Lash T. Modern Epidemiology, Third Edition. Philadelphia: Lippincott Williams & Wilkins, 2008.

15 Platts-Mills JA, Operario DJ, Houpt ER. Molecular diagnosis of diarrhea: current status and future potential. *Curr Infect Dis Rep* 2012; **14**: 41–6.

16 Ochoa TJ, Barletta F, Contreras C, Mercado E. New insights into the epidemiology of enteropathogenic Escherichia coli infection. *Trans R Soc Trop Med Hyg* 2008; **102**: 852–6.

17 World Health Organization. Global Health Observatory data repository: Cholera. 2016. http://apps.who.int/gho/data/node.main.174?lang=en (accessed Aug 25, 2016).

18 Moher D, Liberati A, Tetzlaff J, Altman DG, Group TP. Preferred Reporting Items for Systematic Reviews and Meta-Analyses: The PRISMA Statement. *PLOS Med* 2009; **6**: e1000097.

19 GBD 2013 Mortality and Causes of Death Collaborators. Global, regional, and national age-sex specific all-cause and cause-specific mortality for 240 causes of death, 1990-2013: a systematic analysis for the Global Burden of Disease Study 2013. *Lancet Lond Engl* 2015; **385**: 117–71.

20 WHO. Estimates for 2000-2015. http://www.who.int/healthinfo/global_burden_disease/estimates_child_cod_2015/en/ (accessed Aug 25, 2016).

21 Kovacs SD, Mullholland K, Bosch J, *et al.* Deconstructing the differences: a comparison of GBD 2010 and CHERG's approach to estimating the mortality burden of diarrhea, pneumonia, and their etiologies. *BMC Infect Dis* 2015; **15**: 16.

22 Lanata CF, Fischer-Walker CL, Olascoaga AC, *et al.* Global causes of diarrheal disease mortality in children <5 years of age: a systematic review. *PloS One* 2013; **8**: e72788.
